# Safety, immunogenicity, and reactogenicity of BNT162b2 and mRNA-1273 COVID-19 vaccines given as fourth-dose boosters following two doses of ChAdOx1 nCoV-19 or BNT162b2 and a third dose of BNT162b2 (COV-BOOST): a multicentre, blinded, phase 2, randomised trial

**DOI:** 10.1016/S1473-3099(22)00271-7

**Published:** 2022-08

**Authors:** Alasdair P S Munro, Shuo Feng, Leila Janani, Victoria Cornelius, Parvinder K Aley, Gavin Babbage, David Baxter, Marcin Bula, Katrina Cathie, Krishna Chatterjee, Kate Dodd, Yvanne Enever, Ehsaan Qureshi, Anna L Goodman, Christopher A Green, Linda Harndahl, John Haughney, Alexander Hicks, Agatha A van der Klaauw, Nasir Kanji, Vincenzo Libri, Martin J Llewelyn, Alastair C McGregor, Mina Maallah, Angela M Minassian, Patrick Moore, Mehmood Mughal, Yama F Mujadidi, Kyra Holliday, Orod Osanlou, Rostam Osanlou, Daniel R Owens, Mihaela Pacurar, Adrian Palfreeman, Daniel Pan, Tommy Rampling, Karen Regan, Stephen Saich, Tanveer Bawa, Dinesh Saralaya, Sunil Sharma, Ray Sheridan, Emma C Thomson, Shirley Todd, Chris Twelves, Robert C Read, Sue Charlton, Bassam Hallis, Mary Ramsay, Nick Andrews, Teresa Lambe, Jonathan S Nguyen-Van-Tam, Matthew D Snape, Xinxue Liu, Saul N Faust, COV-BOOST study group, Alasdair P S Munro, Shuo Feng, Leila Janani, Victoria Cornelius, Parvinder K Aley, Gavin Babbage, David Baxter, Marcin Bula, Katrina Cathie, Krishna Chatterjee, Kate Dodd, Yvanne Enever, Ehsaan Qureshi, Anna L Goodman, Christopher A Green, Linda Harndahl, John Haughney, Alexander Hicks, Agatha A van der Klaauw, Nasir Kanji, Vincenzo Libri, Martin J Llewelyn, Alastair C McGregor, Angela M Minassian, Patrick Moore, Mehmood Mughal, Yama F Mujadidi, Kyra Holliday, Orod Osanlou, Rostam Osanlou, Daniel R Owens, Mihaela Pacurar, Adrian Palfreeman, Daniel Pan, Tommy Rampling, Karen Regan, Stephen Saich, Tanveer Bawa, Dinesh Saralaya, Sunil Sharma, Ray Sheridan, Mina Maallah, Emma C Thomson, Shirley Todd, Chris Twelves, Robert C Read, Sue Charlton, Bassam Hallis, Mary Ramsay, Nick Andrews, Teresa Lambe, Jonathan S Nguyen-Van-Tam, Matthew D Snape, Xinxue Liu, Saul N Faust, Andrew Riordan, Andrew Ustianowski, Chris Rogers, Kashyap Katechia, Alison Cooper, Andrew Freedman, Rachel Hughes, Lynne Grundy, Lona Tudor Jones, Elizabeth Harrison, Emma Snashall, Lewis Mallon, Katharine Burton, Kim Storton, Malathi Munusamy, Bridget Tandy, Akamino Egbo, Stephen Cox, Nabeela Nazir Ahmed, Anil Shenoy, Rachel Bousfield, Donna Wixted, Helen Gutteridge, Becky Mansfield, Christopher Herbert, Jennifer Murira, James Calderwood, Dominique Barker, Jacqueline Brandon, Hayley Tulloch, Suzie Colquhoun, Helen Thorp, Helen Radford, Julie Evans, Helena Baker, Jeanette Thorpe, Sally Batham, Jessica Hailstone, Rachael Phillips, Dileep Kumar, Fran Westwell, Ann Sturdy, Lara Barcella, Najwa Soussi, Mushiya Mpelembue, Sreena Raj, Rajni Sharma, Tumena Corrah, Laurence John, Ashley Whittington, Siobhan Roche, Lynda Wagstaff, Adam Farrier, Karen Bisnauthsing, Movin Abeywickrama, Niamh Spence, Alice Packham, Teona Serafimova, Suahil Aslam, Caitlin McGreevy, Alessandro Borca, Pamela DeLosSantosDominguez, Emily Palmer, Samantha Broadhead, Sadaf Farooqi, Jo Piper, Rowena Weighell, Lorinda Pickup, Djamila Shamtally, Jason Domingo, Evgenia Kourampa, Colin Hale, Jennifer Gibney, Michael Stackpoole, Zalina Rashid-Gardner, Rebecca Lyon, Chloe McDonnell, Christine Cole, Anna Stewart, Gillian McMillan, Mary Savage, Helen Beckett, Chantelle Moorbey, Amisha Desai, Claire Brown, Kush Naker, Karishma Gokani, Charlotte Trinham, Charlette Sabine, Sophie Moore, Steve Hurdover, Edwin Justice, Megan Stone, Emma Plested, Carla Ferreira Da Silva, Rachel White, Hannah Robinson, Iain Turnbull, Gertraud Morshead, Rachael Drake-Brockman, Catherine Smith, Grace Li, Mwila Kasanyinga, Elizabeth A Clutterbuck, Sagida Bibi, Michael Singh, Trishna Champaneri, Margaret Irwin, Mohammed Khan, Alicia Kownacka, Martha Nabunjo, Carol Osuji, John Hladkiwskyj, Dominic Galvin, Gita Patel, Jacques Grierson, Samantha Males, Krishna Askoolam, Joshua Barry, Johanna Mouland, Beverley Longhurst, Maria Moon, Beth Giddins, Carlota Pereira Dias Alves, Leah Richmond, Christine Minnis, Sonia Baryschpolec, Scott Elliott, Lauren Fox, Victoria Graham, Natalie Baker, Kerry Godwin, Karen Buttigieg, Chanice Knight, Phillip Brown, Paminder Lall, Imam Shaik, Emily Chiplin, Emily Brunt, Stephanie Leung, Lauren Allen, Steve Thomas, Sara Fraser, Bea Choi, Jade Gouriet, Jonathan Perkins, Andrew Gowland, Jonathan Macdonald, John Paul Seenan, Igor Starinskij, Andrew Seaton, Erica Peters, Stephen Singh, Ben Gardside, Avril Bonnaud, Ceri Davies, Elizabeth Gordon, Samantha Keenan, Jane Hall, Suzanne Wilkins, Suzanne Tasker, Rob James, Ingrid Seath, Kelly Littlewood, Joseph Newman, Iryna Boubriak, Debbie Suggitt, Helen Haydock, Sara Bennett, Wiesia Woodyatt, Kerry Hughes, Judith Bell, Tricia Coughlan, Donald van Welsenes, Mohammed Kamal, Chris Cooper, Simon Tunstall, Nicholas Ronan, Rebecca Cutts, Tracey Dare, Yee Ting Nicole Yim, Sarah Whittley, Shama Hamal, Marivic Ricamara, Kirsty Adams, Holly Baker, Kimberley Driver, Nicola Turner, Todd Rawlins, Subarna Roy, Marta Merida-Morillas, Yukari Sakagami, Antonette Andrews, Lillian Goncalvescordeiro, Matthew Stokes, Wythehi Ambihapathy, Joanne Spencer, Nina Parungao, Lisa Berry, James Cullinane, Laura Presland, Amy Ross Russell, Sarah Warren, Jonathan Baker, Abigail Oliver, Amanda Buadi, Kim Lee, Louise Haskell, Rossana Romani, Ian Bentley, Tim Whitbred, Simon Fowler, John Gavin, Alan Magee, Tara Watson, Kari Nightingale, Phedra Marius, Eloise Summerton, Emily Locke, Thomas Honey, Aidan Lingwood, Anastasia de la Haye, Ryan Stephen Elliott, Karen Underwood, Mikayala King, Sharon Davies-Dear, Emily Horsfall, Olivia Chalwin, Holly Burton, Christopher J Edwards, Benjamin Welham, Kim Appleby, Emily Dineen, Sarah Garrahy, Fran Hall, Eleni Ladikou, Dee Mullan, Daniel Hansen, Marion Campbell, Filipa Dos Santos, Nicki Lakeman, Debbie Branney, Luke Vamplew, Alison Hogan, Jorden Frankham, Martin Wiselka, Denny Vail, Victoria Wenn, Valerie Renals, Kate Ellis, Jessica Lewis-Taylor, Haniah Habash-Bailey, Javier Magan, Anna Hardy

**Affiliations:** a NIHR Southampton Clinical Research Facility and Biomedical Research Centre, University Hospital Southampton NHS Foundation Trust, Southampton, UK; b Faculty of Medicine and Institute for Life Sciences, University of Southampton, Southampton, UK; c Oxford Vaccine Group, Department of Paediatrics, University of Oxford, Oxford, UK; d Jenner Institute, Nuffield Department of Medicine, University of Oxford, Oxford, UK; e Imperial Clinical Trials Unit, Imperial College London, London, UK; f NIHR Oxford Biomedical Research Centre, Oxford, UK; g Stockport NHS Foundation Trust, Stockport, UK; h NIHR Liverpool and Broadgreen Clinical Research Facility, Liverpool, UK; i NIHR Cambridge Clinical Research Facility, Cambridge University Hospitals NHS Foundation Trust, Cambridge, UK; j PHARMExcel, Welwyn Garden City, UK; k NIHR/Wellcome Clinical Research Facility, University Hospitals Birmingham NHS Foundation Trust, Birmingham, UK; l Department of Infection, Guy's and St Thomas' NHS Foundation Trust, London, UK; m MRC Clinical Trials Unit, University College London, London, UK; n Portsmouth Hospitals University NHS Trust, Portsmouth, UK; o Queen Elizabeth University Hospital, NHS Greater Glasgow and Clyde, Glasgow, UK; p Wellcome-MRC Institute of Metabolic Science, Department of Clinical Biochemistry, University of Cambridge, Cambridge, UK; q NIHR UCLH Clinical Research Facility and NIHR UCLH Biomedical Research Centre, University College London Hospitals NHS Foundation Trust, London, UK; r University Hospitals Sussex NHS Foundation Trust, Brighton, UK; s Department of Infectious Diseases and Tropical Medicine, London Northwest University Healthcare, London, UK; t The Adam Practice, Poole, UK; u NIHR Leeds Clinical Research Facility, Leeds Teaching Hospitals Trust and University of Leeds, Leeds, UK; v Public Health Wales, Betsi Cadwaladr University Health Board, Bangor University, Bangor, UK; w University of Liverpool, Liverpool, UK; x University Hospitals of Leicester NHS Trust, University of Leicester, Leicester, UK; y Bradford Institute for Health Research, Bradford Teaching Hospitals NHS Foundation Trust, Bradford, UK; z Royal Devon and Exeter Hospital NHS Foundation Trust, Exeter, UK; aa MRC-University of Glasgow Centre for Virus Research, Glasgow, UK; ab UK Health Security Agency, Porton Down, Porton, UK; ac UK Health Security Agency, Colindale, London, UK; ad Division of Epidemiology and Public Health, University of Nottingham School of Medicine, University of Nottingham, Nottingham, UK

## Abstract

**Background:**

Some high-income countries have deployed fourth doses of COVID-19 vaccines, but the clinical need, effectiveness, timing, and dose of a fourth dose remain uncertain. We aimed to investigate the safety, reactogenicity, and immunogenicity of fourth-dose boosters against COVID-19.

**Methods:**

The COV-BOOST trial is a multicentre, blinded, phase 2, randomised controlled trial of seven COVID-19 vaccines given as third-dose boosters at 18 sites in the UK. This sub-study enrolled participants who had received BNT162b2 (Pfizer-BioNTech) as their third dose in COV-BOOST and randomly assigned them (1:1) to receive a fourth dose of either BNT162b2 (30 μg in 0·30 mL; full dose) or mRNA-1273 (Moderna; 50 μg in 0·25 mL; half dose) via intramuscular injection into the upper arm. The computer-generated randomisation list was created by the study statisticians with random block sizes of two or four. Participants and all study staff not delivering the vaccines were masked to treatment allocation. The coprimary outcomes were safety and reactogenicity, and immunogenicity (anti-spike protein IgG titres by ELISA and cellular immune response by ELISpot). We compared immunogenicity at 28 days after the third dose versus 14 days after the fourth dose and at day 0 versus day 14 relative to the fourth dose. Safety and reactogenicity were assessed in the per-protocol population, which comprised all participants who received a fourth-dose booster regardless of their SARS-CoV-2 serostatus. Immunogenicity was primarily analysed in a modified intention-to-treat population comprising seronegative participants who had received a fourth-dose booster and had available endpoint data. This trial is registered with ISRCTN, 73765130, and is ongoing.

**Findings:**

Between Jan 11 and Jan 25, 2022, 166 participants were screened, randomly assigned, and received either full-dose BNT162b2 (n=83) or half-dose mRNA-1273 (n=83) as a fourth dose. The median age of these participants was 70·1 years (IQR 51·6–77·5) and 86 (52%) of 166 participants were female and 80 (48%) were male. The median interval between the third and fourth doses was 208·5 days (IQR 203·3–214·8). Pain was the most common local solicited adverse event and fatigue was the most common systemic solicited adverse event after BNT162b2 or mRNA-1273 booster doses. None of three serious adverse events reported after a fourth dose with BNT162b2 were related to the study vaccine. In the BNT162b2 group, geometric mean anti-spike protein IgG concentration at day 28 after the third dose was 23 325 ELISA laboratory units (ELU)/mL (95% CI 20 030–27 162), which increased to 37 460 ELU/mL (31 996–43 857) at day 14 after the fourth dose, representing a significant fold change (geometric mean 1·59, 95% CI 1·41–1·78). There was a significant increase in geometric mean anti-spike protein IgG concentration from 28 days after the third dose (25 317 ELU/mL, 95% CI 20 996–30 528) to 14 days after a fourth dose of mRNA-1273 (54 936 ELU/mL, 46 826–64 452), with a geometric mean fold change of 2·19 (1·90–2·52). The fold changes in anti-spike protein IgG titres from before (day 0) to after (day 14) the fourth dose were 12·19 (95% CI 10·37–14·32) and 15·90 (12·92–19·58) in the BNT162b2 and mRNA-1273 groups, respectively. T-cell responses were also boosted after the fourth dose (eg, the fold changes for the wild-type variant from before to after the fourth dose were 7·32 [95% CI 3·24–16·54] in the BNT162b2 group and 6·22 [3·90–9·92] in the mRNA-1273 group).

**Interpretation:**

Fourth-dose COVID-19 mRNA booster vaccines are well tolerated and boost cellular and humoral immunity. Peak responses after the fourth dose were similar to, and possibly better than, peak responses after the third dose.

**Funding:**

UK Vaccine Task Force and National Institute for Health Research.

## Introduction

With the emergence of highly transmissible SARS-CoV-2 variants, such as omicron (B.1.1.529), many high-income countries have rapidly deployed third doses of COVID-19 vaccines to their populations. Third-dose boosters increase humoral and cellular immunity^1^ and provide more short-term protection against symptomatic infection with variants of concern, including omicron,^2^, ^3^ compared with a two-dose schedule. However, protection against symptomatic infection wanes rapidly following the second^4^ and third^2^ doses of COVID-19 vaccines. As of March, 2022, some countries, such as Israel and Germany, started to offer fourth-dose booster vaccines to their populations, and the UK rolled out fourth doses for clinically vulnerable populations in April, 2022.^5^

Observational data from Israel have shown a boosting effect on immunogenicity^6^ and moderate protection against symptomatic infection from a fourth dose of mRNA COVID-19 vaccines administered approximately 4 months after a third dose.^7^, ^8^ The clinical need, timing, and dose of the fourth COVID-19 vaccine remain uncertain,^9^ as does the gain in vaccine effectiveness compared with a third dose. Given the urgent need for data to inform policy on additional booster doses, the COV-BOOST trial^1^ of third-dose booster vaccines for COVID-19 was extended to investigate the safety, reactogenicity, and immunogenicity of fourth-dose boosters against COVID-19 administered approximately 7 months following a third dose of BNT162b2 (Pfizer-BioNTech).

## Methods

### Study design and participants

The COV-BOOST trial is a multicentre, blinded, phase 2, randomised controlled trial^1^ done at 18 sites in the UK. For the main COV-BOOST study,^1^ we enrolled participants aged 30 years or older who had received two doses of BNT162b2 or ChAdOx1 nCoV-19 (Oxford-AstraZeneca) and randomly assigned them to receive either a third-dose booster of one of seven COVID-19 vaccines (in ten schedules) or a meningococcal vaccine control. Details of the main study design have been described previously,^1^ and the full inclusion and exclusion criteria can be found in the protocol (appendix 2 pp 36–38). The statistical analysis plan is provided as appendix 3. This study is a randomised sub-trial nested within the main COV-BOOST trial. Participants who received a third-dose BNT162b2 booster in the COV-BOOST trial during June, 2021, were eligible for inclusion in this sub-study unless they had a previous severe adverse reaction to mRNA vaccines or had acquired an additional COVID-19 vaccine outside of the study since enrolling. Based on site location and participant availability, a subset of participants (around 25 per group) were enrolled into an immunology cohort to collect cellular immunology samples at 14 days after the fourth dose. The trial was reviewed and approved by the UK National Health Service (NHS) Research Ethics Service (21/SC/0171). All participants provided written informed consent.

**Research in context**

**Evidence before this study**

We searched PubMed for randomised controlled trials in non-immunocompromised adults published between database inception and March 31, 2022, using the search terms “(COVID) AND (vaccin*) AND (booster OR fourth dose)” with no language restrictions. We identified no clinical trials including fourth-dose COVID-19 vaccine boosters. One observational study following fourth doses of full-dose BNT162b2 (Pfizer-BioNTech) or half-dose mRNA-1273 (Moderna) in Israel in people who had received three previous doses of BNT162b2 found that humoral immunity after the fourth dose was boosted above peak levels after the third dose. A preprint of a small observational study of fourth-dose boosters from Germany found a boost to humoral immunity from baseline and the activation of T cells, which was weakly correlated with serum anti-spike protein antibody titres.

**Added value of this study**

To our knowledge, this study is the first to report a randomised trial of fourth-dose COVID-19 boosters. These data suggest that, after a period of approximately 7 months following third-dose boosters with BNT162b2, an additional dose of a COVID-19 mRNA vaccine can boost humoral anti-spike protein IgG titres and cellular responses to, or higher than, levels seen at 28 days after a third dose. Some participants with high levels of humoral and cellular responses before the fourth dose had limited boosting from the fourth dose, indicating that there could be a vaccine-specific ceiling effect. There might be additional antibody and T-cell boosting from heterologous mRNA fourth vaccine doses.

**Implications of all the available evidence**

More than 6 months after third-dose boosters, fourth doses of COVID-19 mRNA vaccines provide large increases in anti-spike protein antibody titres, although these increases will probably wane rapidly, as has been observed after third doses. People with high antibody titres are unlikely to gain much boosting from additional doses. This study provides important data to guide policy makers who might be considering the deployment of further booster doses of COVID-19 vaccines to the clinically vulnerable or whole populations.

### Randomisation and masking

Eligible participants were randomly assigned (1:1) to receive either BNT162b2 or mRNA-1273 (Moderna) as a fourth dose. The computer-generated randomisation list was created by the study statisticians with random block sizes of two or four, and randomisation was done with the electronic data capture system REDCap (version 10.6.13) by trained site staff. Allocation concealment was maintained by REDCap, in which the final randomisation list was only accessible by the IT manager and trial statistician. Randomisation was stratified by the initial two-dose vaccine schedule (ChAdOx1 nCoV-19 plus ChAdOx1 nCoV-19 *vs* BNT162b2 plus BNT162b2), study site, age (<70 years *vs* ≥70 years), and cohort (general *vs* immunology). Participants, laboratory staff, and the clinical study team not delivering the vaccines, including those assessing adverse events, were masked to treatment allocation. Data analysts were not masked to treatment allocation. Participant masking was maintained by concealing randomisation pages, preparing vaccines out of sight, and applying masking tape to vaccine syringes to conceal dose, volume, and appearance.

### Procedures

Procedures for the main study have been previously described.^1^ Two COVID-19 vaccines were used in this sub-study. Both BNT162b2 and mRNA-1273 are lipid nanoparticle-formulated, nucleoside-modified mRNA vaccines encoding trimerised SARS-CoV-2 spike glycoprotein. Administered by appropriately trained trial staff at the trial sites, participants received either BNT162b2 (30 μg in 0·30 mL; full dose) or mRNA-1273 (50 μg in 0·25 mL; half dose) via intramuscular injection into the upper arm. Participants were observed for at least 15 min after vaccination.

Blood samples for immunogenicity were taken at day 0 (before the fourth dose), day 14 (after the fourth dose), and day 84. Immunological assays are described in appendix 1 (p 12). Briefly, we measured SARS-CoV-2 anti-spike protein IgG concentrations by ELISA (Nexelis; Laval, QC, Canada) for all participants at all timepoints and cellular immune responses by ELISpot (Oxford Immunotec; Abingdon, UK) at day 0 for all participants and at day 14 for the immunology cohort only. Anti-SARS-CoV-2 nucleocapsid IgG status was analysed at Porton Down, Public Health England, by an electrochemiluminescence immunoassay (Cobas platform, Elecsys assay; Roche Diagnostics; Rotkreuz, Switzerland). Safety endpoints were followed up by use of electronic diaries completed by participants daily for the first 7 days and then on an ad hoc basis and by direct solicitation in person at the day 14 follow-up visit. The study visits will be completed by May, 2022.

### Outcomes

The coprimary outcomes were the safety and reactogenicity, and immunogenicity, of fourth-dose booster vaccination with full-dose BNT162b2 or half-dose mRNA-1273. Safety was assessed by sites, reactogenicity was self-reported, and immunogenicity was assessed centrally by different commercial laboratories. Safety and reactogenicity were characterised by the occurrence of solicited local and systemic adverse events within 7 days of the fourth dose, unsolicited adverse events within 28 days of the fourth dose, medically attended adverse events up to 3 months following the fourth dose, adverse events of special interest, and serious adverse events. Serious adverse events and adverse events of special interest (appendix 2 p 73) were recorded throughout the study. The severity of clinical and laboratory adverse events was assessed according to scales based on the toxicity grading scales of the Food and Drug Administration for healthy adult volunteers enrolled in preventive vaccine clinical trials. Immunogenicity was defined as anti-spike protein IgG antibody titres (and live virus neutralising antibody titres, data for which are not reported here due to laboratory delays but will be reported at the first opportunity) for all participants and cellular immune responses for participants in the immunology cohort (appendix 1 p 12). To accelerate the data being available for policy decision making, and because maximum anti-spike protein IgG responses had been seen before day 28 following a third dose in the initial analysis,^1^ we used day 14 as the primary outcome timepoint. A secondary outcome was immunogenicity at day 84 following the fourth dose; because these assays have not yet been processed, we do not report this outcome and it will be reported elsewhere.

### Statistical analysis

Our aim was to investigate the boosting in immunological endpoints following two mRNA fourth-dose booster vaccines administered after ChAdOx1 nCoV-19 plus ChAdOx1 nCoV-19 plus BNT162b2 or BNT162b2 plus BNT162b2 plus BNT162b2 (the most commonly deployed COVID-19 vaccination schedules in the UK). As hypothesis testing between the two fourth-dose mRNA vaccines was not the primary aim of the main study, no power or formal sample size calculations were done.

Safety and reactogenicity were analysed in the per-protocol population, which comprised all participants who received a fourth-dose booster, regardless of their history of SARS-CoV-2 infection and anti-nucleocapsid IgG serostatus before the fourth dose. The proportions of participants with at least one severe (grades 3–4) or one severe or moderate (grades 2–4) adverse event are presented by initial vaccine schedules (ChAdOx1 nCoV-19 plus ChAdOx1 nCoV-19 *vs* BNT162b2 plus BNT162b2) by use of radial plots. Unsolicited adverse events were coded according to the Medical Dictionary for Regulatory Activities and tabulated at System Organ Class level across vaccine groups. Adverse events of special interest and serious adverse events are reported up to the data cutoff date of March 2, 2022, by line listing.

The primary immunogenicity outcomes were analysed in the modified intention-to-treat seronegative population, which comprised participants who received a fourth-dose booster, were seronegative before receiving the fourth dose (defined by the Roche Elecsys anti-SARS-CoV-2 nucleocapsid assay at all study visits before the fourth dose, including days 0 and 84 of the third dose and day 0 of the fourth dose), did not have SARS-CoV-2 infection before or within 7 days of the fourth dose (self-reported PCR or lateral flow tests following community testing), and had available endpoint data. The main analyses included all participants regardless of their initial two-dose vaccine schedules, with prespecified subgroup analyses split by the initial two-dose schedules (two doses of ChAdOx1 nCoV-19 *vs* BNT162b2) and age (<70 years *vs* ≥70 years).

In the immunogenicity analysis, we compared anti-spike protein IgG and T-cell responses at 14 days after the fourth dose versus 28 days after the third dose (data previously reported).^1^ For each paired data from one participant, the fold change was calculated by dividing immunogenicity values at day 14 after the fourth dose by those at day 28 after the third dose. As the fold change has a log-normal distribution, the geometric mean of the fold change between the two timepoints with 95% CIs are reported, with no adjustment for multiplicity. Absolute levels of immune responses and fold changes before (day 0) versus 14 days after the fourth dose are summarised by geometric means and 95% CIs. The immunogenicity analyses were also repeated in the seropositive modified intention-to-treat population, which comprised participants who received a fourth-dose booster and who had evidence of SARS-CoV-2 infection before the fourth dose (defined by the Roche Elecsys anti-SARS-CoV-2 nucleocapsid assay or via self-reported PCR or lateral flow test) or within 7 days of the fourth dose (self-reported PCR or lateral flow test).

All analyses were done by use of R, version 4.1.1. This trial is registered with ISRCTN, 73765130. An independent data safety monitoring board reviewed safety data regularly, and local trial site physicians provided oversight of all adverse events in real time.

### Role of the funding source

The funders of the study had no role in study design, data collection, data analysis, data interpretation, or writing of the report.

## Results

**Figure 1:**
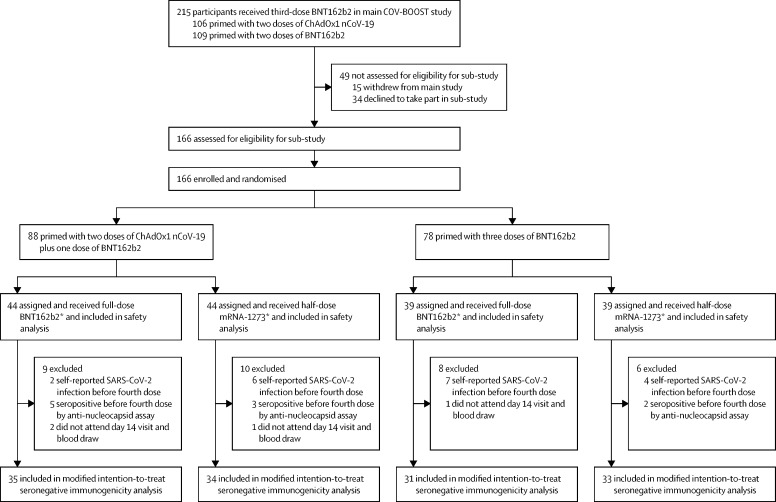
Trial profile *The full dose of BNT162b2 was 30 μg and the half dose of mRNA-1273 was 50 μg.

Among 215 participants who received a third dose of BNT162b2 in June, 2021, 166 people volunteered and were screened from Jan 11 to Jan 25, 2022, for the fourth dose sub-study (figure 1). All participants were eligible and were randomly assigned to receive either full-dose BNT162b2 (n=83) or half-dose mRNA-1273 (n=83) as a fourth-dose vaccine (figure 1). 88 participants had previously received two doses of ChAdOx1 nCoV-19 plus a third dose of BNT162b2 and 78 participants had previously received three doses of BNT162b2 (figure 1, Table 1, Table 2). The median age of the entire cohort was 70·1 years (IQR 51·6–77·5). Among those who had received two doses of ChAdOx1 nCoV-19 plus a third dose of BNT162b2, the baseline characteristics were well balanced between the two fourth-dose groups (table 1). For participants who had received three doses of BNT162b2, those in the fourth-dose BNT162b2 group were younger (median age 67·2 years *vs* 73·2 years) and had a shorter interval between the second and third doses (median time 96·0 days *vs* 106·0 days) than those in the fourth-dose mRNA-1273 group (table 1). The median interval between the third and fourth doses was similar for the four groups and was 208·5 days (IQR 203·3–214·8) for the entire cohort (table 1). 166 participants received a fourth-dose vaccination and were included in the safety and reactogenicity analysis. We excluded 29 participants from the main immunogenicity analysis who were seropositive or had self-reported COVID-19 before the fourth dose and four who did not attend the day 14 visit after the fourth dose. 133 people were included in the modified intention-to-treat immunogenicity analysis of the seronegative population, of whom 66 received full-dose BNT162b2 and 67 received half-dose mRNA-1273 (figure 1; table 2). Where the population does not total 133, there are missing data, the reason for which is still being investigated.

**Table 1:** Baseline characteristics by initial vaccine schedules and sub-study groups in participants who were randomly assigned

	**Two doses of ChAdOx1 nCov-19 plus one dose of BNT162b2**		**Three doses of BNT162b2**	
	Full BNT162b2 as fourth dose (n=44)	Half mRNA-1273 as fourth dose (n=44)	Full BNT162b2 as fourth dose (n=39)	Half mRNA-1273 as fourth dose (n=39)
**Age**				
Median, years	71·2 (52·3–77·4)	71·6 (52·3–77·2)	67·2 (52·9–77·9)	73·2 (52·4–80·5)
<70 years	21 (48%)	21 (48%)	20 (51%)	18 (46%)
≥70 years	23 (52%)	23 (52%)	19 (49%)	21 (54%)
**Intervals between doses, days**				
Between first and second	71·0 (65·0–77·0)	75·5 (67·5–78·0)	69·0 (60·5–76·0)	55·0 (25·5–74·5)
Between second and third	78·0 (73·8–84·2)	76·5 (73·0–85·0)	96·0 (90·0–110·5)	106·0 (90·5–152·5)
Between third and fourth	204·0 (203·0–210·0)	206·0 (204·0–212·2)	208·0 (203·5–214·0)	215·0 (206·5–218·0)
**Sex**				
Female	20 (45%)	22 (50%)	22 (56%)	22 (56%)
Male	24 (55%)	22 (50%)	17 (44%)	17 (44%)
**Occupation**				
Health worker	13 (30%)	11 (25%)	18 (46%)	17 (44%)
Other	31 (70%)	33 (75%)	21 (54%)	22 (56%)
**Diabetes**				
Yes	3 (7%)	4 (9%)	2 (5%)	3 (8%)
No	41 (93%)	40 (91%)	37 (95%)	36 (92%)
**Respiratory disease**				
Yes	2 (5%)	6 (14%)	2 (5%)	3 (8%)
No	42 (95%)	38 (86%)	37 (95%)	36 (92%)
**Cardiovascular disease**				
Yes	20 (45%)	14 (32%)	10 (26%)	9 (23%)
No	24 (55%)	30 (68%)	29 (74%)	30 (77%)
**Ethnicity**				
White	43 (98%)	42 (95%)	36 (92%)	39 (100%)
Black	0	0	0	0
Asian	1 (2%)	2 (5%)	2 (5%)	0
Mixed	0	0	0	0
Other	0	0	1 (3%)	0
Not given	0	0	0	0

Data are median (IQR) or n (%).

**Table 2:** Immune responses in seronegative participants

	**Total (n=133)**		**Two doses of ChAdOx1 nCoV-19 plus one dose of BNT162b2 (n=69)**		**Three doses of BNT162b2 (n=64)**	
	Full-dose BNT162b2 (n=66)	Half-dose mRNA-1273 (n=67)	Full-dose BNT162b2 (n=35)	Half-dose mRNA-1273 (n=34)	Full-dose BNT162b2 (n=31)	Half-dose mRNA-1273 (n=33)
**SARS-CoV-2 anti-spike protein IgG concentration, ELU/mL**						
Day 28 after the third dose	23 325 (20 030–27 162); 66	25 317 (20 996–30 528); 66	20 502 (16 473–25 517); 35	21 980 (16 476–29 324); 33	26 982 (22 056–33 008); 31	29 161 (23 093–36 823); 33
Day 0 of fourth dose	3049 (2550–3646); 66	3469 (2730–4407); 66	2532 (1974–3247); 35	2571 (1874–3527); 34	3761 (2959–4780); 31	4769 (3421–6648); 32
Day 14 after the fourth dose	37 460 (31 996–43 857); 65	54 936 (46 826–64 452); 67	33 316 (26 942–41 198); 35	52 080 (41 163–65 894); 34	42 949 (34 148–54 019); 30	58 043 (46 693–72 150); 33
Fold change (day 14 after fourth dose *vs* day 28 after third dose)	1·59 (1·41–1·78); 65	2·19 (1·90–2·52); 66	1·62 (1·35–1·95); 35	2·41 (1·90–3·05); 33	1·54 (1·35–1·76); 30	1·99 (1·71–2·31); 33
Fold change (day 14 after fourth dose *vs* day 0 of fourth dose)	12·19 (10·37–14·32); 65	15·90 (12·92–19·58); 66	13·16 (10·24–16·91); 35	20·26 (15·09–27·21); 34	11·14 (9·21–13·47); 30	12·30 (9·39–16·11); 32
Cellular response (wild-type), spot forming cells per 10^6^ PBMCs*						
Day 28 after the third dose	96·03 (65·68–140·42; 35	111·19 (75·87–162·95); 33	133·33 (81·31–218·62); 19	113·40 (57·93–221·98); 17	65·04 (37·76–112·03); 16	108·90 (75·81–156·43); 16
Day 0 of fourth dose	19·32 (10·99–33·97);36	35·32 (20·66–60·40); 34	18·85 (8·31–42·77); 20	42·13 (18·58–95·51); 16	19·93 (9·14–43·48); 16	30·20 (14·71–62·02); 18
Day 14 after the fourth dose	112·64 (80·61–157·38); 20	236·95 (146·04–384·48); 20	141·99 (92·57–217·80); 11	232·98 (116·70–465·12); 11	84·87 (51·94–138·66); 9	241·91 (118·79–492·64); 9
Fold change (day 14 after fourth dose *vs* day 28 after third dose)	1·10 (0·72–1·70); 18	1·69 (1·22–2·34); 19	1·09 (0·63–1·89); 10	1·16 (0·79–1·70); 11	1·12 (0·54–2·31); 8	2·83 (2·02–3·96); 8
Fold change (day 14 after fourth dose *vs* day 0 of fourth dose)	7·32 (3·24–16·54); 19	6·22 (3·90–9·92); 20	11·07 (4·21–29·12); 11	6·34 (2·89–13·92); 11	4·14 (1·04–16·54); 8	6·08 (3·86–9·56); 9
Cellular response (beta), spot forming cells per 10^6^ PBMCs*						
Day 28 after the third dose	98·34 (72·11–134·10); 35	108·85 (76·90–154·07); 33	132·70 (87·79–200·59); 19	120·52 (65·57–221·54); 17	68·89 (45·39–104·57); 16	97·69 (70·62–135·12); 16
Day 0 of fourth dose	18·53 (10·60–32·37); 36	28·35 (15·40–52·19); 34	16·71 (7·50–37·22); 20	37·69 (16·24–87·46); 16	21·07 (9·64–46·06); n=16	22·01 (9·11–53·20); 18
Day 14 after the fourth dose	85·55 (54·11–135·28); 20	245·84 (158·84–380·50); 20	96·25 (55·85–165·86); 11	256·42 (142·42–461·65); 11	74·08 (33·34–164·60); 9	233·50 (117·10–465·64); 9
Fold change (day 14 after fourth dose *vs* day 28 after third dose)	0·98 (0·64–1·50); 18	1·96 (1·36–2·82); 19	0·93 (0·54–1·61); 10	1·30 (0·91–1·87); 11	1·03 (0·50–2·12); 8	3·45 (2·10–5·69); 8
Fold change (day 14 after fourth dose *vs* day 0 of fourth dose)	5·47 (2·30–13·02); 19	8·48 (4·72–15·22); 20	7·63 (2·51–23·17); 11	8·98 (4·19–19·23); 11	3·46 (0·85–14·08); 8	7·91 (3·04–20·56); 9
Cellular response (delta), spot forming cells per 10^6^ PBMCs*						
Day 28 after the third dose	92·48 (66·90–127·85); 35	104·34 (72·95–149·24); 33	130·31 (92·00–184·58); 19	114·49 (61·51–213·11); 17	61·55 (36·64–103·38); 16	94·54 (66·74–133·91); 16
Day 0 of fourth dose	16·40 (9·38–28·68); 36	35·93 (20·29–63·62); 34	18·39 (8·68–38·95); 20	41·93 (17·99–97·78); 16	14·22 (6·02–33·58); 16	31·31 (14·19–69·08); 18
Day 14 after the fourth dose	94·20 (66·92–132·60); 20	239·62 (155·19–369·97); 20	108·24 (69·20–169·28); 11	244·67 (141·79–422·20); 11	79·49 (46·55–135·72); 9	233·59 (111·99–487·24); 9
Fold change (day 14 after fourth dose *vs* day 28 after third dose)	1·00 (0·68–1·49); 18	2·03 (1·39–2·97); 19	0·88 (0·56–1·38); 10	1·28 (0·89–1·84); 11	1·19 (0·59–2·40); 8	3·85 (2·39–6·21); 8
Fold change (day 14 after fourth dose *vs* day 0 of fourth dose)	7·57 (3·32–17·25); 19	6·35 (3·51–11·49); 20	9·37 (3·56–24·67); 11	6·62 (2·87–15·25); 11	5·64 (1·27–25·06); 8	6·04 (2·48–14·68); 9

Data are geometric mean (95% CI); number of participants contributing to analysis. ELU=ELISA laboratory units. PBMCs=peripheral blood mononuclear cells.

* Due to logistical reasons, only 50% of study sites collected cellular immunology samples (proximity to external laboratory) in the main COV-BOOST study; the cellular immunology samples after the fourth dose were collected in the immunology cohort.

**Figure 2:**
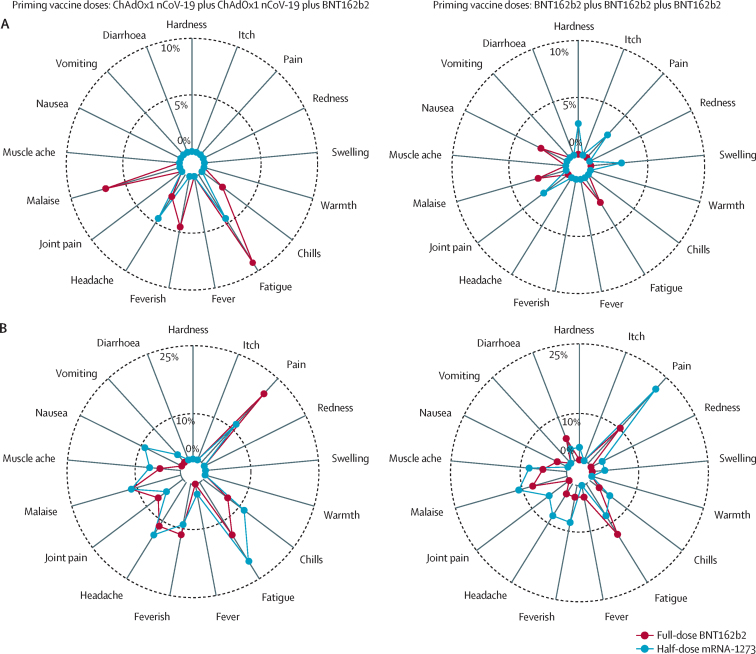
Solicited adverse events within 7 days following fourth-dose vaccination in participants who received a study vaccine (A) Severe (grade 3–4) local and systemic adverse events. (B) Moderate or severe (grade 2–4) local and systemic adverse events. For each solicited adverse event, the highest severity within the first 7 days after fourth-dose vaccination at an individual level was used to draw the plot.

Pain was the most common solicited local adverse event for participants receiving full-dose BNT162b2 and those receiving half-dose mRNA-1273 booster doses and was mostly mild or moderate in severity (appendix 1 pp 2–3). Fatigue, headache, malaise, and muscle ache were the most common solicited systemic adverse events (appendix 1 pp 2–3). One (3%) of 39 participants who received four doses of BNT162b2, two (3%) of 39 participants who received three doses of BNT162b2 and one half-dose of mRNA-1273, four (9%) of 44 people who received two doses of ChAdOx1 nCoV-19 and two doses of BNT162b2, and three (7%) of 44 people who received two doses of ChAdOx1 nCoV-19, one dose of BNT162b2, and one half-dose of mRNA-1273 had any severe (grades 3–4) local and systemic solicited adverse event within 7 days of the fourth dose (figure 2).

Up to the data extraction cutoff date of March 2, 2022, three serious adverse events, all in recipients of BNT162b2 as a fourth dose, were reported, none of which were related to the study vaccine (appendix 1 pp 9–11). 16 adverse events were reported after fourth-dose BNT162b2 and 18 adverse events were reported after fourth-dose mRNA-1273 (including unsolicited adverse events within 28 days, medically attended adverse events within 3 months, and all other adverse events reported up to data lock). Four adverse events of special interest were reported in the group who received three doses of BNT162b2 and one half-dose of mRNA-1273, all of which were unrelated to the study vaccine (appendix 1 pp 9–11).

**Figure 3:**
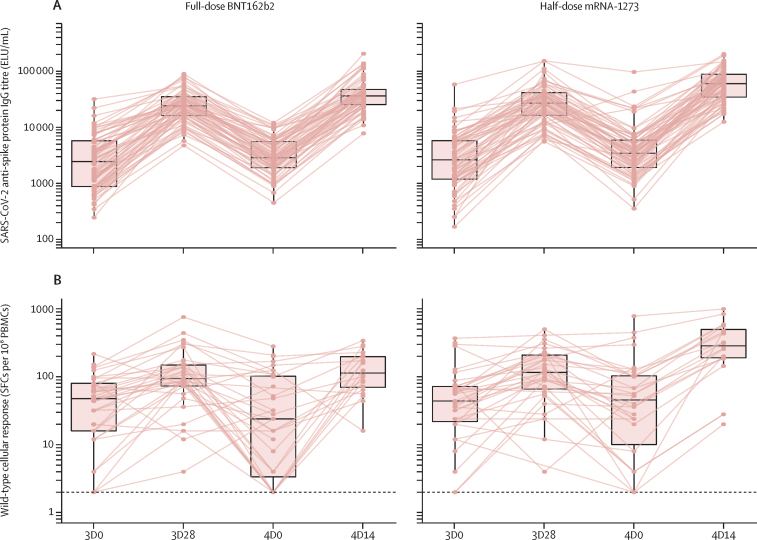
Kinetics of immunogenicity in seronegative participants (A) Anti-spike protein IgG titres. (B) Cellular response. Boxplots represent the median and 25th and 75th percentiles. Each data point is one participant. Solid lines connect samples from the same participant at multiple timepoints. The dashed line represents the lower limit of detection by the ELISpot assay. 3D0=pre-third dose. 3D28=28 days after the third dose. 4D0=pre-fourth dose. 4D14=14 days after the fourth dose. ELU=ELISA laboratory units. PBMCs=peripheral blood mononuclear cells. SFCs=spot forming cells.

In the group who received BNT162b2 as their fourth dose, geometric mean anti-spike protein IgG concentration at day 28 after the third dose was 23 325 ELISA laboratory units (ELU)/mL (95% CI 20 030–27 162), which increased to 37 460 ELU/mL (31 996–43 857) after the fourth dose, representing a significant fold change (geometric mean 1·59, 95% CI 1·41–1·78; table 2; figure 3). Similarly, there was a significant increase in geometric mean anti-spike protein IgG concentration from 28 days after the third dose (25 317 ELU/mL, 95% CI 20 996–30 528) to 14 days after a fourth dose of mRNA-1273 (54 936 ELU/mL, 46 826–64 452), with a geometric mean fold change of 2·19 (1·90–2·52; table 2; figure 3). This increase in anti-spike protein IgG titres between these two timepoints was observed regardless of initial vaccine schedule or age group (Table 2, Table 3; appendix 1 pp 4–5). There was a considerable decay of anti-spike protein IgG titres during approximately 7 months from day 28 after the third dose to just before the fourth dose (day 0; figure 3), leading to a geometric mean fold change between day 0 and day 14 of the fourth dose ranging from 11·14 to 20·26 (table 2).

**Table 3:** Anti-spike protein IgG titres by vaccine schedule and age group in seronegative participants

	**SARS-CoV-2 anti-spike protein IgG concentration, ELU/mL**			**Fold change**		
	Day 0 of third dose	Day 28 after third dose	Day 0 of fourth dose	Day 14 after fourth dose	Day 14 after fourth dose *vs* day 28 after third dose	Day 14 after fourth dose *vs* day 0 of fourth dose
**Three doses of BNT162b2 plus BNT162B2**						
Age <70 years	5421 (4855–6054); 13	24 055 (22 180–26 088); 13	3895 (3486–4351); 13	35 116 (31 868–38 696); 12	1·37 (1·31–1·42); 12	8·45 (7·83–9·11); 12
Age ≥70 years	4047 (3551–4612); 18	29 314 (27 468–31 285); 18	3667 (3413–3940); 18	49 120 (45 756–52 730); 18	1·68 (1·60–1·75); 18	13·39 (12·68–14·15); 18
**Three doses of BNT162b2 plus mRNA-1273**						
Age <70 years	4449 (4027–4916); 15	24 040 (22 444–25 748); 15	3203 (2971–3452); 15	46 053 (42 311–50 126); 15	1·92 (1·80–2·04); 15	14·38 (13·00–15·91); 15
Age ≥70 years	4812 (4255–5441); 18	34 253 (31 499–37 247); 18	6778 (5970–7695); 17	70 387 (66 103–74 947); 18	2·05 (1·97–2·15); 18	10·71 (9·79–11·72); 17
**Two doses of ChAdOx1 nCoV-19 plus BNT162B2 plus BNT162B2**						
Age <70 years	1496 (1345–1663); 16	23 299 (21 376–25 395); 16	2630 (2415–2865); 16	34 582 (32 335–36 985); 16	1·48 (1·39–1·58); 16	13·15 (12·09–14·29); 16
Age ≥70 years	1154 (1067–1248); 19	18 409 (17 268–19 626); 19	2451 (2254–2665); 19	32 286 (29 965–34 787); 19	1·75 (1·65–1·86); 19	13·17 (12·09–14·35); 19
**Two doses of ChAdOx1 nCoV-19 plus BNT162B2 plus mRNA-1273**						
Age <70 years	1416 (1240–1616); 14	21 607 (19 457–23 996); 14	2391 (2130–2683); 15	47 167 (43 536–51 102); 15	2·25 (2·04–2·48); 14	19·73 (17·59–22·13); 15
Age ≥70 years	1249 (1115–1400); 19	22 259 (20 225–24 499); 19	2722 (2454–3019); 19	56 318 (52 024–60 966); 19	2·53 (2·36–2·72); 19	20·69 (18·86–22·7); 19

Data are geometric mean (95% CI); number of participants contributing to analysis. ELU=ELISA laboratory units.

Among participants with cellular response data available, similar T-cell responses were seen at day 14 after the fourth dose compared with day 28 after the third dose across tested variants for participants who received two doses of ChAdOx1 nCoV-19 plus two doses of BNT162b2, two doses of ChAdOx1 nCoV-19 plus one dose of BNT162b2 plus one half dose of mRNA-1273, or four doses of BNT162b2 (table 2; figure 3; appendix p 7). However, among participants who received three doses of BNT162b2 and one half-dose of mRNA-1273, T-cell responses were significantly increased 14 days after the fourth dose compared with 28 days after the third dose (table 2; figure 3). Similar to anti-spike protein IgG titres, a decay of cellular response was also seen from 28 days after the third dose to day 0 of the fourth dose (figure 3), resulting in a significant boosting effect on cellular response in most groups after the fourth dose (fold change ranging from 3·46 to 11·07; table 2).

For participants with evidence of SARS-CoV-2 infection before or within 7 days of the fourth dose, there were 4·89-fold (95% CI 4·35–5·50; n=13) and 4·63-fold (4·04–5·29; n=15) increases in anti-spike protein IgG titres from day 0 of the fourth dose to day 14 after the fourth dose for full-dose BNT162b2 and half-dose mRNA-1273, respectively (appendix 1 p 8). A boost effect on T-cell responses in this population was also seen between day 0 and day 14 relative to the fourth dose, although the sample size was small (appendix 1 p 8).

## Discussion

To our knowledge, we present the first data from a randomised trial on the safety, reactogenicity, and immunogenicity of full-dose BNT162b2 and half-dose mRNA-1273 COVID-19 vaccines given as fourth-dose boosters in healthy adult populations who had previously received different vaccine schedules. These data show that a fourth dose of COVID-19 mRNA vaccines is well tolerated and can provide a substantial boost to both humoral and cellular immunity approximately 7 months after a third-dose booster, with anti-spike protein IgG titres at day 14 following the fourth dose higher than those at day 28 after the third dose for both BNT162b2 and mRNA-1273.

The peak anti-spike protein IgG concentration after a fourth vaccine dose was also higher than after a third dose for full-dose BNT162b2 and half-dose mRNA-1273 among participants in an Israeli observational study who had previously received three doses of BNT162b2 and had low anti-spike IgG titres before the fourth dose.^6^ The fold changes before and after the fourth dose in the Israeli study were lower to those found in our study, probably due to the shorter interval between third and fourth doses in the Israeli study as a longer duration between vaccine doses is recognised to increase immunogenicity.^10^, ^11^ A large increase from baseline in neutralising antibody titres after a fourth dose of mRNA COVID-19 vaccine was also observed in a German observational study, although neutralising capacity against omicron subvariants remained low.^12^

In our study, the fold change in anti-spike protein IgG titres between day 0 and day 14 of the fourth dose ranged from 11·14 to 20·26. There are two possible explanations for such a large fold change: first, the vaccines remain strongly immunogenic, and, second, the boost is from a relatively low baseline following waning of immunity after the third dose. Baseline anti-spike protein IgG concentrations before the fourth dose (day 0) were similar to baseline concentrations before the third dose (day 0). Some participants in our study maintained high levels of humoral and cellular responses even before the fourth dose and had limited boosting from the fourth dose. This finding was replicated in participants with a history of SARS-CoV-2 infection, indicating that there might be a ceiling or maximum anti-spike protein IgG titre and T-cell response and that the fourth dose might not boost humoral and cellular responses if the baseline response is high. These individual data are important for policy makers as the benefit of a fourth dose might be less in people who already have high levels of immune responses from recent infection or vaccination. In addition, this ceiling effect could be dependent on vaccine type and dose. If this ceiling effect is replicated in other datasets, it could be due to host immunity, vaccine type, or vaccine dose, which needs to be explored in further trials and analyses.

Our results for immunogenicity are also consistent with the little observational evidence on vaccine effectiveness available from Israel, which indicates increased protection against symptomatic infection and severe illness from a fourth-dose booster.^6^, ^7^ In our study, half-dose mRNA-1273 appeared to have higher immunogenicity than full-dose BNT162b2, which was also seen in the Israeli study,^6^ although the two groups in the Israeli study were not randomised. This result might be due to a heterologous schedule effect or the vaccine dose. For third doses given in the main COV-BOOST study, heterologous mRNA vaccines appeared to provide a superior boost to third homologous doses.^1^, ^13^ In addition to the boost to humoral immunity, there was also a boost in broad cellular responses after a fourth vaccine dose. Due to the small number of samples available for analysis, it is difficult to quantify the size of the booster effect or make direct comparisons across all the schedules tested. A higher number of samples will be tested at the day 84 timepoint to investigate any differences.

Our study has several limitations. The number of participants within each subgroup is relatively small as we recruited only existing COV-BOOST participants who had received BNT162b2 as their third dose within the study. An even smaller number of samples were available for our analysis of cellular immunity, meaning low levels of precision to quantify T-cell responses. There were not enough samples to investigate any potential benefit of heterologous schedules on cellular responses. The timepoints after the third and fourth doses were different, but humoral responses in previous studies were at similar levels between day 7 and day 28 after vaccination.^1^, ^6^ Due to laboratory capacity, data for neutralising antibodies against variants of concern, including omicron, were not available when this Article was developed. Given that a strong correlation has been observed between anti-spike protein IgG titres and neutralising antibody titres against SARS-CoV-2 variants of concern,^1^ it is expected that the titres of neutralising antibodies after a fourth dose are similar to those observed following a third dose. Furthermore, only mRNA vaccines, which are currently difficult to obtain or are unavailable in many low-income or middle-income countries, were analysed as fourth-dose vaccines in this study.

The strengths of this study include it being the first to report on mixed-schedule fourth-dose data from a randomised trial and on populations who had received vaccines other than BNT162b2 as their first, second, or third dose. This study provides important data to help guide policy makers in decisions on how to use fourth doses of COVID-19 vaccines.

## Data sharing

The study protocol is provided as appendix 2 and the statistical analysis plan is provided as appendix 3. Individual participant data will be made available when the study is complete upon reasonable requests made to the corresponding author (s.faust@soton.ac.uk); data can be shared through secure online platforms after proposals are approved. All the sequence datasets used in the T-cell analysis are available in the public GISAID database (https://www.gisaid.org).

## Declaration of interests

KCa acts on behalf of University Hospital Southampton as an investigator on studies funded or sponsored by vaccine manufacturers, including AstraZeneca, GlaxoSmithKline, Janssen, Medimmune, Merck, Pfizer, Sanofi, and Valneva, and receives no personal financial payment for this work. SNF acts on behalf of University Hospital Southampton NHS Foundation Trust as an investigator or consults on clinical trials and studies of COVID-19 vaccines and other vaccines funded or sponsored by vaccine manufacturers, including Janssen, Pfizer, AstraZeneca, GlaxoSmithKline, Novavax, Seqirus, Sanofi, Medimmune, Merck, and Valneva, and receives no personal financial payment for this work. ALG is named as an inventor on a patent covering the use of a particular promoter construct that is often used in ChAdOx1-vectored vaccines and is incorporated in the ChAdOx1 nCoV-19 vaccine and could benefit from royalty income paid to the University of Oxford from sales of this vaccine by AstraZeneca and its sublicensees under the university's revenue sharing policy. JH has received payments for presentations for AstraZeneca, Boehringer Ingelheim, Chiesi, and Cipla & Teva. VL acts on behalf of University College London Hospitals NHS Foundation Trust as an investigator on clinical trials of COVID-19 vaccines funded or sponsored by vaccine manufacturers, including Pfizer, AstraZeneca, and Valneva, and receives no personal financial payment for this work. PM acts on behalf of University Hospital Southampton NHS Foundation Trust and The Adam Practice as an investigator on studies funded or sponsored by vaccine manufacturers, including AstraZeneca, GlaxoSmithKline, Novavax, Medicago, and Sanofi, and receives no personal financial payment for this work. JSN-V-T was seconded to the Department of Health and Social Care, England, until March 31, 2022. MR has provided post-marketing surveillance reports on vaccines for Pfizer and GlaxoSmithKline, for which a cost recovery charge is made. MDS acts on behalf of the University of Oxford as an investigator on studies funded or sponsored by vaccine manufacturers, including AstraZeneca, GlaxoSmithKline, Pfizer, Novavax, Janssen, Medimmune, and MCM, and has received no personal financial payment for this work. All other authors declare no competing interests.
