# Supplementary material for: Safety, immunogenicity, and reactogenicity of BNT162b2 and mRNA-1273 COVID-19 vaccines given as fourth-dose boosters following two doses of ChAdOx1 nCoV-19 or BNT162b2 and a third dose of BNT162b2 (COV-BOOST): a multicentre, blinded, phase 2, randomised trial
Source: Lancet Infect Dis. 2022 Aug;22(8):1131–41. doi: 10.1016/S1473-3099(22)00271-7 (PMC9084623; doi:10.1016/S1473-3099(22)00271-7)
Supplement: Supplementary appendix 1 [file mmc1.pdf]

THE LANCET
Infectious Diseases

Supplementary appendix 1

This appendix formed part of the original submission and has been peer reviewed. We post it as supplied by the authors.

Supplement to: Munro APS, Feng S, Janani L, et al. Safety, immunogenicity, and reactogenicity of BNT162b2 and mRNA-1273 COVID-19 vaccines given as fourth-dose boosters following two doses of ChAdOx1 nCoV-19 or BNT162b2 and a third dose of BNT162b2 (COV-BOOST): a multicentre, blinded, phase 2, randomised trial. *Lancet Infect Dis* 2022; published online May 9. https://doi.org/10.1016/S1473-3099(22)00271-7.

Immunogenicity and reactogenicity of BNT162b2 and mRNA1273 COVID-19 vaccines given as fourth dose boosters in the COV-BOOST randomised trial following two doses of ChAdOx1 nCov-19 or BNT162b2 and a third dose of BNT162b2

Supplementary Appendix

Contents

Supplementary Figure 1. Reactogenicity of fourth dose after ChAd/ChAd/BNT in participants who received a study vaccine 2

Supplementary Figure 2. Reactogenicity of fourth dose after BNT/BNT/BNT in participants who received a study vaccine 3

Supplementary Figure 3. SARS-CoV-2 anti-spike IgG of D28 at the third dose and D14 of the fourth dose by vaccine schedules in seronegative participants 4

Supplementary Figure 4. Kinetics of immunogenicity post third dose by priming vaccines, fourth dose and age group in seronegative participants 5

Supplementary Figure 5. Kinetics of immunogenicity of anti-spike IgG by vaccine schedules in seronegative participants 6

Supplementary Figure 6. Kinetics of cellular responses A) Beta and B) Delta in seronegative participants 7

Supplementary Figure 7. Kinetics of immunogenicity 8

Supplementary Table 1. Summary of adverse events in all participants who received a study vaccine 9

Supplementary Table 2. Adverse Events of Special Interest in all participants who received a study vaccine 11

Supplementary Table 3. Serious Adverse Events in all participants who received a study vaccine 11

Laboratory Units Conversion and Assays 12

COV-BOOST Study Group 13

Supplementary Figure 1. Reactogenicity of fourth dose after ChAd/ChAd/BNT in participants who received a study vaccine

Local (A) and systemic (B) adverse reactions following the fourth dose vaccination in participants who have previously received ChAd/ChAd/BNT

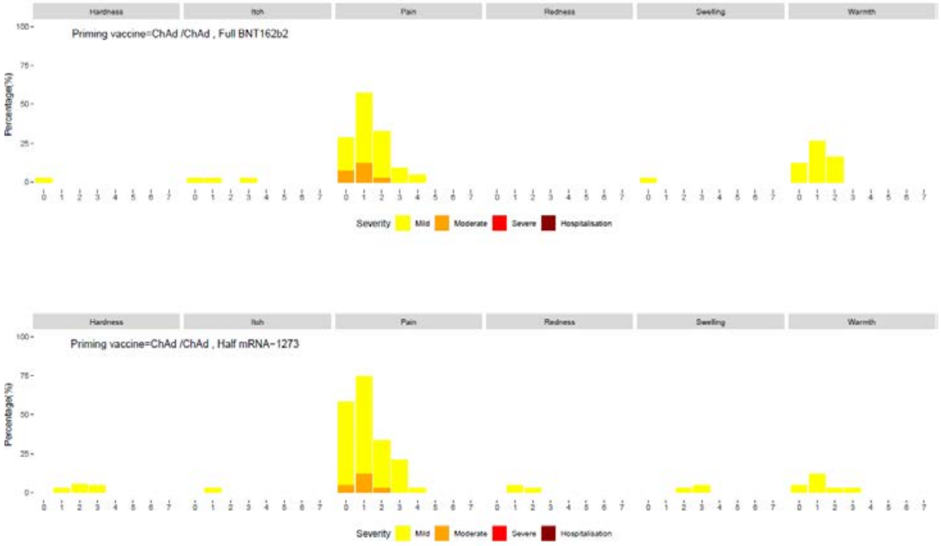

A)

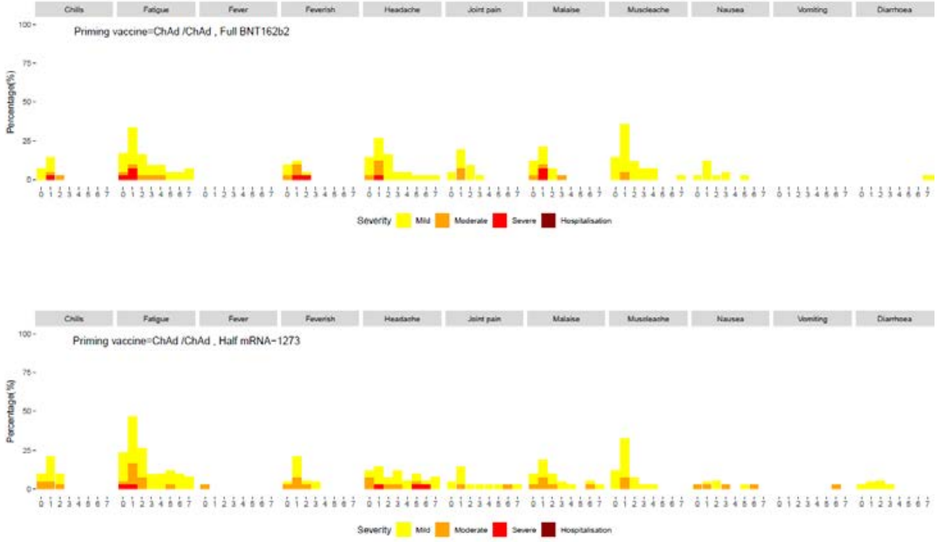

B)

Supplementary Figure 2. Reactogenicity of fourth dose after BNT/BNT/BNT in participants who received a study vaccine

Local (A) and systemic (B) adverse reactions following the fourth dose vaccination in participants who have previously received ChAd/ChAd/BNT

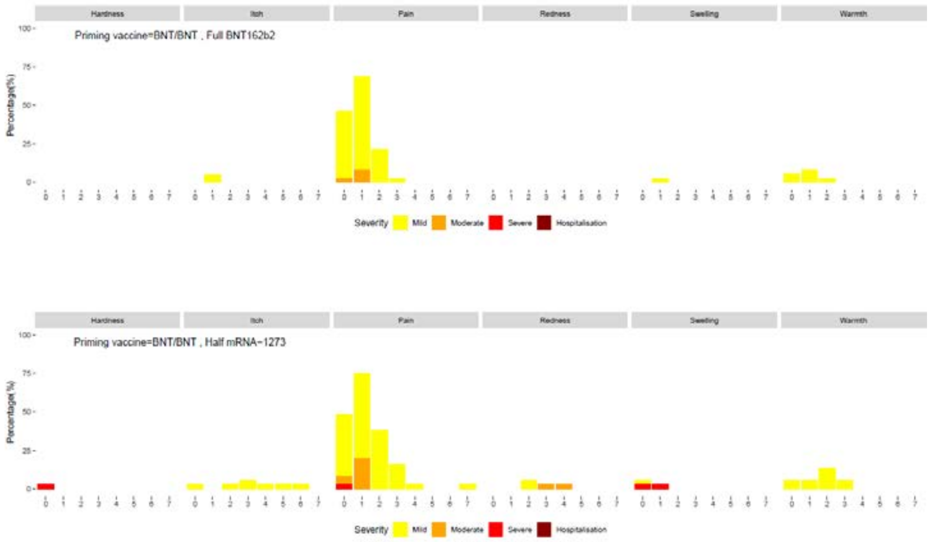

A)

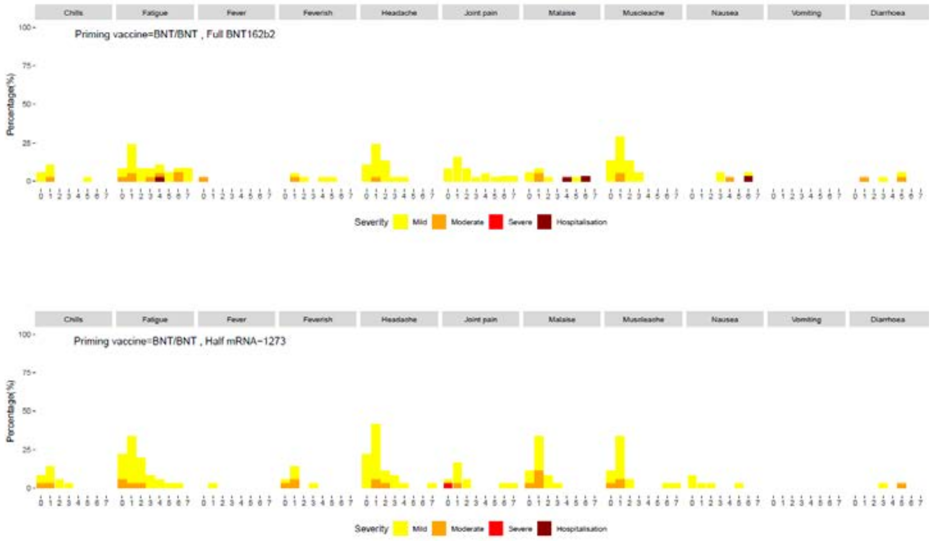

B)

One participant in BNT/BNT/BNT/BNT arm reported Grade4 for Fatigue and Malaise on day 4 and for Malaise and Nausea on day 6. This participant had a small bowel obstruction and was admitted to hospital. Reported as an SAE, unrelated to study vaccine.

Supplementary Figure 3. SARS-CoV-2 anti-spike IgG of D28 at the third dose and D14 of the fourth dose by vaccine schedules in seronegative participants

3D0: pre-third dose; 4D14: 14 days post fourth dose; boxplots represent the median and 25th and 75th percentiles; each data point is one participant; solid lines connect samples from the same participant at multiple time points.

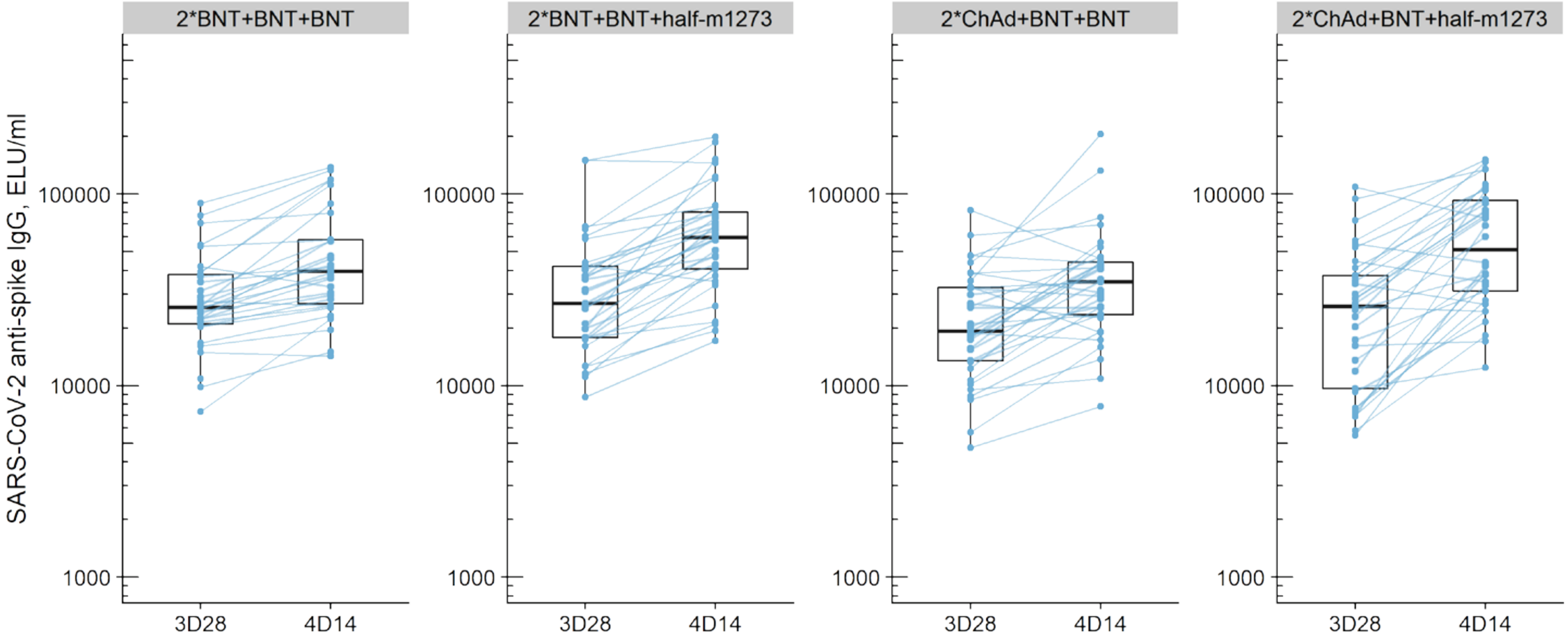

Supplementary Figure 4. Kinetics of immunogenicity post third dose by priming vaccines, fourth dose and age group in seronegative participants

3D0: pre-third dose; 3D28: 28 days post third dose; 4D0: pre-fourth dose; 4D14: 14 days post fourth dose; data points are medians with IQRs.

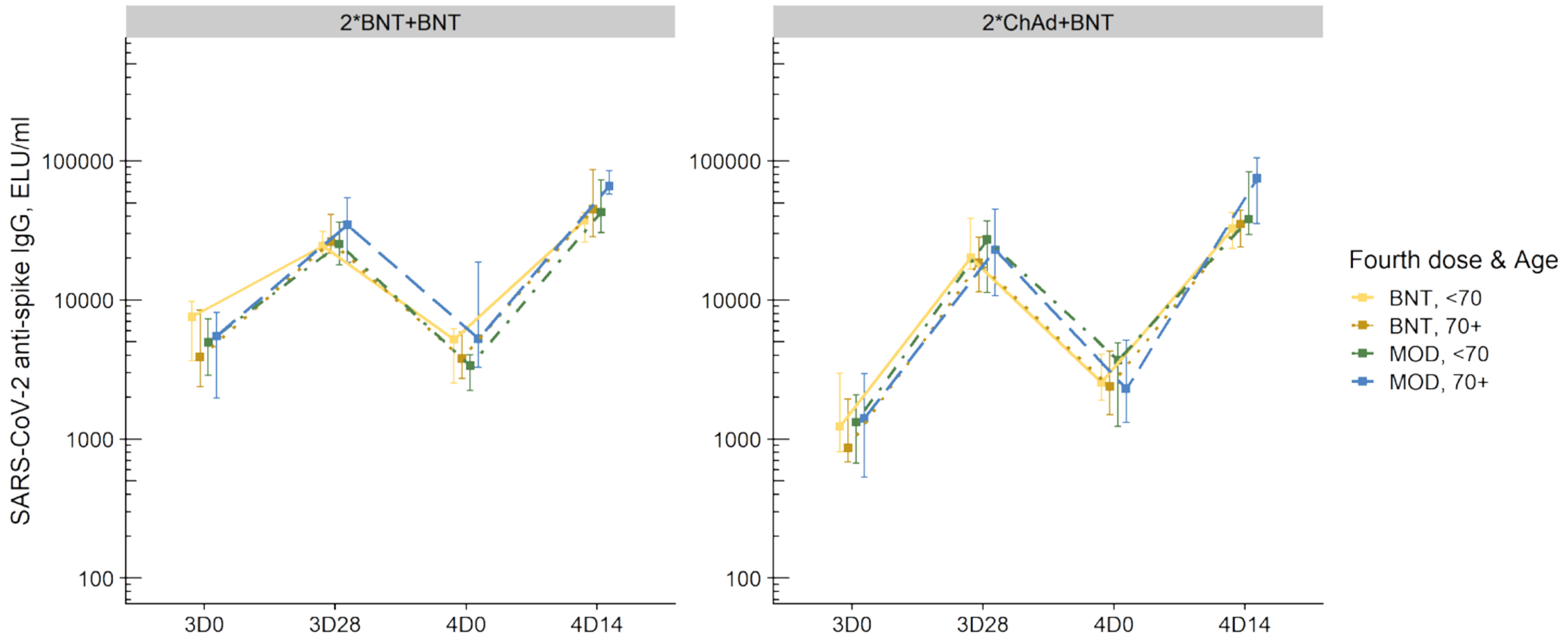

Supplementary Figure 5. Kinetics of immunogenicity of anti-spike IgG by vaccine schedules in seronegative participants

3D0: pre-third dose; 3D28: 28 days post third dose; 4D0: pre-fourth dose; 4D14: 14 days post fourth dose; boxplots represent the median and 25th and 75th percentiles; each data point is one participant; solid lines connect samples from the same participant at multiple time points.

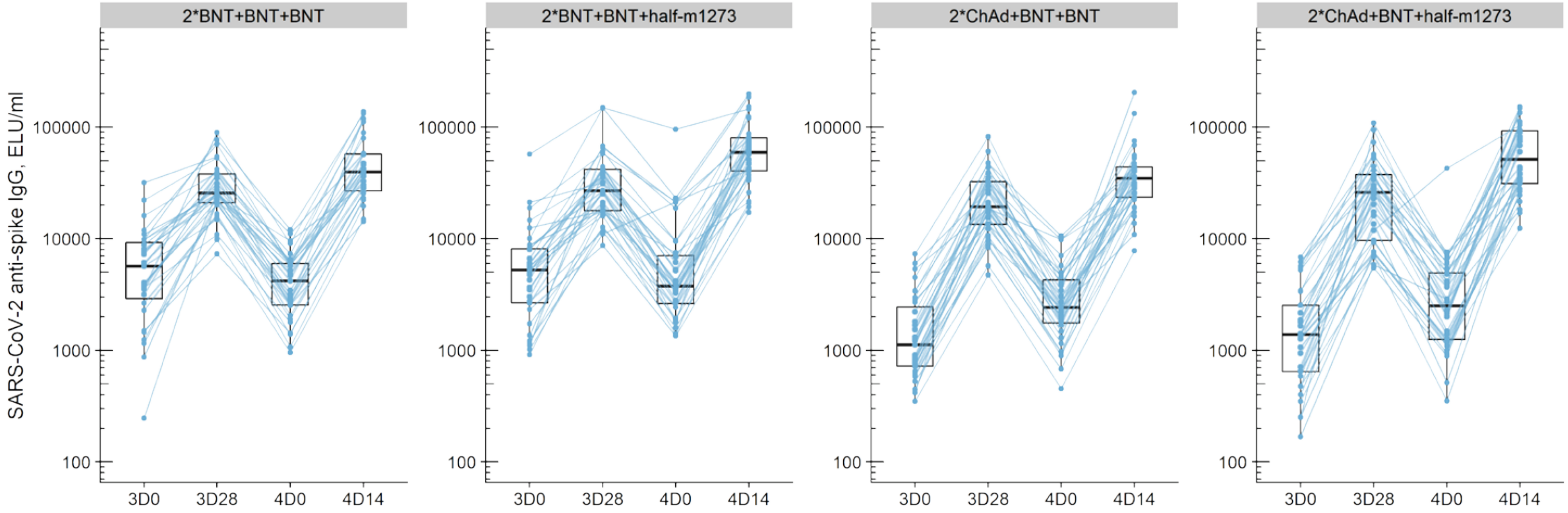

Supplementary Figure 6. Kinetics of cellular responses A) Beta and B) Delta in seronegative participants

3D0: pre-third dose; 3D28: 28 days post third dose; 4D0: pre-fourth dose; 4D14: 14 days post fourth dose; boxplots represent the median and 25th and 75th percentiles; each data point is one participant; solid lines connect samples from the same participant at multiple time points.

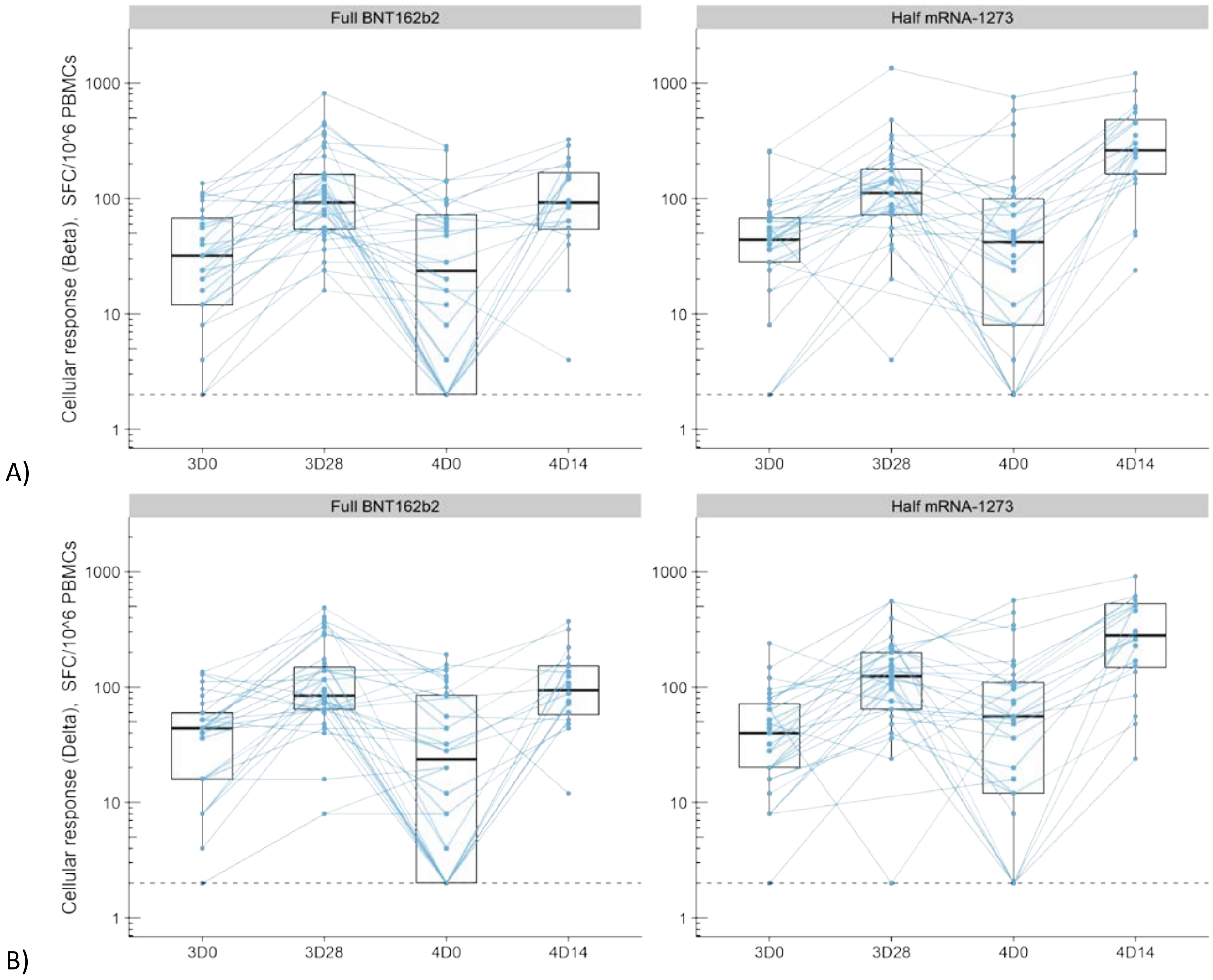

Supplementary Figure 7. Kinetics of immunogenicity
A) anti-spike IgG; B) cellular response post third dose among seropositive or self-reported SARS-CoV-2 positive (PCR/lateral flow test) participants

3D0: pre-third dose; 3D28: 28 days post third dose; 4D0: pre-fourth dose; 4D14: 14 days post fourth dose; boxplots represent the median and 25th and 75th percentiles; each data point is one participant; solid lines connect samples from the same participant at multiple time points.

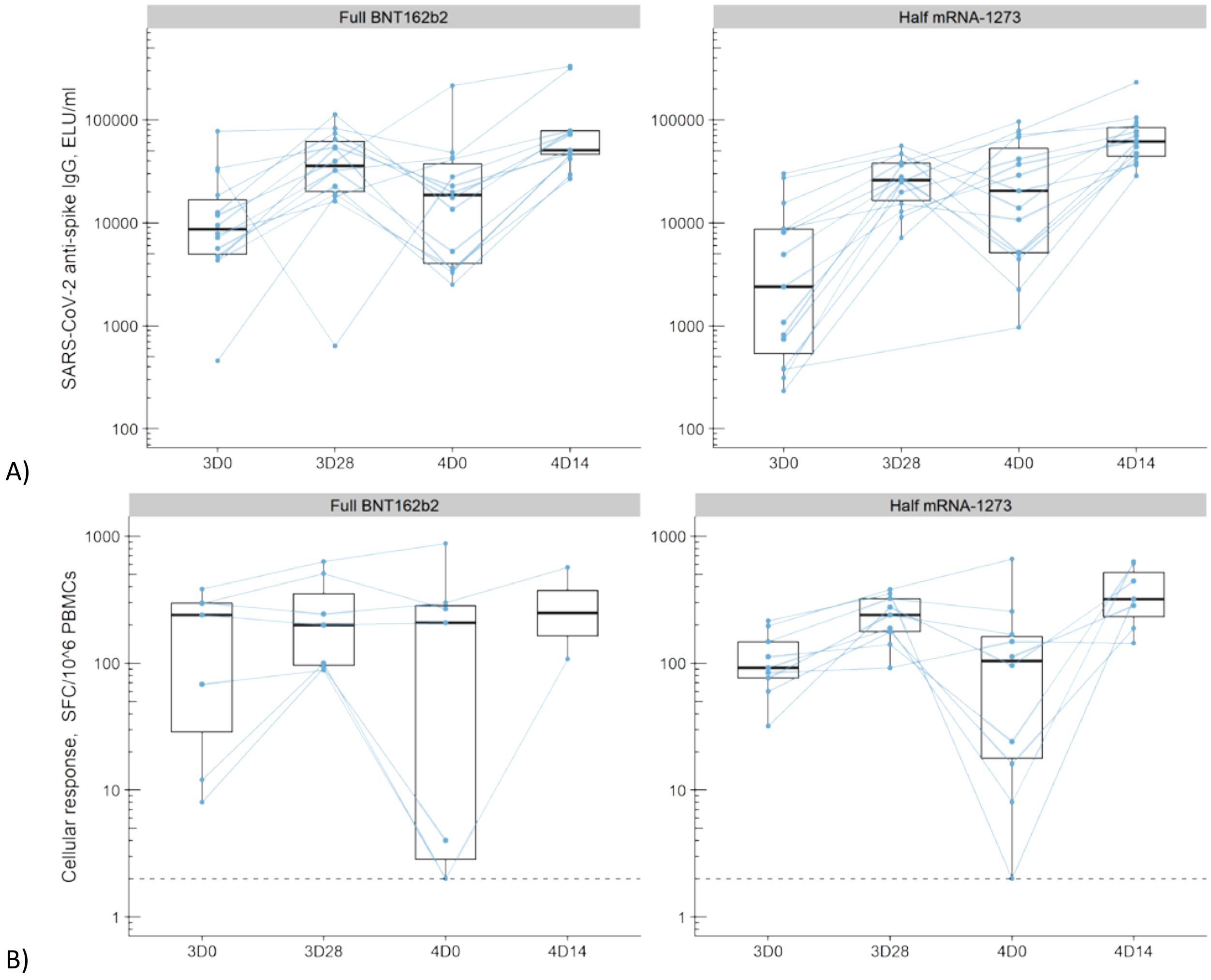

Supplementary Table 1. Summary of adverse events in all participants who received a study vaccine

	Priming vaccine ChAdOx1-nCov19/ ChAdOx1-nCov19		Priming vaccine BNT162b2/ BNT162b2	
**N=Number of vaccinated participants**	**Full BNT162b2 (N=44)**	**Half mRNA-1273 (N=44)**	**Full BNT162b2 (N=39)**	**Half mRNA-1273 (N=39)**
**Number of unique participants with at least one adverse event**	10	10	5	6
**Number of adverse events**	11	12	5	6
**Adverse events with special interest**				
No	11 (100.0%)	12 (100.0%)	5 (100.0%)	2 (33.3%)
Yes	0 (0.0%)	0 (0.0%)	0 (0.0%)	4 (66.7%)
**Serious adverse events**				
No	9 (81.8%)	12 (100.0%)	4 (80.0%)	6 (100.0%)
Yes - hospitalisation	2 (18.2%)	0 (0.0%)	1 (20.0%)	0 (0.0%)
**Severity**				
Grade 1	5 (45.5%)	6 (50.0%)	3 (60.0%)	5 (83.3%)
Grade 2	4 (36.4%)	3 (25.0%)	1 (20.0%)	1 (16.7%)
Grade 3	2 (18.2%)	2 (16.7%)	1 (20.0%)	0 (0.0%)
Grade 4	0 (0.0%)	1 (8.3%)	0 (0.0%)	0 (0.0%)
**Causality**				
No relationship	9 (81.8%)	8 (66.7%)	2 (40.0%)	5 (83.3%)
Unlikely	1 (9.1%)	2 (16.7%)	1 (20.0%)	0 (0.0%)
Possible	0 (0.0%)	0 (0.0%)	1 (20.0%)	0 (0.0%)
Probable	1 (9.1%)	2 (16.7%)	0 (0.0%)	1 (16.7%)
Definite	0 (0.0%)	0 (0.0%)	1 (20.0%)	0 (0.0%)
**System Organ Classes (SOC)**				
Blood and lymphatic system disorders	1 (9.1%)	1 (8.3%)	1 (20.0%)	0 (0.0%)
Cardiac disorders	0 (0.0%)	1 (8.3%)	0 (0.0%)	0 (0.0%)
Congenital, familial and genetic disorders	0 (0.0%)	0 (0.0%)	0 (0.0%)	0 (0.0%)
Ear and labyrinth disorders	0 (0.0%)	1 (8.3%)	0 (0.0%)	0 (0.0%)
Endocrine disorders	0 (0.0%)	0 (0.0%)	0 (0.0%)	0 (0.0%)
Eye disorders	0 (0.0%)	1 (8.3%)	0 (0.0%)	0 (0.0%)
Gastrointestinal disorders	0 (0.0%)	1 (8.3%)	1 (20.0%)	0 (0.0%)
General disorders and administration site conditions	1 (9.1%)	0 (0.0%)	1 (20.0%)	0 (0.0%)
Hepatobiliary disorders	0 (0.0%)	0 (0.0%)	1 (20.0%)	0 (0.0%)
Immune system disorders	0 (0.0%)	0 (0.0%)	0 (0.0%)	1 (16.7%)
Infections and infestations	2 (18.2%)	0 (0.0%)	1 (20.0%)	5 (83.3%)
Injury, poisoning and procedural complications	2 (18.2%)	1 (8.3%)	0 (0.0%)	0 (0.0%)
Investigations	0 (0.0%)	1 (8.3%)	0 (0.0%)	0 (0.0%)

Metabolism and nutrition disorders	0 (0.0%)	0 (0.0%)	0 (0.0%)	0 (0.0%)
Musculoskeletal and connective tissue disorders	3 (27.3%)	2 (16.7%)	0 (0.0%)	0 (0.0%)
Neoplasms benign, malignant and unspecified (incl cysts and polyps)	0 (0.0%)	0 (0.0%)	0 (0.0%)	0 (0.0%)
Nervous system disorders	0 (0.0%)	2 (16.7%)	0 (0.0%)	0 (0.0%)
Pregnancy, puerperium and perinatal conditions	0 (0.0%)	0 (0.0%)	0 (0.0%)	0 (0.0%)
Product issues	0 (0.0%)	0 (0.0%)	0 (0.0%)	0 (0.0%)
Psychiatric disorders	0 (0.0%)	0 (0.0%)	0 (0.0%)	0 (0.0%)
Renal and urinary disorders	1 (9.1%)	0 (0.0%)	0 (0.0%)	0 (0.0%)
Reproductive system and breast disorders	0 (0.0%)	0 (0.0%)	0 (0.0%)	0 (0.0%)
Respiratory, thoracic and mediastinal disorders	0 (0.0%)	0 (0.0%)	0 (0.0%)	0 (0.0%)
Skin and subcutaneous tissue disorders	0 (0.0%)	1 (8.3%)	0 (0.0%)	0 (0.0%)
Social circumstances	0 (0.0%)	0 (0.0%)	0 (0.0%)	0 (0.0%)
Surgical and medical procedures	0 (0.0%)	0 (0.0%)	0 (0.0%)	0 (0.0%)
Vascular disorders	1 (9.1%)	0 (0.0%)	0 (0.0%)	0 (0.0%)
**Menstrual disorder**				
Yes	0 (0.0%)	0 (0.0%)	0 (0.0%)	0 (0.0%)

Data are frequency (percentage) unless otherwise indicated.

Supplementary Table 2. Adverse Events of Special Interest in all participants who received a study vaccine

Study arm	Site	Days to onset from boost	MedDRA Preferred Term	MedDRA System Order Class	Severity	Causality assessment
Half mRNA-1273	University Hospital Southampton NHS FT	0	COVID-19	Infections and infestations	Grade1	No relationship
Half mRNA-1273	University of Oxford	38	COVID-19	Infections and infestations	Grade1	No relationship
Half mRNA-1273	Guys and St Thomas' NHS FT	23	COVID-19	Infections and infestations	Grade1	No relationship
Half mRNA-1273	Cambridge University Hospitals NHS FT	33	COVID-19	Infections and infestations	Grade1	No relationship

Supplementary Table 3. Serious Adverse Events in all participants who received a study vaccine

Study arm	Site	Days to onset from boost	MedDRA Preferred Term	MedDRA System Order Class	Severity	Serious adverse event type	Causality assessment
Full BNT162b2	University Hospital Southampton NHS FT	12	Back pain	Musculoskeletal and connective tissue disorders	Grade3	Hospitalisation	No relationship
Full BNT162b2	University Hospital Southampton NHS FT	9	Renal colic	Renal and urinary disorders	Grade3	Hospitalisation	No relationship
Full BNT162b2	Cambridge University Hospitals NHS FT	4	Small intestinal obstruction	Gastrointestinal disorders	Grade3	Hospitalisation	No relationship

Laboratory Units Conversion and Assays

Anti-spike IgG

Sera were analysed at Nexelis (Laval, QC, Canada) to determine SARS-CoV-2 anti-spike IgG concentrations by ELISA (reported as ELISA laboratory units [ELU]/mL). The conversion factor to international standard units is:

Human SARS-CoV-2 Pre-Spike IgG ELISA Conversion

The results generated for the Human SARS-CoV-2 Pre-Spike IgG ELISA are reported with concentration units in "ELU/mL". When required a correlation factor of 1/7.9815 will be applied to convert the reported results from ELU/mL to BAU/mL. For example, a sample with reported anti-PreSpike IgG antibody concentration of 7981.5 ELU/mL will have a concentration equivalent to 1000 BAU/mL.

The following formula may be used for converting concentration units from ELU/mL to BAU/mL:
Result (BAU/mL) = Result (ELU/mL) / 7.9815.

Anti-nucleocapsid IgG

Sera were analysed at Porton Down, Public Health England, by ECLIA (Cobas platform, Roche Diagnostics) to determine anti-SARS-CoV-2 nucleocapsid IgG status (reported as negative if below a cut-off index (COI) of 1·0).

Cellular immunity

The cellular immunology samples were collected from nine sites based on logistical reasons in the main COV-BOOST trial (i.e. proximity to external laboratory) and in the immunology cohort (n=~25) in the fourth dose sub-study. IFN-γ secreting T cells specific to whole spike protein epitopes designed based on the Wuhan-Hu-1 sequence (YP_009724390.1) were detected by modified TSPOT-Discovery test within 32 hours (h) of venepuncture, using the addition of T-Cell Xtend reagent to extend peripheral blood mononuclear cell (PBMC) survival, at Oxford Immunotec (Abingdon, UK). T-cell frequencies were reported as spot forming cells (SFC) per 250,000 PBMCs with a lower limit of detection of one in 250,000 PBMCs, and these results were multiplied by four to express frequencies per million PBMCs.

COV-BOOST Study Group

Name	Affiliation
Andrew Riordan	Data Monitoring Safety Committee, Alder Hey Children's Hospital
Andrew Ustianowski	Data Monitoring Safety Committee, North Manchester General Hospital
Chris Rogers	Data Safety Monitoring Committee, University of Bristol
Kashyap Katechia	Betsi Cadwaladr University Health Board
Alison Cooper	Betsi Cadwaladr University Health Board
Andrew Freedman	Betsi Cadwaladr University Health Board
Rachel Hughes	Betsi Cadwaladr University Health Board
Lynne Grundy	Betsi Cadwaladr University Health Board
Lona Tudor Jones	Betsi Cadwaladr University Health Board
Elizabeth Harrison	Betsi Cadwaladr University Health Board
Emma Snashall	Betsi Cadwaladr University Health Board
Lewis Mallon	Betsi Cadwaladr University Health Board
Katharine Burton	Betsi Cadwaladr University Health Board
Kim Storton	Bradford Teaching Hospitals NHS Foundation Trust
Malathi Munusamy	Bradford Teaching Hospitals NHS Foundation Trust
Bridget Tandy	Bradford Teaching Hospitals NHS Foundation Trust
Akamino Egbo	Bradford Teaching Hospitals NHS Foundation Trust
Stephen Cox	Bradford Teaching Hospitals NHS Foundation Trust
Nabeela Nazir Ahmed	Bradford Teaching Hospitals NHS Foundation Trust
Anil Shenoy	Bradford Teaching Hospitals NHS Foundation Trust
Rachel Bousfield	Department of Infectious Diseases, Cambridge University Hospitals NHS Foundation Trust

Donna Wixted	Dorset County Hospital
Helen Gutteridge	Dorset Research Hub
Becky Mansfield	Dorset Research Hub
Christopher Herbert	Leeds Teaching Hospitals NHS Trust
Jennifer Murira	Leeds Teaching Hospitals NHS Trust
James Calderwood	Leeds Teaching Hospitals NHS Trust
Dominique Barker	Leeds Teaching Hospitals NHS Trust
Jacqueline Brandon	Leeds Teaching Hospitals NHS Trust
Hayley Tulloch	Leeds Teaching Hospitals NHS Trust
Suzie Colquhoun	Leeds Teaching Hospitals NHS Trust
Helen Thorp	Leeds Teaching Hospitals NHS Trust
Helen Radford	Leeds Teaching Hospitals NHS Trust
Julie Evans	Leeds Teaching Hospitals NHS Trust
Helena Baker	Leeds Teaching Hospitals NHS Trust
Jeanette Thorpe	Leeds Teaching Hospitals NHS Trust
Sally Batham	Leicester CRF
Jessica Hailstone	Leicester CRF
Rachael Phillips	Leicester CRF
Dileep Kumar	Leicester CRF
Fran Westwell	Liverpool University Hospitals NHS Foundation Trust
Ann Sturdy	London Northwest University Healthcare
Lara Barcella	London Northwest University Healthcare

Najwa Soussi	London Northwest University Healthcare
Mushiya Mpelembue	London Northwest University Healthcare
Sreena Raj	London Northwest University Healthcare
Rajni Sharma	London Northwest University Healthcare
Tumena Corrah	London Northwest University Healthcare
Laurence John	London Northwest University Healthcare
Ashley Whittington	London Northwest University Healthcare
Siobhan Roche	National Institute of Health Research, West Midlands Clinical Research Network
Lynda Wagstaff	National Institute of Health Research, West Midlands Clinical Research Network
Adam Farrier	Newcastle University
Karen Bisnauthsing	NIHR BRC at Guy's and St Thomas' NHS Foundation Trust
Michael Singh	NIHR BRC at Guy's and St Thomas' NHS Foundation Trust
Movin Abeywickrama	NIHR BRC at Guy's and St Thomas' NHS Foundation Trust
Niamh Spence	NIHR BRC at Guy's and St Thomas' NHS Foundation Trust
Alice Packham	NIHR BRC at Guy's and St Thomas' NHS Foundation Trust
Teona Serafimova	NIHR BRC at Guy's and St Thomas' NHS Foundation Trust
Suahil Aslam	NIHR BRC at Guy's and St Thomas' NHS Foundation Trust
Caitlin McGreevy	NIHR BRC at Guy's and St Thomas' NHS Foundation Trust
Alessandro Borca	NIHR BRC at Guy's and St Thomas' NHS Foundation Trust
Pamela DeLosSantosDominguez	NIHR BRC at Guy's and St Thomas' NHS Foundation Trust
Emily Palmer	NIHR BRC at Guy's and St Thomas' NHS Foundation Trust
Samantha Broadhead	NIHR BRC at Guy's and St Thomas' NHS Foundation Trust

Sadaf Farooqi	NIHR Clinical Research Facility, Cambridge University Hospitals NHS Foundation Trust
Jo Piper	NIHR Clinical Research Facility, Cambridge University Hospitals NHS Foundation Trust
Rowena Weighell	NIHR Clinical Research Facility, Cambridge University Hospitals NHS Foundation Trust
Lorinda Pickup	NIHR Clinical Research Facility, Cambridge University Hospitals NHS Foundation Trust
Djamila Shamtally	NIHR Clinical Research Facility, Cambridge University Hospitals NHS Foundation Trust
Jason Domingo	NIHR Clinical Research Facility, Cambridge University Hospitals NHS Foundation Trust
Evgenia Kourampa	NIHR Clinical Research Facility, Cambridge University Hospitals NHS Foundation Trust
Colin Hale	NIHR Liverpool & Broadgreen CRF
Jennifer Gibney	NIHR Liverpool & Broadgreen CRF
Michael Stackpoole	NIHR Liverpool & Broadgreen CRF
Zalina Rashid-Gardner	NIHR Liverpool & Broadgreen CRF
Rebecca Lyon	NIHR Liverpool & Broadgreen CRF
Chloe McDonnell	NIHR Liverpool & Broadgreen CRF
Christine Cole	NIHR Liverpool & Broadgreen CRF
Anna Stewart	NIHR Liverpool & Broadgreen CRF
Gillian McMillan	NIHR Liverpool & Broadgreen CRF
Mary Savage	NIHR Liverpool & Broadgreen CRF
Helen Beckett	NIHR Liverpool & Broadgreen CRF
Chantelle Moorbey	NIHR Wessex Local Clinical Research Network
Amisha Desai	NIHR/Wellcome Clinical Research Facility, University Hospitals Birmingham NHS Foundation Trust
Claire Brown	NIHR/Wellcome Clinical Research Facility, University Hospitals Birmingham NHS Foundation Trust
Kush Naker	NIHR/Wellcome Clinical Research Facility, University Hospitals Birmingham NHS Foundation Trust

Karishma Gokani	NIHR/Wellcome Clinical Research Facility, University Hospitals Birmingham NHS Foundation Trust
Charlotte Trinham	NIHR/Wellcome Clinical Research Facility, University Hospitals Birmingham NHS Foundation Trust
Charlotte Sabine	NIHR/Wellcome Clinical Research Facility, University Hospitals Birmingham NHS Foundation Trust
Sophie Moore	NIHR/Wellcome Clinical Research Facility, University Hospitals Birmingham NHS Foundation Trust
Steve Hurdover	NIHR/Wellcome Clinical Research Facility, University Hospitals Birmingham NHS Foundation Trust
Edwin Justice	NIHR/Wellcome Clinical Research Facility, University Hospitals Birmingham NHS Foundation Trust
Megan Stone	Oxford Vaccine Group, Department of Paediatrics, University of Oxford, Oxford, UK
Emma Plested	Oxford Vaccine Group, Department of Paediatrics, University of Oxford, Oxford, UK
Carla Ferreira Da Silva	Oxford Vaccine Group, Department of Paediatrics, University of Oxford, Oxford, UK
Rachel White	Oxford Vaccine Group, Department of Paediatrics, University of Oxford, Oxford, UK
Hannah Robinson	Oxford Vaccine Group, Department of Paediatrics, University of Oxford, Oxford, UK
Iain Turnbull	Oxford Vaccine Group, Department of Paediatrics, University of Oxford, Oxford, UK
Gertraud Morshead	Oxford Vaccine Group, Department of Paediatrics, University of Oxford, Oxford, UK
Rachael Drake-Brockman	Oxford Vaccine Group, Department of Paediatrics, University of Oxford, Oxford, UK
Catherine Smith	Oxford Vaccine Group, Department of Paediatrics, University of Oxford, Oxford, UK
Grace Li	Oxford Vaccine Group, Department of Paediatrics, University of Oxford, Oxford, UK
Mwila Kasanyinga	Oxford Vaccine Group, Department of Paediatrics, University of Oxford, Oxford, UK
Elizabeth A Clutterbuck	Oxford Vaccine Group, Department of Paediatrics, University of Oxford, Oxford, UK
Sagida Bibi	Oxford Vaccine Group, Department of Paediatrics, University of Oxford, Oxford, UK
Trishna Champaneri	PHARMExcel
Margaret Irwin	PHARMExcel
Mohammed Khan	PHARMExcel

Alicja Kownacka	PHARMExcel
Martha Nabunjo	PHARMExcel
Carol Osuji	PHARMExcel
John Hladkiwskyj	PHARMExcel
Dominic Galvin	PHARMExcel
Gita Patel	PHARMExcel
Jacques Grierson	PHARMExcel
Samantha Males	PHARMExcel
Krishna Askoolam	PHARMExcel
Joshua Barry	PHARMExcel
Johanna Mouland	Portsmouth Hospitals University NHS Trust
Beverley Longhurst	Portsmouth Hospitals University NHS Trust
Maria Moon	Portsmouth Hospitals University NHS Trust
Beth Giddins	Portsmouth Hospitals University NHS Trust
Carlota Pereira Dias Alves	Portsmouth Hospitals University NHS Trust
Leah Richmond	Portsmouth Hospitals University NHS Trust
Christine Minnis	Portsmouth Hospitals University NHS Trust
Sonia Baryschpolec	Portsmouth Hospitals University NHS Trust
Scott Elliott	Portsmouth Hospitals University NHS Trust
Lauren Fox	Portsmouth Hospitals University NHS Trust
Victoria Graham	Public Health England
Natalie Baker	Public Health England

Kerry Godwin	Public Health England
Karen Buttigieg	Public Health England
Chanice Knight	Public Health England
Phillip Brown	Public Health England
Paminder Lall	Public Health England
Imam Shaik	Public Health England
Emily Chiplin	Public Health England
Emily Brunt	Public Health England
Stephanie Leung	Public Health England
Lauren Allen	Public Health England
Steve Thomas	Public Health England
Sara Fraser	Public Health England
Bea Choi	Public Health England
Jade Gouriet	Public Health England
Jonathan Perkins	Queen Elizabeth University Hospital, NHS Greater Glasgow & Clyde
Andrew Gowland	Queen Elizabeth University Hospital, NHS Greater Glasgow & Clyde
Jonathan Macdonald	Queen Elizabeth University Hospital, NHS Greater Glasgow & Clyde
John Paul Seenan	Queen Elizabeth University Hospital, NHS Greater Glasgow & Clyde
Igor Starinskij	Queen Elizabeth University Hospital, NHS Greater Glasgow & Clyde
Andrew Seaton	Queen Elizabeth University Hospital, NHS Greater Glasgow & Clyde
Erica Peters	Queen Elizabeth University Hospital, NHS Greater Glasgow & Clyde
Stephen Singh	Royal Devon and Exeter Hospital

Ben Gardside	Royal Devon and Exeter Hospital
Avril Bonnaud	Royal Devon and Exeter Hospital
Ceri Davies	Royal Devon and Exeter Hospital
Elizabeth Gordon	Royal Devon and Exeter Hospital
Samantha Keenan	Royal Devon and Exeter Hospital
Jane Hall	Royal Devon and Exeter Hospital
Suzanne Wilkins	Royal Devon and Exeter Hospital
Suzanne Tasker	Royal Devon and Exeter Hospital
Rob James	Royal Devon and Exeter Hospital
Ingrid Seath	Royal Devon and Exeter Hospital
Kelly Littlewood	Royal Devon and Exeter Hospital
Joseph Newman	Royal Papworth Hospital NHS Foundation Trust
Iryna Boubriak	Royal Papworth Hospital NHS Foundation Trust
Debbie Suggitt	Stockport NHS Foundation Trust
Helen Haydock	Stockport NHS Foundation Trust
Sara Bennett	Stockport NHS Foundation Trust
Wiesia Woodyatt	Stockport NHS Foundation Trust
Kerry Hughes	Stockport NHS Foundation Trust
Judith Bell	Stockport NHS Foundation Trust
Tricia Coughlan	Stockport NHS Foundation Trust
Donald van Welsenes	Stockport NHS Foundation Trust
Mohammed Kamal	Stockport NHS Foundation Trust

Chris Cooper	Stockport NHS Foundation Trust
Simon Tunstall	Stockport NHS Foundation Trust
Nicholas Ronan	Stockport NHS Foundation Trust
Rebecca Cutts	The Adam Practice
Tracey Dare	The Adam Practice
Dr Yee Ting Nicole Yim	University College London Hospitals NHS Foundation Trust
Sarah Whittley	University College London Hospitals NHS Foundation Trust
Shama Hamal	University College London Hospitals NHS Foundation Trust
Marivic Ricamara	University College London Hospitals NHS Foundation Trust
Kirsty Adams	University College London Hospitals NHS Foundation Trust
Holly Baker	University College London Hospitals NHS Foundation Trust
Kimberley Driver	University College London Hospitals NHS Foundation Trust
Nicola Turner	University College London Hospitals NHS Foundation Trust
Todd Rawlins	University College London Hospitals NHS Foundation Trust
Subarna Roy	University College London Hospitals NHS Foundation Trust
Marta Merida-Morillas	University College London Hospitals NHS Foundation Trust
Yukari Sakagami	University College London Hospitals NHS Foundation Trust
Antonette Andrews	University College London Hospitals NHS Foundation Trust
Lillian Goncalvescordeiro	University Hospital Southampton NHS Foundation Trust
Matthew Stokes	University Hospital Southampton NHS Foundation Trust
Wythehi Ambihapathy	University Hospital Southampton NHS Foundation Trust
Joanne Spencer	University Hospital Southampton NHS Foundation Trust

Nina Parungao	University Hospital Southampton NHS Foundation Trust
Lisa Berry	University Hospital Southampton NHS Foundation Trust
James Cullinane	University Hospital Southampton NHS Foundation Trust
Laura Presland	University Hospital Southampton NHS Foundation Trust
Amy Ross Russell	University Hospital Southampton NHS Foundation Trust
Sarah Warren	University Hospital Southampton NHS Foundation Trust
Jonathan Baker	University Hospital Southampton NHS Foundation Trust
Abigail Oliver	University Hospital Southampton NHS Foundation Trust
Amanda Buadi	University Hospital Southampton NHS Foundation Trust
Kim Lee	University Hospital Southampton NHS Foundation Trust
Louise Haskell	University Hospital Southampton NHS Foundation Trust
Rossana Romani	University Hospital Southampton NHS Foundation Trust
Ian Bentley	University Hospital Southampton NHS Foundation Trust
Tim Whitbred	University Hospital Southampton NHS Foundation Trust
Simon Fowler	University Hospital Southampton NHS Foundation Trust
John Gavin	University Hospital Southampton NHS Foundation Trust
Alan Magee	University Hospital Southampton NHS Foundation Trust
Tara Watson	University Hospital Southampton NHS Foundation Trust
Kari Nightingale	University Hospital Southampton NHS Foundation Trust
Phedra Marius	University Hospital Southampton NHS Foundation Trust
Eloise Summerton	University Hospital Southampton NHS Foundation Trust
Emily Locke	University Hospital Southampton NHS Foundation Trust

Thomas Honey	University Hospital Southampton NHS Foundation Trust
Aidan Lingwood	University Hospital Southampton NHS Foundation Trust
Anastasia de la Haye	University Hospital Southampton NHS Foundation Trust
Ryan Stephen Elliott	University Hospital Southampton NHS Foundation Trust
Karen Underwood	University Hospital Southampton NHS Foundation Trust
Mikayala King	University Hospital Southampton NHS Foundation Trust
Sharon Davies-Dear	University Hospital Southampton NHS Foundation Trust
Emily Horsfall	University Hospital Southampton NHS Foundation Trust
Olivia Chalwin	University Hospital Southampton NHS Foundation Trust
Holly Burton	University Hospital Southampton NHS Foundation Trust
Christopher J Edwards	University Hospital Southampton NHS Foundation Trust
Benjamin Welham	University Hospital Southampton NHS Foundation Trust
Kim Appleby	University Hospital Southampton NHS Foundation Trust
Emily Dineen	University Hospital Southampton NHS Foundation Trust
Sarah Garrahy	University Hospital Sussex NHS Trust
Fran Hall	University Hospital Sussex NHS Trust
Eleni Ladikou	University Hospital Sussex NHS Trust
Dee Mullan	University Hospital Sussex NHS Trust
Daniel Hansen	University Hospital Sussex NHS Trust
Marion Campbell	University Hospital Sussex NHS Trust
Filipa Dos Santos	University Hospital Sussex NHS Trust
Nicki Lakeman	University Hospitals Dorset

Debbie Branney	University Hospitals Dorset
Luke Vamplew	University Hospitals Dorset
Alison Hogan	University Hospitals Dorset
Jorden Frankham	University Hospitals Dorset
Martin Wiselka	University Hospitals of Leicester NHS Trust
Denny Vail	University Hospitals of Leicester NHS Trust
Victoria Wenn	University Hospitals of Leicester NHS Trust
Valerie Renals	University Hospitals of Leicester NHS Trust
Kate Ellis	University Hospitals of Leicester NHS Trust
Jessica Lewis-Taylor	University Hospitals of Leicester NHS Trust
Haniah Habash-Bailey	University Hospitals Sussex NHS Trust
Javier Magan	Wessex Clinical Research network
Anna Hardy	Wessex Clinical Research network
