# Supplementary material for: Safety, immunogenicity, and reactogenicity of BNT162b2 and mRNA-1273 COVID-19 vaccines given as fourth-dose boosters following two doses of ChAdOx1 nCoV-19 or BNT162b2 and a third dose of BNT162b2 (COV-BOOST): a multicentre, blinded, phase 2, randomised trial
Source: Lancet Infect Dis. 2022 Aug;22(8):1131–41. doi: 10.1016/S1473-3099(22)00271-7 (PMC9084623; doi:10.1016/S1473-3099(22)00271-7)
Supplement: Supplementary appendix 2 [file mmc2.pdf]

THE LANCET
Infectious Diseases

Supplementary appendix 2

This appendix formed part of the original submission and has been peer reviewed. We post it as supplied by the authors.

Supplement to: Munro APS, Feng S, Janani L, et al. Safety, immunogenicity, and reactogenicity of BNT162b2 and mRNA-1273 COVID-19 vaccines given as fourth-dose boosters following two doses of ChAdOx1 nCoV-19 or BNT162b2 and a third dose of BNT162b2 (COV-BOOST): a multicentre, blinded, phase 2, randomised trial. *Lancet Infect Dis* 2022; published online May 9. https://doi.org/10.1016/S1473-3099(22)00271-7.

COV-BOOST
Evaluating COVID-19 vaccine boosters

University of Southampton | NHS University Hospital Southampton NHS Foundation Trust

Study Title: A randomised, phase II UK multi-centre study to determine reactogenicity and immunogenicity of booster vaccination against ancestral and novel variants of SARS-CoV-2

Short Title: Evaluating COVID-19 Vaccine Boosters

IRAS Project ID: 299180

EudraCT Number: 2021-002175-19

UHS Study Number: RHM MED1781

Date and Version No: V10.0 28th February 2022

Chief Investigator: Professor Saul Faust
NIHR Southampton Clinical Research Facility
University Hospital Southampton NHS Foundation Trust,
Southampton SO16 6YD

Investigators:

Sponsor: University Hospital Southampton NHS Foundation Trust

Funder: National Institute Health Research (NIHR), supported by the Vaccine Task Force and DHSC, and CEPI (fractional dose sub-study)

Chief Investigator Signature:

Sponsor Signature:

Statistician Signature:

1 Contents

2 Key Trial Contacts 10

3 Lay Summary 13

4 Background 14

5 Study Rationale 14

6 Synopsis 15

7 Abbreviations 23

8 Risks and Benefits 25

8.1 Potential benefits 25

8.2 Potential risks 26

8.2.1 Associated with phlebotomy 26

8.2.2 Associated with saliva sampling 26

8.2.3 Associated with nasal fluid sampling 26

8.2.4 Allergic reactions 26

8.2.5 Behaviour change 26

8.2.6 Specific risks from vaccines 26

8.2.7 Antibody-dependent enhancement and immunopathology 26

8.2.8 Unwanted media attention 27

8.2.9 Reactogenicity 27

9 Study Design 27

9.1 Trial Design 27

9.1.1 Setting 27

9.1.2 Trial duration 27

9.1.3 Study groups 28

9.1.4 Stage 1 28

9.1.5 Stage 2 (optional group, not funded at time of stage 1 submissions and set up) 29

9.1.6 Stage 3 30

9.1.7 Stage 1 Group allocations 30

9.1.8 Stage 2 Group allocations 33

9.1.9 Stage 3 Group allocations 33

9.1.10 Routine NHS deployment of booster vaccination 33

9.1.11 External Vaccine trial participants Sub-Study 34

9.1.12 Young Adult Fractional Dosing Sub-Study 35

9.1.13 Fourth Dose Booster Sub-Study 35

9.1.14 Omicron Specific Fourth Dose Booster sub-study 35

10 Study participants 36

10.1 Trial Participants 36

10.2 Inclusion Criteria 36

10.3 Exclusion Criteria 37

10.3.1 Temporary exclusion criteria 38

10.4 Eligibility for External Vaccine Trial Participants Sub-Study 38

11 Trial procedures 40

11.1 Recruitment 40

11.1.1 Volunteer Identification 40

11.2 Screening and Eligibility Assessment 41

11.2.1 Initial screening – Main Trial Only 41

11.2.2 Telephone screening visit(s) – Main Trial Only 42

11.2.3 Screening during Visit 1 42

11.2.4 Screening: External Vaccine Trial Sub-Study 42

11.3 Informed Consent 42

11.4 Randomisation 44

11.5 Blinding and code-breaking 45

11.5.1 Unblinding of all Stage 1 trial participants (protocol V7.0) 45

11.6 Visits 46

11.6.1 Visit 1 (D0): Final eligibility check, Enrolment and Vaccination visit 46

11.6.2 Safety review 48

11.6.3 Subsequent visits 49
11.6.4 Participants under quarantine 49
11.6.5 Participants with confirmed SARS-CoV-2 infection (COVID-19 Pathway) 49
11.6.6 Admission of participants to hospital with COVID-19 infection 51
11.7 Sample Handling 52
11.7.1 Sample handling for trial purposes 52
11.7.2 Sample handling for standard of care 53
11.8 Early Discontinuation/Withdrawal of Participants 53
11.9 Definition of End of Trial 54
12 Trial Interventions 54
12.1 Investigational Medicinal Product(s) (IMP) Description 54
12.1.1 AstraZeneca COVID-19 vaccine (ChAdOx1 nCOV-19) 55
12.1.2 Pfizer BioNTech (BNT162b2) 55
12.1.3 Moderna (mRNA-1273) 56
12.1.4 Novavax (NVXCoV2373) 56
12.1.5 Valneva (VLA2001) 57
12.1.6 Curevac (CVnCoV) 57
12.1.7 Janssen (Ad26.COV2.S) 58
12.1.8 Control vaccine 58
12.1.9 Blinding of IMPs 59
12.1.10 Storage of IMP 59
12.1.11 Compliance with Trial Treatment 60
12.1.12 Accountability of the Trial Treatment 60
12.1.13 Concomitant Medication 60
12.1.14 Post-trial Treatment 61
12.2 Other Treatments (non-IMPS) 61
12.3 Other Interventions 61
13 Safety Reporting 61

13.1 Safety reporting window 61
13.2 Adverse Event Definitions 62
13.3 Assessment results outside of normal parameters as AEs and SAEs 63
13.3.1 Clinical 63
13.3.2 Laboratory 63
13.4 Assessment of severity 64
13.5 Assessment of Causality 65
13.5.1 Causality assessment for participants who have received additional external vaccines 66
13.6 Procedures for Reporting Adverse Events 67
13.6.1 Solicited AEs 67
13.6.2 Unsolicited AEs 67
13.6.3 Medically attended AEs 67
13.7 Reporting Procedures for Serious Adverse Events 67
13.7.1 Events exempt from immediate reporting as SAEs 68
13.8 Expectedness 68
13.8.1 SAEs 68
13.8.2 Thrombocytopaenic thrombosis following adenovirus vector vaccination 74
13.8.2.1 ChAdOx1-nCov19 (Oxford) 74
13.8.2.2 Ad26.COV2.S (Janssen) 74
13.8.3 Disease enhancement following vaccination 75
13.9 SUSAR Reporting 75
13.10 Development Safety Update Reports 75
13.11 Interim reviews 75
13.12 Safety Holding Rules 76
13.13 Contraception and pregnancy 76
13.13.1 Contraception 76
13.13.2 Pregnancy 76

14 Statistics 77
14.1 Sample size 77
14.2 Description of statistical methods 78
14.3 Interim analyses 79
14.4 Missing data 80
15 Data Management 80
15.1 Access to Data & Data Protection 80
15.2 Data Recording 80
15.3 Record keeping 81
15.4 Source Data and Case Report Forms (CRFs) 82
15.5 Data Quality 82
15.6 Data Sharing 82
16 Quality Assurance Procedures 83
16.1 Risk assessment 83
16.2 Monitoring 83
16.3 Trial committees 83
16.3.1 Trial Steering Committee 83
16.3.2 Data Safety Monitoring Board 83
16.3.3 Study Management Group 83
17 Protocol Deviations 84
18 Serious Breaches 84
19 Ethical And Regulatory Considerations 84
19.1 Declaration of Helsinki 84
19.2 Guidelines for Good Clinical Practice 84
19.3 Approvals 84
19.4 Other Ethical Considerations 85
19.5 Reporting 85
19.6 Transparency in Research 85

19.7 Participant Confidentiality 85

19.8 Expenses and Benefits 86

20 Finance And Insurance 86

20.1 Funding 86

20.2 Contractual arrangements 86

21 Publication Policy 86

22 Development Of A New Product/ Process Or The Generation Of Intellectual Property 87

23 Archiving 87

24 References 87

25 Appendix A: Schedule of Visitation 89

25.1 Schedule of visits for general cohort 89

25.2 Schedule of visits for immunology cohorts 90

25.3 Schedule of additional visits for unblinded control arms in event of NHS national booster campaign 91

25.4 Schedule of visits for External vaccine trials participant 92

26 Appendix B: Amendment History 93

27 Appendix C: Toxicity grading scale for lab AEs 102

28 Appendix D: Blood Sampling 103

29 Appendix E: Young Adult Fractional Dosing Sub-Study 111

29.1 Rationale 111

29.2 Participants and eligibility 111

29.2.1 Inclusion Criteria 111

29.2.2 Exclusion Criteria 111

29.2.3 Temporary exclusion criteria 112

29.3 Trial design 113

29.3.1 Young Adults Fractional Dosing Sub-Study group allocation 113

29.4 Randomisation and blinding 113

29.5 Statistics 114

29.6 Schedule of visitation (Young Adult Fractional Dosing) 117

29.7 Blood sampling (Young Adult Fractional Dosing) Immunology Cohort 118

29.8 Blood sampling (Young Adult Fractional Dosing) General Cohort with Lithium Heparin 119

29.9 Blood sampling (Young Adult Fractional Dosing) General Cohort without Lithium Heparin 120

30 Appendix F: Fourth Dose Booster Sub-Study 121

30.1 Rationale 121

30.2 Participants and eligibility 121

30.3 Trial Design 121

30.3.1 Fourth Dose Booster Sub-Study group allocation 122

30.4 Randomisation and blinding 122

30.5 Statistics 122

30.6 Schedule of visitation (Fourth Dose Booster) 124

30.7 Blood sampling (Fourth Dose Booster) Immunology Cohort 125

30.8 Blood sampling (Fourth Dose Booster) General Cohort with Lithium Heparin 126

30.9 Blood sampling (Fourth Dose Booster) General Cohort without Lithium Heparin 127

31 Appendix G: Omicron Specific Fourth Dose Booster 128

31.1 Rationale 128

31.2 Outcomes 128

31.3 Participants and eligibility 128

31.3.1 Inclusion Criteria 128

31.3.2 Exclusion Criteria 129

31.3.3 Temporary exclusion criteria 130

31.4 Trial Design 130

31.4.1 Omicron Variant Booster Sub-Study group allocation 131

31.5 Randomisation and blinding 131

31.6 Statistics 131

31.7 Trial Interventions 133

31.7.1 Investigational Medicinal Product(s) (IMP) Description 133
31.8 Safety 134
31.8.1 Expected Serious Adverse Reactions 134
31.8.2 Adverse Events of Special Interest (AESIs) 134
31.9 Study Procedures 137
31.9.1 Schedule of visitation (Omicron Variant Booster Sub-study) 137
31.9.2 Blood sampling (Omicron Variant Booster) Immunology Cohort 138
31.9.3 Blood sampling (Omicron Variant Booster) General Cohort with Lithium Heparin ... 139
31.9.4 Blood sampling (Omicron Variant Booster) General Cohort without Lithium Heparin 140

2 Key Trial Contacts

**Chief Investigator**	Professor Saul Faust<br>Professor of Paediatric Immunology and Infectious Diseases<br>NIHR Southampton Clinical Research Facility<br>University Hospital Southampton NHS Foundation Trust<br>Tremona Road, Southampton<br>United Kingdom<br>s.faust@soton.ac.uk
**Sponsor**	Sharon Davies-Dear<br>University Hospital Southampton NHS Foundation Trust<br>Tremona Road<br>Southampton<br>Hampshire, SO16 6YD<br>023 8120 5146<br>Email: Sharon.davies-dear@uhs.nhs.uk
**Funder(s)**	National Institute for Health Research & UK Vaccine Task Force<br>Young Adult Fractional Dosing sub-study funded by the Centre for Epidemic Preparedness and Innovation (CEPI)
**Clinical Trials Unit**	Oxford Vaccine Group,<br>University of Oxford,<br>Centre for Clinical Vaccinology and Tropical Medicine (CCVTM),<br>Churchill Hospital,<br>Oxford, OX3 7LE,<br>United Kingdom<br>info@ovg.ox.ac.uk

**External Contract Research Organisation (CRO)**	PHARMExcel Ltd<br>Albany Chambers<br>26 Bridge Road East<br>Welwyn Garden City<br>Hertfordshire<br>AL7 1HL<br>United Kingdom
**Statisticians**	**Xinxue Liu**<br>Oxford Vaccine Group, Centre for Clinical Vaccinology and Tropical Medicine (CCVTM),<br>University of Oxford, Churchill Hospital, Oxford, OX3 7LE,<br>xinxue.liu@paediatrics.ox.ac.uk<br>**Nick Andrews**<br>Public Health England<br>61 Colindale Ave, London NW9 5EQ<br>nick.andrews@phe.gov.uk<br>**Leila Janani**<br>Imperial College London<br>Exhibition Rd, South Kensington, London SW7 2BX<br>l.janani@imperial.ac.uk<br>**Victoria Cornelius**<br>Imperial College London<br>Exhibition Rd, South Kensington, London SW7 2BX<br>v.cornelius@imperial.ac.uk

**Committees**	**Data Safety Monitoring Board**<br>Chair: Prof Andrew Ustianowski, North Manchester General Hospital<br>Statistician: Prof Chris Rogers, University of Bristol<br>Clinical vaccine expert: Dr Andrew Riordan, Alder Hey Hospital Liverpool.<br>Open meetings: CI, Sponsor Representatives, PHARMExcel representative.<br><br>**Trial Steering Group**<br>Chair: Prof Rob Read, University of Southampton<br>Prof Mary Ramsay, Public Health England<br>Dr Paul Turner, Imperial College London<br>Prof Claire Cameron, University College London<br>Dr Karen Underwood (Sponsor Rep), UHSFT<br>Prof Chris Kipps (Sponsor Rep), UHSFT<br>Yvanne Enever (CRO Rep), PHARMExcel<br>**Study Management Group**<br>S Faust (CI)<br>Site PIs:<br>A Minassian (or delegate)<br>K Chatterjee (or delegate)<br>D Saralaya (or delegate)<br>A Goodman (or delegate)<br>C Twelves (or delegate)<br>M Bula (or delegate)<br>E Thomson (or delegate)<br>A Mcgregor (or delegate)<br>O Osanlou (or delegate)<br>R Sheridan (or delegate)<br>D Baxter (or delegate)<br>V Libri (or delegate)<br>K Cathie (or delegate)<br>C Green (or delegate)<br>A Palfreeman (or delegate)<br>S Sharma (or delegate)<br>P Heath (or delegate)

	Project Management:<br><br>S Saich (Senior Vaccine Project Manager)<br>S Davies-Dear (Sponsor Representative)<br>PHARMexcel (CRO) project managers

3 Lay Summary

As of 1st June 2021 the MHRA in the UK has granted Regulation 174 approval for emergency use of 4 vaccines for protection against COVID-19 in the UK, including the mRNA vaccines BNT162b2 (Pfizer) the mRNA-1273 (Moderna) vaccine, the chimpanzee adenovirus vector vaccine ChAdOx1-nCov19 (AstraZeneca/Oxford), and the human adenovirus vector vaccine Ad26.COV2.S (Janssen). So far over 33 million people in the UK have received at least one dose of either BNT162b2, ChAdOx1-nCov19, or mRNA-1273. Annual or seasonal booster vaccination for high risk groups is thought likely to be required, especially in light of the emergence of new variants of the SARS-CoV-2 virus. There is concern from studies in South Africa and elsewhere that existing vaccines may be less effective against these variant strains. It is currently unclear which booster vaccine schedule will provide the best safety profile and immune responses, according to which vaccine was originally given. The Joint Committee for Vaccination and Immunisation and UK Chief Medical Officers need timely information regarding the effects of different booster vaccinations on the safety profile and immunity to previous and new variants of SARS-CoV-2 in order to inform national policy for autumn and winter 2021. This study will determine the immune responses provided from different booster vaccinations given a minimum of 3 months from the 2nd dose of an initial course of AstraZeneca/Oxford or Pfizer vaccines.

4 Background

The COVID-19 pandemic was met with a rapid global effort to produce safe and efficacious vaccines to protect vulnerable individuals against severe disease, and to reduce transmission of the virus. Within a year there had been multiple vaccine candidates which were successfully granted emergency use authorisation by several high stringency agencies on the bases of successful clinical safety and efficacy endpoints in phase 3 clinical trials.

Whilst immunity conferred by natural infection has been shown to by durable for many months [1], long term immunogenicity data is not yet available for vaccines beyond 6 months [2]. There is an expectation that the durability of immune response may be affected by 2 factors; the waning over time of immune memory elicited by vaccines, and the emergence of novel variants of the SARS-Cov-2 virus with substantive mutations to the spike protein which enable varying levels of antigen escape [3, 4]. This is likely to necessitate booster doses of COVID-19 vaccines, and potentially periodic boosters. Boosters will either increase personal protection via generating higher antibody levels against the original pandemic variant regardless of circulating variant; or in due course by generating immunity specifically against novel variants of the virus.

5 Study Rationale

The 2 vaccines which have been delivered at scale in the UK so far have been the mRNA vaccine BNT162b2 (Pfizer) and the chimpanzee adenovirus vector vaccine ChAdOx1-nCov19 (Oxford/AstraZeneca). Studies are underway currently to examine the effect of heterologous 2 dose primary regimens using these differing vaccine technologies (the COM-COV trial, IRAS Project ID: 291055 EudraCT Number: 2020-005085-33).

The COV-BOOST trial aims to establish the safety and immunogenicity of seasonal boosting vaccination with different authorised vaccine candidates for participants who have received homologous prim-boosting with either BNT162b2 or ChAdOx1-nCov19 early in the UK NHS deployment campaign. In addition, this study will examine the safety and efficacy of boosting using vaccines designed specifically to target novel variants of the SARS-CoV-2 virus when these vaccines become available.

6 Synopsis

Trial Title	A randomised, phase II UK multi-centre study to determine reactogenicity and immunogenicity of booster vaccination against SARS-CoV-2
Internal ref. no. (or short title)	Evaluating COVID-19 Vaccine Boosters (COV-BOOST)
Trial registration	EudraCT: 2021-002175-19<br><br>ISRCTN: ISRCTN73765130
Sponsor	University Hospital Southampton NHS Foundation Trust<br><br>Tremona Road<br><br>Southampton<br><br>SO16 6YD
Funder	National Institute for Health Research
Clinical Phase	Phase II
Trial Design	Three stage, randomized, adaptive phase II multicentre study of annual booster vaccination against ancestral and novel variants of SARS-CoV-2. Participants and laboratory staff will remain blind to treatment allocation. Analysing statisticians will be blinded for any stages or sub-stages after sign off of Statistical Analysis Plan (SAP) V1.0
Trial Participants	Adults aged 30 years or older who received a complete homologous 2 dose primary course of vaccination against COVID-19 with their first dose in December 2020, January or February 2021, and their booster at least 84 days prior to day 0 (70 days minimum allowable with Sponsor approval at site). Recruitment will be from participants aged 30 years and older (including specifically those aged 75 years and over).<br><br>Participants that have taken part in the Phase III NVXCoV2373 clinical trial are eligible for enrolment to the external vaccine trial sub-study of this protocol.
Sample Size	**Existing SARS-CoV-2 strain booster (stage 1)**<br><br>Stage 1 will be run across 3 groups (A-C) which will each be enrolled at different clusters of sites, with a total of 2886 participants. Site groups A and C will have 888 participants consisting of a cohort of

	444 participants who received 2 doses of BNT162b2 with the second dose at least 3 months (=>84 days) prior to enrolment and 444 participants who received 2 doses of ChAdOx1 nCov19 with the second dose at least 3 months (=>84 days) prior to enrolment. Site group B will have 1110 participants consisting of a cohort of 555 participants who received 2 doses of BNT162b2 with the second dose at least 3 months (=>84 days) prior to enrolment and 555 participants who received 2 doses of ChAdOx1-nCov19 with the second dose at least 3 months (=>84 days) prior to enrolment. Total target enrolment figures and the target number of participants per arm are approximates, some minor variation is expected as a result of randomisation stratification.<br><br>**Site Group A**<br><br>Each of 2 cohorts of 444 participants will be randomised 1:1:1:1 to receive a single dose of the following vaccines:<br><br>• ChAdOx1 nCOV-19 (111 participants)<br>• Novavax (111 participants)<br>• Novavax half dose⁺ (111 participants)<br>• Men ACWY (control) (111 participants)<br><br>**Site Group B**<br><br>Each of 2 cohorts of 555 participants will be randomised 1:1:1:1:1 to receive a single dose of the following vaccines:<br><br>• BNT162b2 (111 participants)<br>• VLA2001 (111 participants)<br>• VLA2001 half dose⁺ (111 participants)<br>• Ad26.COV2.S (111 participants)<br>• MenACWY (111 participants)<br><br>**Site Group C**<br><br>Each of 2 cohorts of 444 participants will be randomised 1:1:1:1 to receive a single dose of the following vaccines:<br><br>• mRNA-1273 (111 participants)<br>• CVnCoV (111 participants)<br>• BNT162b2 half dose⁺ (111 participants)<br>• Men ACWY (111 participants)<br><br>**Existing SARS-CoV-2 strain booster (stage 2, not yet agreed/funded, left in protocol now for information re how the trial might evolve during the pandemic)**<br><br>This adaptive and optional stage 2 is in preparation for vaccines which are not authorized or available for use in the UK at the time Stage 1 begins (this may include vaccines currently planned for Stage 1).

	As new vaccines are provided an emergency use authorization by MHRA they will be assigned a new group of 111 participants in each cohort as in stage 1 (For each vaccine, 111 people who have 2 doses of BNT162b2 with the second dose at least 3 months (=>84 days) prior to enrolment and 111 participants who received 2 doses of ChAdOx1 nCov19 with the second dose at least 3 months (=>84 days) prior to enrolment. A further 111 control participants will receive Men ACWY. Total target enrolment figures and the target number of participants per arm are approximates, some minor variation is expected as a result of randomisation stratification.<br><br>Each of 2 cohorts of 222 participants will be randomised 1:1(:1) to receive a single dose of the following vaccines:<br>• Vaccine G (111 participants)<br>• Men ACWY (control) (111 participants)<br><br>If other vaccines become available in the interim, a planned amendment will be added to add 2 additional groups with 111 participants each (who have each received 2 doses of BNT162b2 or ChAdOx1 nCov19).<br><br>**Novel variant booster** Please see Appendix G for details of the Omicron variant booster sub-study.<br><br>This adaptive stage 3 is in preparation for future viral variant vaccines.		
Planned Trial Period	12 months per participant in the main study (following on from the first vaccination)<br><br>6 – 10 months per participant in the external vaccine trial sub-study<br><br>Total trial period 1 year, 6 months		
	Objectives	Outcome Measures	Timepoint(s)
Co-Primary	To determine the safety and reactogenicity of annual booster doses of vaccines against SARS-CoV-2 following a homologous 2 dose primary regime	Solicited and unsolicited adverse events<br><br>Serious adverse events<br><br>Adverse events of special interest	Solicited adverse events: Day 0-7 after immunisation<br><br>Unsolicited adverse events: Day 0-28 after immunisation<br><br>Serious adverse events: throughout the study<br><br>Adverse events of special interest: throughout the study

Co-Primary	To determine whether the immune response to booster immunisation with different COVID-19 vaccines is superior to control vaccination for participants who have received priming vaccination with either ChAdOx1-nCov19 or BNT162b2	Immunogenicity: Anti spike protein IgG	Day 28
Secondary	Further characterisation of immunogenicity of booster vaccination against ancestral and novel variants of SARS-CoV-2	Anti-spike immunoglobulins	Day 0, 7*, 28, 84, 242
		Neutralising antibodies against SARS-CoV-2	Day 0, 28, 84, 242
		Anti-nucleocapsid immunoglobulins	Day 0, 84, 242
		Pseudo neutralising antibodies	Day 0, 28, 84, 242
		Cellular immune responses by ELISpot**	Day 0, 14*, 28, 84, 242
		Cellular immune responses by ICS (Th1/Th2)**	D0*, 14*
	To characterise immune responses of 3rd dose booster vaccinations when administered approximately 6 months after 2nd dose, and in comparison to those administered after 3 months	Anti-spike immunoglobulins, neutralising and pseudo-neutralising antibodies, cellular immune response by ICS** and ELISpot**	Post unblinding of the control vaccine arms: UCD0, UCD14, UCD28, UCD242
	To assess changes in markers of cardiac muscle damage in participants receiving a 3rd dose COVID-19 booster vaccine	High sensitivity Troponin	Post unblinding of the control vaccine arms: UCD0, UCD14

	approximately 6 months after 2nd dose, post initial vaccination course		
	To characterise immune responses of 3rd dose booster vaccination with BNT162b2 when administered after a primary immunization course of purified protein technology (NVXCoV2373)	Anti-spike immunoglobulins, neutralising antibodies, cellular immune response by ICS** and ELISpot**	Day 0, 14, 28, 84, 242
	To characterise immune responses of 3rd dose booster vaccination with fractional doses of BNT162b2 and mRNA-1273 when administered to young adults aged 18 – 30y	Anti-spike immunoglobulins, neutralising and pseudo-neutralising antibodies, cellular immune response by ICS** and ELISpot**	Day 0, 14, 28, 84, 242
	To characterise immune responses of 4th dose booster vaccination with BNT162b2 and mRNA-1273 50mcg when administered to participants who previously received a 3rd dose of BNT162b2	Anti-spike immunoglobulins, neutralising antibodies, cellular immune response by ICS** and ELISpot**	Day 0, 14, 84,
	To characterise immune responses of 4th dose booster vaccination with BNT162b2 and omicron variant vaccine mRNA-1273.529 50mcg when administered to adults aged 30 years or over	Anti-spike immunoglobulins, neutralising antibodies, cellular immune response by ICS** and ELISpot**	Day 0, 14, 28, 84, 242
	Safety of booster vaccination against ancestral and novel variants of SARS-CoV-2	Medically attended adverse events	Up to 3 months post immunisation
		Changes from baseline in laboratory safety measures	Day 0, 7*, 28,

	To determine the reactogenicity of 3rd dose booster vaccinations when administered approximately 6 months after 2nd dose, and in comparison to those administered after 3 months	Solicited and unsolicited adverse events<br>Medically attended adverse events	Solicited adverse events: Day 0-7 after immunisation<br>Unsolicited adverse events: Day 0-28 after immunisation<br>Up to 3 months post immunisation
	To determine the reactogenicity of 3rd dose booster vaccination with BNT162b2 when administered after a primary immunisation course of purified protein technology (NVXCoV2373)	Solicited and unsolicited adverse events<br>Medically attended adverse events	Solicited adverse events: Day 0-7 after immunisation<br>Unsolicited adverse events: Day 0-28 after immunisation<br>Up to 3 months post immunisation
	To determine the reactogenicity of 3rd dose booster vaccination with fractional doses of BNT162b2 and mRNA-1273 when administered to young adults aged 18 – 30y	Solicited and unsolicited adverse events<br>Medically attended adverse events	Solicited adverse events: Day 0-7 after immunisation<br>Unsolicited adverse events: Day 0-28 after immunisation<br>Up to 3 months post immunisation
	To determine the reactogenicity of 4th dose booster vaccination with BNT162b2 and mRNA-1273 50mcg when administered to participants who previously received a 3rd dose of BNT162b2	Solicited and unsolicited adverse events<br>Medically attended adverse events	Solicited adverse events: Day 0-7 after immunisation<br>Unsolicited adverse events: Day 0-28 after immunisation

			Up to 3 months post immunisation
	To determine the reactogenicity of 4th dose booster vaccination with BNT162b2 and omicron variant vaccine mRNA-1273.529 when administered to adults aged 30 years or over.	Solicited and unsolicited adverse events<br>Medically attended adverse events	Solicited adverse events: Day 0-7 after immunisation<br>Unsolicited adverse events: Day 0-28 after immunisation<br>Up to 3 months post immunisation
Exploratory	To characterise COVID-19 infections experienced following booster vaccination against ancestral and novel variants of SARS-CoV-2	Anti-spike & anti-nucleocapsid immunoglobulins, neutralising and pseudo-neutralising antibodies, cellular immune response by ICS and ELISpot<br>Genome sequencing of SARS-CoV-2 viruses isolated from infected participants	From vaccination, and within 1 week of a participant being found to be SARS-CoV-2 positive by external testing
	To further characterise the blood antibody response to booster vaccination against ancestral and novel variants of SARS-CoV-2	Functional antibody assays in line with other NISEC studies.	Day 0, 28, 242
	To characterise and compare the mucosal immune response to immunisation with homologous and heterologous COVID-19 vaccines in the immunology cohort using nasal fluid (collected using SAM-strips) and saliva samples. *	IgA & IgG ELISA and exploratory immunological assays	Day 0, 28, 242

* Only applies to the immunology cohort comprising approximately 25 participants from each group

** Only applies to centres collecting Lithium Heparin blood samples (approximately 50% of participants

[+] Half doses of vaccine have been included to investigate as potential dose sparing regimen for use in the UK or globally due to potential shortage of vaccine supply

Intervention(s)

- IMP(s)

Vaccine	Dose	Route of administration
AstraZeneca COVID-19 vaccine AZD1222 (ChAdOx1 nCOV-19)	5x10^{10}vp (0.5ml)	Intramuscular
Pfizer BioNTech (BNT162b2)	30 µg (0.3ml)	Intramuscular
Pfizer BioNTech (BNT162b2)	15 µg (0.15ml)	Intramuscular
Pfizer BioNTech (BNT162b2)	10 µg (0.1ml)	Intramuscular
NVX–CoV2373, Novavax	5 µg SARS-CoV-2 rS + 50 µg Matrix-M1 adjuvant (0.5ml)	Intramuscular
NVX–CoV2373, Novavax Half Dose[+]	2.5 µg SARS-CoV-2 rS + 25 µg Matrix-M1 adjuvant (0.25ml)	Intramuscular
VLA2001, Valneva	33 AU + 0.5mg Aluminium hydroxide +1mg CPG (0.5ml)	Intramuscular
VLA2001, Valneva Half Dose[+]	16.5 AU + 0.25mg Aluminium hydroxide + 0.5mg CPG (0.25ml)	Intramuscular

		mRNA 1273, Moderna	0.10 mg (0.5ml)	Intramuscular
		mRNA 1273, Moderna	0.05 mg (0.25ml)	Intramuscular
		CVnCoV, Curevac	12 µg (0.6ml)	Intramuscular
		Janssen, Ad26COV2.S	$5x10^{10}$vp (0.5ml)	Intramuscular
		Men ACWY	0.5ml	Intramuscular
		mRNA-1273.529, Moderna Omicron variant	50 µg (0.25ml)	Intramuscular

[+] Half doses of vaccine have been included to investigate as potential dose sparing regimen for use in the UK or globally due to potential shortage of vaccine supply

7 Abbreviations

ADE	Antibody Dependant Enhancement
AE	Adverse event
AESI	Adverse Event of Special Interest
AR	Adverse reaction
AU	Antigen Units
C-19P	COVID-19 Pathway
ChAdOx1	Chimpanzee adenovirus 1
CI	Chief Investigator
CRF	Case Report Form
CT	Clinical Trials
CTA	Clinical Trials Authorisation
CTRG	Clinical Trials and Research Governance
DSMB	Data Safety Monitoring Board

DSUR	Development Safety Update Report
EDC	Electronic Data Capture
ELISPOT	Enzyme-linked Immunospot
FBC	Full blood count
GCP	Good Clinical Practice
GMT	Geometric Mean Titre
GP	General Practitioner
HIV	Human Immunodeficiency virus
HRA	Health Research Authority
IB	Investigators Brochure
ICS	Intracellular Cytokine Staining
ICF	Informed Consent Form
IM	Intramuscular
IMP	Investigational Medicinal Product
IV	Intravenous
JCVI	Joint Committee on Vaccination and Immunisation
MHRA	Medicines and Healthcare products Regulatory Agency
NHS	National Health Service
NIHR	National Institute for Health Research
NISEC	National Immunisation Schedule Evaluation Consortium
PBMC	Peripheral blood mononuclear cell
PCR	Polymerase chain reaction
qPCR	Quantitative polymerase chain reaction
RES	Research Ethics Service
PB	Post-booster
PI	Principal Investigator

PIS	Participant/ Patient Information Sheet
REC	Research Ethics Committee
RSI	Reference Safety Information
SAE	Serious Adverse Event
SAM-strips	Synthetic absorbable matrix strips
SAR	Serious Adverse Reaction
SMPC	Summary of Medicinal Product Characteristics
SOP	Standard Operating Procedure
SUSAR	Suspected Unexpected Serious Adverse Reactions
TMF	Trial Master File
TSG	University Hospital Southampton NHS Foundation Trust Trials Steering Group
UHS NHSFT	University Hospital Southampton NHS Foundation Trust
µg	Microgram
Vp	Viral particle
VTF	Vaccine Task Force
WHO	World Health Organisation

8 Risks and Benefits

8.1 Potential benefits

Participants in this study will have already received a 2 dose primary regime of an approved COVID-19 vaccine through the NHS. Administration of the third dose 'boost' of COVID-19 vaccine in this study (from 84 days post dose 2) may be administered earlier than it would be through routine immunisation which is of potential benefit.

It is hoped that the information gained from this study will contribute to the development of a safe, effective and versatile vaccine programme against COVID-19.

8.2 Potential risks

8.2.1 Associated with phlebotomy

Localised bruising and discomfort can occur at the site of venepuncture. Infrequently fainting may occur. These will not be documented as AEs if they occur. The total volume of blood drawn over a 12 month period will be up to 311ml (+ up to 67ml per COVID-19 visit if required, and/or up to 7ml per additional set of safety bloods, + up to 164ml if in the control arm Immunology cohort attending for unblinded booster vaccination) (blood volumes may vary slightly for participants at different investigator sites due to use of different volume vacutainers, following local Trust SOPs). This should not compromise these otherwise healthy volunteers, as these volumes are within the limits of 470mL every 3 – 4 months for blood donations to the National Blood Transfusion Service. Participants will be asked to refrain from blood donation for the duration of their involvement in the trial.

8.2.2 Associated with saliva sampling

Participants may find the saliva collection process unsavoury as it is involves drooling and spitting.

8.2.3 Associated with nasal fluid sampling

Localised discomfort can occur in the nostril. Infrequently, this can result in a small amount of epistaxis, which can be controlled with pressure to the affected area.

8.2.4 Allergic reactions

Allergic reactions from mild to severe may occur in response to any constituent of a medicinal product's preparation. Anaphylaxis is known to occur in approximately 2.5 to 4.7 per million recipients of mRNA COVID-19 vaccines[5], and more generally in around 1 in 1,000,000 doses of all vaccines, but can occur in response to any vaccine or medication[6].

8.2.5 Behaviour change

Participants might feel they can modify their COVID-19 risk behaviours on the assumption that they are protected once vaccinated. Participants will be extensively counselled that they should continue to follow all up to date government advice in relation to COVID-19 precautions during the trial.

8.2.6 Specific risks from vaccines

Please refer to Section 13.8.1 for full details.

8.2.7 Antibody-dependent enhancement and immunopathology

Safety concerns around the use of some viral antigens as a vaccine antigen have been raised following historical and limited reports of immunopathology and antibody dependant enhancement (ADE)

reported in vitro and post SARS-CoV challenge in mice, ferrets and non-human primates immunised with whole SARS-CoV inactivated or full-length S protein based vaccines, including a study using Modified Vaccinia Ankara as a vector [7-9]. To date, there has been one report of lung immunopathology following MERS-CoV challenge in mice immunised with an inactivated MERS-CoV candidate vaccine [8]. However, in preclinical studies of ChAdOx1 immunisation and MERS-CoV challenge, no ADE was observed in hDPP4 transgenic mice, dromedary camels or non-human primates[10, 11].

The possibility of ADE has also been evaluated in clinical and pre-clinical studies of the vaccines used in this trial. Nevertheless, this risk will not have been assessed for heterologous boost schedules. Participants will be made aware of this theoretical risk.

8.2.8 Unwanted media attention

Trial participants can be subjected to unwanted attention from the media. They will therefore be provided with access to a document outlining some suggested media guidance.

8.2.9 Reactogenicity

Preliminary data from the “Comparing COVID-19 vaccine schedule combinations” study (COMCOV) has indicated that there may be increased reactogenicity at the second dose of vaccine from heterologous 2 dose primary regimens than from homologous. It is possible that participants might experience more reactogenicity if their booster vaccine is different to the vaccine used in their 2 dose primary regime.

9 Study Design

9.1 Trial Design

A randomised, phase II UK multi-centre study to determine reactogenicity and immunogenicity of booster vaccination against ancestral and novel variants of SARS-CoV-2. Participants, laboratory and analysing statisticians will remain blind to treatment allocation.

9.1.1 Setting

Multicentre study conducted through academic and NHS clinical trials sites.

9.1.2 Trial duration

Total duration of each participant in the primary phase of Stage 1 will be 12 months from the administration of the first vaccine dose. The total duration of each participant in the External Vaccine

Trial Sub-Study will be 8 months (+/-60 days) from vaccination in the trial. The total trial period will be approximately 1 year, 6 months.

9.1.3 Study groups

The study will initially consist of several cohorts enrolled in 2 or 3 stages

9.1.4 Stage 1

The initial aim is to test several different COVID-19 vaccines which are available and have already, or are expected to be imminently granted reg 147 use by the MHRA in the UK, as annual booster doses for participants who have received a homologous 2 dose primary vaccine schedule with either BNT162b2 (Pfizer) or ChAdOx1-nCov19 (AstraZeneca/Oxford).

Stage 1 will be run across 3 groups (A-C) which will each be enrolled at different clusters of sites, with a total of 2886 participants.

Site groups A and C will have 888 participants consisting of a cohort of 444 participants who received 2 doses of BNT162b2 with the second dose at least 3 months (=>84 days) prior to enrolment and 444 participants who received 2 doses of ChAdOx1 nCov19 with the second dose at least 3 months (=>84 days) prior to enrolment.

Site group B will have 1110 participants consisting of a cohort of 555 participants who received 2 doses of BNT162b2 with the second dose at least 3 months (=>84 days) prior to enrolment and 555 participants who received 2 doses of ChAdOx1-nCov19 with the second dose at least 3 months (=>84 days) prior to enrolment.

Each of the 3 groups in Stage 1 will be enrolled at separate clusters of 6 trial sites to reduce the risk of medication/dosing errors.

Group A: 888 participants (including 444 vaccinated previously with 2 doses of BNT162b2 and 444 vaccinated previously with 2 doses of ChAdOx1-nCov19) will be randomised 1:1:1:1 to receive a booster dose of either ChAdOx1-nCov19, NVX–CoV2373 (Novavax), NVX-CoV2373 half dose, or MenACWY (control group).

Group B: 1110 participants (including 555 vaccinated previously with 2 doses of BNT162b2 and 555 vaccinated previously with 2 doses of ChAdOx1-nCov19) will be randomised 1:1:1:1:1 to receive a booster dose of either BNT162b2, VLA2001 (Valneva), VLA2001 half dose, Ad26.COV2.S (Janssen) or MenACWY (control group).

Group C: 888 participants (including 444 vaccinated previously with 2 doses of BNT162b2 and 444 vaccinated previously with 2 doses of ChAdOx1-nCov19) will be randomised 1:1:1:1 to receive a booster dose of either mRNA-1273 (Moderna), CVnCoV (Curevac), BNT162b2 half dose, or MenACWY (control group).

All participants will be monitored for adverse events and have bloods for immunogenicity taken at days 28, and 84. All participants except for those that received VLA and CVnCoV will have bloods for immunogenicity taken at day 242. This is because these participants will have received a licensed booster outside of the trial following mass unblinding. The primary outcome assessed from stage 1 will be a geometric mean ratio in day 28 anti-spike protein IgG of 1.75 compared to the control group. In addition, an immunology subgroup comprising approximately 25 participants of each vaccine group will attend on day 7 and day 14 for immunology bloods. Half doses of 3 vaccines have been included at the request of the UK Chief Medical Office and Vaccine Task Force to investigate as potential dose sparing regimen for use in the UK or globally due to potential shortage of vaccine supply. Total target enrolment figures and the target number of participants per arm are approximates, some minor variation is expected as a result of randomisation stratification.

For procedures in the event of NHS deployment of COVID-19 booster vaccines, see section 9.1.10.

9.1.5 Stage 2 (optional group, not funded at time of stage 1 submissions and set up)

This stage is adaptive/optional and is available to utilise any additional vaccines which become available to the trial after commencement of stage 1. The design of stage 2 will mimic stage 1. If an additional vaccine is available (or if one of the vaccines planned for inclusion for stage 1 is unavailable at the time it commences and is deferred to stage 2), a further 444 participants will be enrolled (including 222 vaccinated previously with BNT162b2 and 222 vaccinated previously with ChAdOx1-nCov19). They will be randomised 1:1 to receive Vaccine C or Men ACWY as a booster. Participants will be monitored for adverse events and have bloods for immunogenicity taken at days 28, 84 and 365. In addition, an immunology subgroup comprising approximately 25 participants of each vaccine group will attend on day 7 and day 14 for immunology bloods. The primary outcome assessed from stage 2 will be a geometric mean ratio in day 28 anti-spike protein IgG of 1.75 compared to the control group. If/when further vaccines become available, a planned amendment will be added to include additional groups of 222 participants (111 who have previously received 2 doses of BNT162b2 and 111 who have had 2 doses of ChAodOx1-nCov19). Total target enrolment figures and the target number of participants per arm are approximates, some minor variation is expected as a result of randomisation stratification.

9.1.6 Stage 3

Please see Appendix G for details of the Omicron variant booster sub-study.

Please see appendix G for details of the omicron variant vaccine sub-study

9.1.7 Stage 1 Group allocations

Group	2 dose Primary	Subgroup	Booster	Visits
**Group A (n=888)**	ChAdOx1-nCov19 (n=444)	ChAd-ChAd (n=111)	ChAdOx1-nCov19	Day 0, 7* 14*, 28, 84, 242
		ChAd- NVX (n=111)	NVX–CoV2373	
		ChAd- NVX50 (n=111)	NVX–CoV2373 Half Dose^{+}	
		ChAd-Men (n=111)	MenACWY	
	BNT162b2 (n=444)	BNT-ChAd (n=111)	ChAdOx1-nCov19	Day 0, 7* 14*, 28, 84, 242
		BNT-NVX (n=111)	NVX–CoV2373	
		BNT-NVX50 (n=111)	NVX–CoV2373 Half Dose^{+}	
		BNT-Men (n=111)	Men ACWY	
**Group B (n=1110)**	ChAdOx1-nCov19 (n=555)	ChAd-BNT (n=111)	BNT162b2	Day 0, 7* 14*, 28, 84, 242
		ChAd-VLA (n=111)	VLA2001	
		ChAd-VLA50 (n=111)	VLA2001 Half Dose^{+}	
		ChAd-Jan (n==111)	Ad26.COV2.S	
		ChAd-Men (n=111)	Men ACWY	
	BNT162b2 (n=555)	BNT-BNT (n=111)	BNT162b2	Day 0, 7* 14*, 28, 84, 242
		BNT-VLA (n=111)	VLA2001	
		BNT-VLA50 (n=111)	VLA2001 Half Dose^{+}	
		BNT-Jan (n=111)	Ad26.COV2.S	
		BNT-Men (n=111)	Men ACWY	

**Group C (n=888)**	ChAdOx1-nCov19 (n=444)	ChAd-Mod (n=111)	mRNA-1273	Day 0, 7* 14*, 28, 84, 242
		ChAd-CVn (n=111)	CVnCoV	
		ChAd-BNT50 (n=111)	BNT162b2 Half Dose⁺	
		ChAd-Men (n=111)	Men ACWY	
	BNT162b2 (n=444)	BNT-Mod (n=111)	mRNA-1273	Day 0, 7* 14*, 28, 84, 242
		BNT-CVn (n=111)	CVnCoV	
		BNT-BNT50 (n=111)	BNT162b2 Half Dose⁺	
		BNT-Men (n=111)	Men ACWY	

9.1.7.1 Stage 1 Unblinded control group allocations

**Groups**	**2 dose primary**	**Subgroup**	**Booster**	**Extra visits**
Unblinded Control Groups (2 per site group, n=666)	ChAdOx1-nCov19 (n=333)	ChAd-L-BNT (n=111)	BNT162b2	UCD0, 14*, 28, 242†
		ChAd-L-BNT50 (n=111)	BNT162b2 Half dose⁺	
		ChAd-L-Mod50 (n=111)	mRNA-1273 Half dose⁺	
	BNT162b2 (n=333)	ChAd-L-BNT (n=111)	BNT162b2	UCD0, 14*, 28, 242†
		ChAd-L-BNT50 (n=111)	BNT162b2 Half dose⁺	
		ChAd-L-Mod50 (n=111)	mRNA-1273 Half dose⁺	

* Only applies to the immunology cohort comprising approximately 25 participants from each group

+ Half doses of 4 vaccines have been included at the request of the UK Chief Medical Office and Vaccine Task Force to investigate as potential dose sparing regimen for use in the UK or globally due to potential shortage of vaccine supply.

† The UCD242 visit replaces the original day 242 visit from Stage 1. Participants who subsequently join the Omicron variant sub-study will have their day 242 visit replaced by the day 0 visit for the sub-study.

9.1.7.2 Stage 1 external vaccine trial sub-study

Groups	2 dose primary	Booster	Visits
External Vaccine Trial Sub-Study (NVX-CoV2373)	NVX–CoV2373 (n=111)*	BNT162b2	D0, D14, D28, D84, D242

*Includes immunology group (n=25)

9.1.7.3 Stage 1 young adult fractional dose sub-study

**Group**	**Subgroup**	**Booster**	**Visits**
Young Adult Fractional Dosing	YA-BNT30mcg	BNT162b2 30mcg	D0, D14, D28, D84, D242
	YA-BNT10mcg	BNT162b2 10mcg	
	YA-MOD50mcg	mRNA-1273 50mcg	
	YA-MOD25mcg	mRNA-1273 25mcg	

9.1.7.4 Stage 1 fourth dose booster sub-study

**Group**	**Subgroup**	**Booster**	**Visits**
Fourth Dose Booster	4D-BNT	BNT162b2	D0, D14, D84*
	4D-MOD	mRNA-1273	

*D84 replaces the previously scheduled D242 for the main phase of the trial if the participant takes part in the fourth dose booster sub-study.

9.1.8 Stage 2 Group allocations

2 dose primary	Subgroup	Booster	Visits
ChAdOx1-nCov19 (n=222)	Ox2A (n=111)	Men ACWY	Day 0, 7*, 14*, 28, 84, 365
	Ox2B (n=111)	Vaccine C	
BNT162b2 (n=222)	Bion2A (n=111)	Men ACWY	Day 0, 7*, 14*, 28, 84, 365
	Bion2B (n=111)	Vaccine C	

* Only applies to the immunology cohort comprising approximately 25 participants from each group

9.1.9 Stage 3 Group allocations

Please see Appendix G for details of the omicron variant vaccine sub-study

9.1.10 Routine NHS deployment of booster vaccination

If a 3rd dose boost vaccine becomes recommended in the UK, we will unblind participants who are in the control arms so that they can receive a 3rd dose of COVID-19 vaccine (no sooner than their day 84 visit) via the study.

As the national booster campaign has been announced by the JCVI and Deputy Chief Medical Officer, all participants will be sent a letter which will either unblind them to allocation to the control arm, or to inform them they have received an active COVID-19 booster vaccine. If they were in the control arm, they will be invited to attend for an additional study visit (UCD0) where they will have baseline bloods taken for safety (FBC and biochem) and immunology (as per Day 0 visit). They will subsequently be randomised to receive a booster vaccination with either BNT162b2, a half dose of BNT162b2, or a half dose of mRNA-1273. Participants will attend follow up visits at day 14 (UCD14) for immunology bloods and for troponin, to monitor for signs of myo/pericarditis on the request of the JCVI and the Public Health England Immunisation Lead. Participants will then attend on day 28 (UCD28) post vaccine for additional immunology bloods. Participants in the Immunology cohort will have additional immunology bloods (see Appendix D).They will attend a UCD242 visit (8 months after receiving an active vaccine after being unblinded) which will replace their original Day 242 visit from Stage 1 unless they take part in the omicron variant vaccine sub-study, (please see appendix G). If they later participate in the Omicron variant booster sub-study, their day 242 visit will be replaced by the day 0 visit for this sub-study. Participants who received a vaccine via the NHS rather than the trial can have their day 242 visit (UCD242) calculated based on the date of the last attendance at their local site for a UCD0. Participants who do not received a 3rd dose booster vaccine by the time of booking of day 242 visits (either as part of the trial or via the NHS) can be withdrawn from the study. Following the

announcement of a Spring 2022 booster campaign for those aged over 75 and those that are considered clinically vulnerable, the window for the D242 visit for participants that previously received control vaccine has been amended to 6-9 months post booster vaccination to enable final follow up visits to be performed prior to participants receiving a fourth dose via the NHS vaccination program.

Participants will be asked to complete electronic symptom diaries for the 7 days following their booster vaccine for solicited and unsolicited reactions, similar to after their initial D0 visit. They will be asked to enter unsolicited AEs for up to 28 days following vaccination and medically attended AEs for up to 84 days following vaccination.

Participants who have received an active COVID vaccine as 3rd dose in this trial will only be unblinded to receive an additional vaccine if advised by the DMSB, and their schedule of visits will otherwise be unchanged.

9.1.10.1 Eligibility for Unblinded Control Vaccination

Participants in the unblinded control group will be eligible for booster vaccination at the UCD0 visit unless they have had a previous severe adverse reaction to mRNA vaccines or have acquired an additional COVID-19 vaccine outside of the study since enrolling. Other medical criteria will be checked prior to immunisation (including diagnosis of cancer, autoimmune conditions, neurological conditions, blood clotting conditions and pregnancy), but will not be considered a contraindication to vaccination unless the investigator feels there is a specific clinical reason to withhold vaccination for the safety of the participant.

Temporary delay criteria will also be checked including administration of other vaccines within 30 days (or 7 days for influenza vaccines), diagnosis of COVID-19 within 28 days, or current moderate/severe respiratory illness or fever. These are not absolute contraindications to vaccination on the day, and in the presence of these conditions an investigator can decide it is safe to proceed with vaccination if delaying is not possible.

9.1.11 External Vaccine trial participants Sub-Study

Participants in clinical trials for the currently unauthorised vaccine NVXCoV2373 are eligible to receive further vaccinations with authorised COVID-19 vaccines via the NHS in order to qualify for international travel. This sub-study has been set up to generate additional data on the safety, reactogenicity and immunogenicity of receiving a single dose of mRNA vaccine following 2 previous doses of NVXCoV2373.

Participants will be invited to attend a day 0 visit in the same manner as participants in stage 1 of the main Cov-Boost study for screening and vaccination with a single full dose of BNT162b2, with follow up visits at day 14, 28, 84 and then at 6 – 10 months post vaccination (day 242 +/- 60 days). Electronic diaries will be completed in the same manner as the main study.

Participants in this sub-stage of the study will receive one dose from the study team but may choose to receive a second dose via the NHS. Information on receipt of a second dose outside of the trial will be recorded by the study team.

9.1.12 Young Adult Fractional Dosing Sub-Study

Following analysis of the results of stage one of the COV-BOOST study, a half dose of BNT162b2 was found to have comparable immunogenicity to a full dose of BNT162b2, with a similar and acceptable side effect profile. A half dose of mRNA1273 has been approved and deployed as a 3rd dose booster by the NHS. As young people generally have a more robust immune response and experience more side effects from vaccination than elderly people, the Coalition for Epidemic Preparedness Innovations (CEPI) have requested a sub-study of fractional doses of mRNA vaccines given to young adults (aged 18 – 30). Dose sparing regimes also allow the same amount of vaccine supply to be distributed to more people.

Please refer to Appendix E for full details of this sub-study.

9.1.13 Fourth Dose Booster Sub-Study

Following mass deployment of 3rd dose COVID-19 booster vaccination by the NHS, and the emergence of the Omicron variant which has substantial mutations to the spike protein thought to confer a significant degree of immune escape, the question has arisen over the ability of further doses of COVID-19 vaccination to overcome this immune escape in people 30 years old or above.

Please refer to Appendix F for full details of this sub-study

9.1.14 Omicron Specific Fourth Dose Booster sub-study

The Omicron Variant of Concern (VOC) exhibits an unusually large number of mutations, including to the spike protein against which all licensed vaccines are designed. There are early epidemiological signs that this has conferred a significant degree of immune escape, predominantly against neutralisation from infection (some degree of protection against severe disease and death is expected to be preserved). As a result, there have been new vaccines designed which are specifically targeted to the spike protein of the Omicron VOC, including one based on the original mRNA-1273 vaccine.

Please refer to Appendix G for full details of this sub-study.

10 Study participants

10.1 Trial Participants

Adults aged 30 years or older who have received a complete homologous 2 dose primary course of COVID-19 vaccination with their first dose of administered in December 2020, January or February 2021.

Recruitment will be from participants aged 30 years and over (including people 75 years and older). Many health care workers will have received BNT162b2 and many adults aged 75 years and older will have received ChAdOx1-nCov19, so recruitment of health care workers who have received ChAdOx1-nCov19 and adults aged 75 years and older who have received BNT162b2 will be particularly encouraged.

Comorbidities of clinical definition mild/moderate/well-controlled will be permitted. Individuals of all ethnicities will be recruited, with recruitment of those identifying as Black and Minority Ethnic particularly encouraged.

The below criteria is inclusion / exclusion criteria for the main study. For inclusion/exclusion criteria of participants taking part in the external vaccine trial sub-study, please see section 10.4.

10.2 Inclusion Criteria

- Participant is willing and able to give written informed consent for participation in the trial.
- Male or Female, aged 30 years or above and in good health as determined by a trial clinician. Participants may have well controlled or mild-moderate comorbidity.
- Female participants of childbearing potential must be willing to ensure that they or their partner use effective contraception from 1 month prior to first immunisation continuously until 3 months after boost immunisation. See Section "Contraception and Pregnancy" for definition of child-bearing potential and definition of effective contraception.
- In the Investigator's opinion, is able and willing to comply with all trial requirements.
- Willing to allow their General Practitioner and consultant, if appropriate, to be notified of participation in the trial
- Willing to allow investigators to discuss the volunteer's medical history with their General Practitioner and access all medical records when relevant to study procedures.
- Agreement to refrain from blood donation during the study.
- Received priming dose of COVID-19 vaccination in December 2020, January or February 2021 and is at least 84 days post second vaccination. Due to the NHS deployment timelines, some sites may need to invite people who have been prime-boosted with their second dose of

AstraZeneca with a minimum of 70 days from their second dose. Sites need Sponsor approval for this prior to enrolment of people with a 70-83 day gap since their second dose in any study arm

10.3 Exclusion Criteria

The participant may not enter the trial if ANY of the following apply:

- Receipt of any vaccine (licensed or investigational) other than the study intervention within 30 days before and after each study vaccination (one week for licensed seasonal influenza vaccine or pneumococcal vaccine)
- Prior or planned receipt of any other investigational or licensed vaccine or product likely to impact on interpretation of the trial data (e.g. adenovirus vectored vaccines, any coronavirus vaccines)
- Participants who are pregnant at enrolment or planning to become pregnant during the first 3 months following vaccination.
- Administration of immunoglobulins and/or any blood products within the three months preceding the planned administration of the vaccines
- Any confirmed or suspected immunosuppressive or immunodeficient state; asplenia; recurrent severe infections and use of immunosuppressant medication within the past 6 months, except topical steroids or short-term oral steroids (course lasting ≤14 days)
- History of allergic disease or reactions likely to be exacerbated by any component of study vaccines (e.g. hypersensitivity to the active substance or any of the SmPC-listed ingredients of the Pfizer vaccine)
- Any history of anaphylaxis
- Current diagnosis of or treatment for cancer (except basal cell carcinoma of the skin and cervical carcinoma in situ)
- Bleeding disorder (e.g. factor deficiency, coagulopathy or platelet disorder), or prior history of significant bleeding or bruising following IM injections or venepuncture
- Continuous use of anticoagulants, such as coumarins and related anticoagulants (i.e. warfarin) or novel oral anticoagulants (i.e. apixaban, rivaroxaban, dabigatran and edoxaban)
- History of cerebral venous sinus thrombosis, antiphospholipid syndrome or heparin induced thrombocytopenia and thrombosis (HITT or HIT type 2)
- Suspected or known current alcohol or drug dependency

- Any other significant disease, disorder or finding which may significantly increase the risk to the volunteer because of participation in the study, affect the ability of the volunteer to participate in the study or impair interpretation of the study data
- Severe and/or uncontrolled cardiovascular disease, respiratory disease, gastrointestinal disease, liver disease, renal disease, endocrine disorder and neurological illness (mild/moderate well controlled comorbidities are allowed)
- History of active or previous auto-immune neurological disorders (e.g. multiple sclerosis, Guillain-Barre syndrome, transverse myelitis). Bell's palsy will not be an exclusion criterion
- Significant renal or hepatic impairment
- Scheduled elective surgery during the trial
- Participant with life expectancy of less than 6 months
- Participants who have participated in another research trial involving an investigational product in the past 12 weeks. This does not exclude participants in trials of AZD1222 (ChAdOx1 nCOV-19) who were originally recipients of placebo and who received AZD1222 (ChAdOx1 nCOV-19) or BNT162b2 as part of the "national schedule" with AZD1222 (ChAdOx1 nCOV-19) or BNT162b2 dose 1 from mid-Dec 2020 through end February 2021 and then AZD1222 (ChAdOx1 nCOV-19) or BNT162b2 second dose 12 twelve weeks later (this is allowed by the COV001 and COV002 protocols)
- Insufficient level of English language to undertake all study requirements in opinion of the Investigators except where translation has been able to be provided and is available.

10.3.1 Temporary exclusion criteria

If at Visit 1 Screening & Vaccination the volunteer has any of the following, they will not be enrolled that day.

- Acute respiratory illness (moderate or severe illness with or without fever)

- Fever (oral temperature greater than 37.8°C)

They may be considered for enrolment later in the trial; if they recover in sufficient time.

10.4 Eligibility for External Vaccine Trial Participants Sub-Study

Any participant from the phase 3 study of this vaccine who have not yet received any additional COVID-19 vaccines external to the trial will be eligible providing that they meet the following eligibility criteria:

Inclusion:

- Participant is willing and able to give written informed consent for participation in the trial.
- Male or female aged 30 years or above
- In the Investigator's opinion, is able and willing to comply with all trial requirements.
- Willing to allow their General Practitioner and consultant, if appropriate, to be notified of participation in the trial
- Willing to allow investigators to discuss the volunteer's medical history with their General Practitioner and access all medical records when relevant to study procedures.
- Primary immunization course (2 doses) of NVXCoV2373 COVID-19 vaccine
- Participant plans to receive 1 or 2 doses of licensed vaccine (1 dose will be received as part of this trial, the second dose will not be provided by the trial but the participant may receive a second dose via the NHS if they wish to).

Exclusion:

- Prior receipt of a third dose of COVID-19 vaccine
- History of allergic disease or reactions likely to be exacerbated by any component of the study vaccine (e.g., hypersensitivity to the active substance or any of the SmPC-listed ingredients of the Pfizer vaccine)
- Current diagnosis of or treatment for cancer (except basal cell carcinoma of the skin and cervical carcinoma in situ)
- Any confirmed or suspected immunosuppressive or immunodeficient state; asplenia; recurrent severe infections and use of immunosuppressant medication within the past 6 months, except topical steroids or short-term oral steroids (course lasting ≤14 days)
- Participant with life expectancy of less than 6 months
- Any other significant disease, disorder or finding which in the opinion of the investigator may significantly increase the risk to the volunteer because of participation in the study, affect the ability of the volunteer to participate in the study or impair interpretation of the study data
- Insufficient level of English language to undertake all study requirements in opinion of the Investigators except where translation has been able to be provided and is available.

Other medical criteria will be checked prior to immunisation (including neurological conditions, blood clotting conditions and pregnancy), but will not be considered a contraindication to vaccination unless the investigator feels there is a specific clinical reason to withhold vaccination for the safety of the participant.

Temporary delay criteria will also be checked including administration of other vaccines within 30 days (or 7 days for influenza vaccines), diagnosis of COVID-19 within 28 days, or current moderate/severe respiratory illness or fever. These are not absolute contraindications to vaccination on the day, and in the presence of these conditions an investigator can decide it is safe to proceed with vaccination if delaying is not possible.

11 Trial procedures

See table in appendix A

11.1 Recruitment

11.1.1 Volunteer Identification

Healthy volunteers aged 30 years or older will be recruited through various media. Care will be taken not to recruit from vulnerable groups (mental health or other capacity issues). This will be checked during the screening process.

Volunteers may be recruited by use of an advertisement +/- registration form formally approved by the ethics committee and distributed or posted in the following places:

- On institutional websites where information will be given and the volunteer information sheet will be downloadable or sent to the volunteer upon request
- In public places, including buses and trains, university campus, student bars, halls of residence, health centres etc. with the agreement of the owner / proprietor
- In newspapers or other literature for circulation
- On radio via announcements
- On a website operated by our group or with the agreement of the owner or operator
- As a post on a Twitter, Facebook or other social media account owned and operated by the study research groups
- By email distribution to a group or list only with the express agreement of the network administrator or with equivalent authorisation
- By email distribution to individuals who have already expressed an interest in taking part in any clinical trial at the databases held at study sites.

- On stalls or stands at exhibitions or fairs
- Via presentations (e.g. presentations at lectures or invited seminars)
- Direct mail-out: This will involve obtaining names and addresses of adults via the most recent Electoral Roll. The contact details of individuals who have indicated that they do not wish to receive postal mail-shots would be removed prior to the investigators being given this information. The company providing this service is registered under the Data Protection Act 2018. Investigators would not be given dates of birth or ages of individuals but the list supplied would only contain names of those aged 30 years or older (as per the inclusion criteria).
- NIHR National Vaccine Registry mail-out
- Direct SMS/text message using the NHS vaccine registers
- Via PIC sites and other NHS databases local to sites of those that have received Pfizer and/or AstraZeneca COVID-19 vaccine at the required times for enrolment into the study.
- Volunteers taking part in the external vaccine trial sub-study will be invited to take part by their local external vaccine trial study team. In some cases the COV-Boost study team and external vaccine trial study team will be the same team. However, on occasions where this is not the case, the invite to take part in the study (and participant information sheet if sent at the same time) must be sent by the external vaccine trial team with contact details provided for the COV-Boost study team should the volunteer wish to receive more information or take part.

Volunteers will be sent a copy of the participant information sheet in response to requests for further information. Volunteers will be given a minimum of 24 hours to review the documentation where possible, prior to attending a screening visit. Sufficient time should be given prior to taking informed consent.

11.2 Screening and Eligibility Assessment

11.2.1 Initial screening – Main Trial Only

Once participants express an interest in joining the trial, they will be directed to a 2 stage online screening process, hosted by the University of Oxford. The first stage will assess for obvious exclusion criteria. If they pass this stage they will be asked to indicate their electronic consent to cover:

1) Reporting their medical history (stage 2)
2) Telephone screening visits to review their medical history (if required). Requirement to be determined by review of responses to Part 2 of online questionnaire)

3) Permission to contact the participant's GP for further clarification of past medical history, should this be clinically indicated

Participants without a past medical history or drug history that requires further review may be invited directly to enrolment/vaccination visits.

11.2.2 Telephone screening visit(s) – Main Trial Only

Participants for whom further clarification of eligibility is required, may be invited for telephone screening visit(s), which would then be completed by member(s) of the clinical team, based on the assessment of the part 2 responses. This will be recorded in a screening CRF. This will reduce the amount of time participants have with the clinical team during their screening procedures, should they progress to Visit 1.

We may also contact the subject's general practitioner with the permission of the volunteer. GPs will be notified at the time of enrolment (vaccination) that the subject is taking part in the study.

The interval between the last screening process (whether on-line or by telephone screening) and V1 may be up to a maximum of 120 days. Volunteers will be asked to contact the study team in the interim if there are significant changes to their health status during this time

11.2.3 Screening during Visit 1

The final eligibility assessment and D0 vaccination visit will be combined into Visit 1 (V1). See Section 11.6

11.2.4 Screening: External Vaccine Trial Sub-Study

Informed consent and all screening procedures for participants taking part in the external vaccine trial sub-study will be completed on Day 0 (visit 1).

11.3 Informed Consent

The participant will personally sign and date the latest approved version of the Informed Consent form. A written version and verbal explanation of the Study Information leaflet and Informed Consent will be presented to the participant of the participant detailing:

- The exact nature of the study
- What it will involve for the participant
- The implications and constraints of the protocol
- The known side effects and any risks involved in taking part

- The sample handling protocol – participants will be informed that anonymised samples taken during the study may be shared with study collaborators
- That individual results will not be shared with participants, with the exception of their enrolment COVID-19 antibody test. This would be done at the end of the study, if requested by the participant

The Study Information leaflet will be made available to the participant for an appropriate amount of time (where possible this will be a minimum of 24 hours) prior to consent being obtained. Participants will have the opportunity to individually question an appropriately trained and delegated researcher before signing consent.

The following general principles will be emphasised:

- Participation in the study is entirely voluntary
- Refusal to participate involves no penalty or loss of medical benefits
- The participant may withdraw from the study at any time
- The participant is free to ask questions at any time to allow him or her to understand the purpose of the study and the procedures involved
- That participants will not be sure whether they have received an effective COVID-19 booster vaccination, and whether they may require a further booster vaccination in the future (main study only, this is not applicable for the External Vaccine Trial Sub-Study).
- Participants, like the general population, will not be exempt from following the contemporaneous government COVID-19 guidance to minimise viral transmission
- Samples taken as part of the study may be sent outside of the UK and Europe to laboratories in collaboration with University Hospital Southampton, Public Health England and NISEC collaborating centres. These will be de-identified. Volunteers will be asked if they consent to indefinite storage of any leftover samples for use in other ethically approved research, this will be optional

The participant will be allowed as much time as they wish to consider the information, and the opportunity to question the Investigator, their GP or other independent parties to decide whether they will participate in the study. Written informed consent will then be obtained by means of the participant dated signature, and dated signature of the person who presented and obtained the informed consent. The person who obtained the consent must be suitably qualified and experienced, and have been authorised to do so by the Chief/Principal Investigator and listed on the delegation log.

A copy of the signed informed consent will be given to the participant. The original signed form will be retained at the research study site, in the CRF.

Updated information that requires participants to be re-consented will be sent to participants and written re-consent requested at the earliest scheduled visit. If the earliest visit to occur is in the COVID-19 Pathway (C-19P), the participant may re-consent using an electronic signature for infection control purposes. Where appropriate, and when re-consenting in person is not possible (e.g. participants in self-isolation), participants may be contacted over the phone and an appropriately trained and delegated researcher will obtain re-consent. In this instance the participant will sign the form (electronic or paper) and a copy will be signed by the researcher. Re-consent via telephone at the D242 visit will be confirmed verbally, with the discussion documented by the research team and entered on the eCRF. The dates of signature may be different, and a copy containing both signatures will be provided to the participant at the next scheduled visit. As the protocol for stage 2 is adaptive, if additional groups are enrolled and stage 2 goes forward participants from stage 1 will not need to be reconsented.

11.4 Randomisation

Computer generated randomisation list will be prepared by the study statistician. In Stage 1 Participants in groups A and C will each be randomised 1:1:1:1 within the 2 cohorts (ChaAdOx1 nCOV-19 and BNT162b2 homologous boost), to the 4 available vaccines in each group using block randomisation. Participants in group B will each be randomised 1:1:1:1:1 within the 2 cohorts (ChaAdOx1 nCOV-19 and BNT162b2 homologous boost), to the 5 available vaccines in each group using block randomisation. Random block sizes of 4 or 8 will be used in group A and C, and random block sizes of 5 or 10 will be used in group B. The randomisation will be stratified by the study sites and by age (<70 and >=70). Although we are not aiming for a formal balance in recruitment of participants over or under the age of 70, this will ensure the recruited population are distributed evenly across all treatment arms within each age group.

Following the announcement of an NHS vaccination campaign, the control group will be unblinded to their allocation and invited to attend for a COVID-19 booster vaccination via the study. Participants in all 3 control groups will be randomised 1:1:1 within the 2 cohorts (ChaAdOx1 nCOV-19 and BNT162b2 homologous boost) to one of the 3 available vaccines: BNT162b2, half dose BNT162b2, or half dose mRNA-1273. Random block sizes of 3 or 6 will be used. The randomisation will be stratified by study sites and by age (<70 and >=70).

Participants in the External Vaccine Trial sub-study will not be randomised. The only vaccine to be used in this part of the study is BNT162b2.

Participants in Stage 2 will be randomised 1:1 within the 2 groups (ChaAdOx1 nCOV-19 and BNT162b2 homologous boost) to either Vaccine C or Men ACWY. Random block sizes of 2 or 4 will be used. The randomisation will be stratified by the study sites. Additional groups may be available for randomisation.

In Stage 3 Participants will be randomised 1:1:1:1:1 within the 2 groups (ChaAdOx1 nCOV-19 and BNT162b2 homologous boost), to Vaccine E, Vaccine F, Vaccine G, Vaccine H or Men ACWY using block randomisation. Random block sizes of 5 or 10 will be used. The randomisation will be stratified by the study sites.

11.5 Blinding and code-breaking

Participants and laboratory staff will remain blind to treatment allocation. Statisticians were blinded during the recruitment phase and for interim analysis up until the Statistical analysis plan (SAP) was signed off. Statisticians will be blinded for future stages or sub-stages after sign off of SAP V1.0**.** Clinical staff involved in study delivery will be aware of which vaccine the participant is receiving (arm allocation); the participants themselves will remain blinded to their vaccine allocation. Vaccines will be prepared out of sight of the participant and the blind will be maintained by applying a masking tape over the vaccine syringe.

If the clinical condition of a participant necessitates unblinding of the participant, this will be undertaken according to a trial specific working instruction and group allocation sent to the attending physician. This will be done if unblinding is thought to be relevant and likely to change clinical management.

Following the announcement of a national NHS booster vaccination campaign, participants will be unblinded to their allocation to the control arm, or one of the active COVID-19 vaccines (however, the specific COVID-19 vaccine will not be revealed until the end of the study). From this point onwards participants and investigators will be unblinded to the allocation of the control group.

Participants in the External Vaccine Trial sub-study will not be blinded to vaccine allocation. The only vaccine to be used in this part of the study is BNT162b2.

11.5.1 Unblinding of all Stage 1 trial participants (protocol V7.0)

All Stage 1 trial participants (including participants that were previously in the control cohort and then received an active vaccine as part of the study) will be unblinded following the approval of protocol V7.0. This follows the earlier completion of the primary analysis. Participants that have received a vaccine that has been deselected by the DSMB or a vaccine where it is confirmed that it will not be licensed will be advised that they may obtain another booster dose (if they are eligible) outside of the

trial, as approved by UK HSA. These participants will stay in the study for the purpose of collecting safety data only.

When participants have been unblinded they and their GP will receive a letter detailing the vaccine they received in the study.

11.6 Visits

The study visits and procedures will be undertaken by one of the clinical trials team. The procedures to be included in each visit are documented in the schedule of attendances (see Appendix A for schedule of attendances). Each visit is assigned a time-point and a window period, within which the visit will be conducted. If a participant cannot attend a visit, where possible, this will be re-arranged to an in-person visit within the time window. A telephone visit may be conducted instead of the in-person visit to ascertain as much relevant information as possible if the participant is unable to attend a visit in person because of quarantine or self-isolation restrictions and the participant will be out of window if the visit is postponed.

11.6.1 Visit 1 (D0): Final eligibility check, Enrolment and Vaccination visit

11.6.1.1 Informed consent

The participant will have informed consent taken as described in Section 11.3, before proceeding to the final eligibility check Component of V1. Individually, each volunteer will have the opportunity to question an appropriately trained and delegated researcher before signing the consent.

11.6.1.2 Final Eligibility Check V1

During the final eligibility check component of Visit 1 (V1):

If written consent is obtained, the procedures indicated in the schedule of attendances will be undertaken including:

- Confirmation of medical history
- Physical examination (if required)
- Height and weight
- Blood tests including:
    - COVID-19 immunogenicity bloods
    - Baseline bloods for safety monitoring (routine haematology & biochemistry tests)
- Nasal fluid sample
- Saliva sample (where available)

- Observations (temperature, heart rate, respiratory rate, blood pressure and oxygen saturation)
- Urine pregnancy test in females of childbearing potential

The eligibility of the volunteer will be reviewed by a suitable member of the clinical team. Decisions to exclude the volunteer from enrolling in the trial or to withdraw a volunteer from the trial will be at the discretion of the Investigator. Note that the blood tests results from this visit will not ordinarily be available at the time the decision to proceed to immunisation with these vaccines is made. Instead, these blood tests will act as a baseline assessment for any subsequent derangements of laboratory measures. Abnormal clinical findings from blood tests at screening will be assessed by a medically qualified study member. Where available, these may be compared to blood test results taken prior to the trial as part of the participant's normal medical care, to ascertain if the derangement is an acute abnormality or is a chronic change. Abnormal blood tests following screening will be assessed according to site-specific laboratory adverse event grading tables. Any abnormal test result deemed clinically significant may be repeated to ensure it is not a single occurrence. If an abnormal finding is deemed to be clinically significant, the volunteer will be informed and appropriate medical care arranged with the permission of the volunteer.

As per Section "Temporary exclusion criteria": If a volunteer has an acute respiratory illness (moderate or severe illness with/without fever) or a fever (oral temperature > 37.8°C) at Visit 1 Screening, the volunteer will not be enrolled that day, but may be considered for enrolment at a later date if they recover in sufficient time. For control participants receiving a booster and for external vaccine trial sub-study participants this temporary exclusion criteria is not a direct contraindication to vaccination.

11.6.1.3 Vaccination at V1

Volunteers will be considered enrolled to the trial at the point of vaccination. All vaccines will be administered intramuscularly according to the IMP Handling Manual, applicable COV-Boost Preparation Guide and individual IMP pharmacy manual (if applicable). The participant will stay in the trial site for observation for at least 15 minutes, in case of immediate adverse events. Photographs of vaccination sites may be taken, if required (with the participants' written, informed consent) and will not include the participants' face. Photographs will be identified by date, trial code and subject's unique identifier.

11.6.1.4 Diary cards

Participants will be given an oral thermometer, tape measure and diary card (electronic, but for those who are unable to use electronic diary cards, a paper version will be made available), with instructions on use. All participants will be given the emergency 24 hour telephone number to contact the on-call

study physician if needed. Participants will be instructed on how to self-assess the severity of these AEs. There will also be space on the diary card to self-document unsolicited AEs, and whether medication was taken to relieve the symptoms. There will also be a separate e-diary to log any medically attended AEs up until 3 months post vaccination (any medical conditions for which a doctor/dentist is seen outside of routine, planned follow-up), and any serious medical illnesses or hospital visits may have occurred over the entire course of the study. Participants will be asked to report on solicited AEs for 7 days (and longer if symptoms persist at day 7, until resolution or stabilisation of symptoms) and unsolicited AEs for 28 days. Diary cards will collect information on the timing and severity of the following solicited AEs:

Table 1. Solicited AEs collected on post vaccination diary cards

**Local solicited AEs**	Pain, Redness, Warmth, Itch, Swelling, Induration
**Systemic solicited AEs**	Fever, Feverishness, Chills, Joint pains, Muscle pains, Fatigue, Headache, Malaise, Nausea, Vomiting, Diarrhoea

Post-vaccination diary cards will be reviewed at the next scheduled study visit. The local study team will receive electronic alerts for any Grade 3 or above AEs entered into diaries, which will be monitored daily. Participants may be telephoned to discuss further, should there be any clinical concerns.

Participants will also be instructed on the use of the Medically Attended Diary Card. They will be asked to record the following healthcare encounters up until 3 months post booster dose:

- GP visits that were not planned or routine
- Attendances at A&E
- Unplanned outpatient visits to hospital e.g. attending an "Ambulatory Care" unit
- Non-routine dental visits (i.e. dental emergency)

This information will be reviewed routinely only at follow up visits. The diary card will contain an instruction to contact the trial team by telephone should any encounter be a hospitalisation, or if they have concerns about their health.

Participants entering the COVID-19 pathway will also be asked to complete a diary, see section 11.6.5 below.

11.6.2 Safety review

While there will be no planned safety pause, a review of reactogenicity data will be conducted after the initial 20 (approximately) participants per arm have received their trial dose in stage 1, stage 2, and stage 3. In case that the 3 groups in stage 1 will not start recruiting at the same time, the review

will be conducted separately for the 3 groups. This will assess reactogenicity in the first 48 hours after immunisation, with data presented by 'anonymised' group to the DSMB chair. Should significant safety concerns arise at this point a full DSMB meeting will be organised.

11.6.3 Subsequent visits

Follow-up visits will take place as per the schedule of attendances described in Appendix A. Participants will be assessed for local and systemic adverse events, interim history, review of diary cards (paper or electronic) and blood fluid/nasal fluid/saliva tests at these time points as detailed in the schedule of attendances. Additional information may be collected such as days off work following the study vaccine, any days requiring extra help or support and extra medical consultations due to side effects. Blood will also be taken for immunology purposes. Observations and physical exam will be performed as and when clinically indicated.

If participants experience adverse events (laboratory or clinical), which the investigator (physician), CI and/or DSMB chair determine necessary for further close observation, the participant may be admitted to an NHS hospital for observation and further medical management under the care of the Consultant on call.

11.6.4 Participants under quarantine

Given the evolving epidemiological situation both globally and in the UK, should a participant be unable to attend any of their scheduled or unscheduled visits, a telephone consultation will be arranged to obtain core study data where possible. Participants should not attend for in-person visits if they are in their period of self-isolation/quarantine – the exception to this is the COVID-19 Pathway.

11.6.5 Participants with confirmed SARS-CoV-2 infection (COVID-19 Pathway)

Participants will be counselled at enrolment that should they receive a positive SARS-CoV-2 test (e.g. an antigen detection or nucleic acid amplification test, for example, via test and trace or occupational health services) they should contact the trial team on receipt of the positive result. Participants will be reminded of this with a weekly text/email message (participant choice), which will commence after the first vaccine dose.

This COVID-19 (C-19) pathway will apply to participants tested via symptomatic and asymptomatic pathways.

Participants enrolled in the external vaccine trial sub-study will not need to attend COVID-19 pathway visits but positive SARS-CoV-2 tests will be documented by the study team.

Once the participant has conveyed their result to the study team, and the study team confirm an appropriate test has been used (verbal discussion with participant as to how testing was obtained, confirmatory documentation will not be sought), an appointment will be arranged to review the participant at the relevant study site. At this visit blood samples for safety (FBC, Biochemistry, CRP and others if deemed clinically relevant) and immunology (PBMCs and serum for cellular and humoral immune responses) will be taken, along with a nasopharyngeal swab for storage and subsequent viral isolation. Nasal fluid and saliva (where available) for mucosal immune response will be taken for those who receive this at their regular study visits. Vital signs and other clinical data will be recorded. Participants will also be provided with a symptom diary, which they will fill in both solicited and unsolicited symptoms for at least 7 days and until symptom resolution (excepting persistent cough and anosmia/dysgeusia, as these are recognised to be able to continue for extended periods). Additional visits on this pathway may be arranged at the clinical discretion of the investigator.

Participants will only be invited to a C-19P visit if they have access to private transport and would not require assistance to attend the visit. Participants may not attend the visit using public transport or taxis.

The windows for performing this visit are as per Table 2.

Following unblinding of all Stage 1 trial participants (protocol V7.0) no participants enrolled in Stage 1 (including the external vaccine trial sub-study, young adult fractional dosing sub-study, fourth dose sub-study and Omicron specific fourth dose sub-study) need to attend in-person visits for C19 episodes or have samples collected. COVID-19 episodes (confirmed by rt-PCR, lateral flow tests or saliva LAMP) will be recorded as an AE/AESI and an initial contact with C19 eCRF completed.

Table 2. C-19P visit windows

Visit booking P0	Margin
**Within 7 days of positive test**	0 – 7 days

Participants should be screened for severity of disease on contacting the trial team with their positive result and referred to NHS care as appropriate.

Table 3. Remote risk stratification of COVID-19 infection

Severity of illness	Features	Advice and action
**Mild**	Completing full sentences	Paracetamol for fever
	No SOB (Grade 0)	Can use NSAIDs according to NHS recommendations (advise lowest dose and shortest duration possible)
	No chest tightness (Grade 0)	Regular fluids

	Able to do ADLS (Grade 0-1)	Self-isolate as per current government guidelines
	RR 12-20	Safety net re worsening symptoms:<br>- Trial doctor for advice in hours (999 in an emergency)<br>- 111 out of hours (non-emergent)
	No other red flags/concerning features from history	Paracetamol for fever
**Moderate A**	Completing full sentences	Can use NSAIDs according to NHS recommendations (advise lowest dose and shortest duration possible)
	Able to do ADLs but lethargic (Grade 1-2)	Regular fluids
	Mild chest tightness (Grade 1)	Self-isolate as per current government guidelines
	Mild SOB on exertion only (Grade 1)	Safety net re worsening symptoms:<br>- Trial doctor for advice in hours (999 in an emergency)<br>111 out of hours (non-emergent)
	RR 12-20 (if can be observed)	
	Any symptoms from other systems considered to be moderate and not requiring medical review	
	No other red flag features from history	
**Moderate B**	Completing full sentences	For medical review<br>- Trial doctor to arrange medical review with a non-trial medical practitioner e.g. GP or hospital doctor (in-hours)<br>- Trial doctor to signpost to NHS services (out-of-hours)
	Able to do ADLs but lethargic (Grade 1-2)	Safety net – 999 if worsening beyond current symptoms
	Mild chest tightness (Grade 1-2)	Inform senior on-call clinician
	Mild SOB on exertion only (Grade 1)	
	RR 20-24 (if can be observed)	
	Any symptoms from other systems considered to be moderate and requiring medical review	
**Severe**	Any one of:	Urgent medical review
	Inability to complete full sentences	Advise participant to call 999
	Unable to do any ADLs/get out of bed (Grade 3)	Inform senior on-call clinician
	RR >25 if can be observed	
	Any other clinical concerns for severe disease	
**Of note, this is not an all-encompassing guide and individual clinical judgement by reviewing clinician should always be taken into account. Should the reviewing clinician have any concerns regardless of risk stratification then they can contact the appropriate senior clinician for further advice.**		

11.6.6 Admission of participants to hospital with COVID-19 infection

With the participant's consent, the study team will request access to medical notes or submit a data collection form for completion by attending clinical staff on any COVID-19 episodes resulting in

hospitalisation. Any data which are relevant to ascertainment of efficacy endpoints and disease enhancement will be collected. These are likely to include, but not limited to, information on ICU admissions, clinical parameters such as oxygen saturation, respiratory rates and vital signs, need for oxygen therapy, need for ventilatory support, imaging and blood tests results, amongst others.

11.7 Sample Handling

Please refer to Appendix D: Blood Sampling for schedule of frequency and volume of blood sampling.

11.7.1 Sample handling for trial purposes

11.7.1.1 Immunology blood tests

Immunogenicity will be assessed by a variety of immunological assays. This will include antibodies to SARS-CoV-Spike and non-Spike antigens by ELISA, ex vivo ELISpot assays for interferon gamma and flow cytometry assays, neutralising and other functional antibody assays. Other exploratory immunological assays including cytokine analysis and other antibody assays, DNA analysis of genetic polymorphisms potentially relevant to vaccine immunogenicity and gene expression studies amongst others may be performed at the discretion of the Investigators.

Collaboration with other specialist laboratories in the UK, Europe and outside of Europe for further exploratory tests may occur. This would involve the transfer of serum, plasma, PBMC and/or other study samples to these laboratories, but these would remain anonymised. The analyses and which laboratories carry these out will be specified in the laboratory analysis plan.

Subjects will be informed that there may be leftover samples of their blood (after all testing for this study is completed), and that such samples may be stored indefinitely for possible future research (exploratory immunology), including genotypic testing of genetic polymorphisms potentially relevant to vaccine immunogenicity. Subjects will be able to decide if they will permit such future use of any leftover samples. With the participants' informed consent, any leftover cells and serum/plasma will be frozen indefinitely for future analysis of COVID-19 and other coronaviruses related diseases or vaccine-related responses. If a subject elects not to permit this, all of that participants' leftover samples will be discarded at the end of the trial.

Samples that are to be stored for future research will be transferred to the National Biosample Centre.

11.7.1.2 Nasal fluid & saliva samples

An exploratory analysis of mucosal immunity will be conducted using nasal fluid collected at Day 0, 28 and 242 in the immunology cohort (n=approximately 650), using SAM-strips (synthetic absorptive matrix) and, where available, saliva samples. All participants who have been enrolled to groups who

will have SAM-strip and saliva sampling (where available) will also have SAM-strips and saliva taken at the C19P visit if they attend this visit. Analysis will be conducted initially with IgA and IgG ELISAs, with further exploratory immunology assays conducted based on results – more detail will be included in the laboratory analysis plan. The same statements regarding collaboration, storage and use of samples as for blood in Section 11.7.1.1 apply here. Participants in the immunology cohort of the external vaccine trial sub-study will not have nasal fluid and saliva samples taken.

11.7.1.3 Nasopharyngeal swabs

Participants seen in the C-19 pathway will have nasopharyngeal swabs taken (instructions on performing sampling in CSP). These swabs will be tested for presence of the SARS-Cov-2 virus centrally. This analysis is for research purposes, and will not be conducted in 'real-time', so will not be used to inform the requirements for participant self-isolation etc. Swabs, and/or samples obtained from them, will be stored for potential further analysis (e.g. whole genome sequencing of identified SARS-CoV-2).

11.7.2 Sample handling for standard of care

Urinary pregnancy testing

For female participants of child bearing potential only, urine will be tested for beta-human chorionic gonadotrophin (β-HCG) at screening and again immediately prior to booster vaccination. This will be a point of care test and no sample will be stored.

11.7.2.1 Safety monitoring blood tests

These will be processed at agreed NHS Trust laboratories, and destroyed in accordance with standard NHS processes. They will include:

- **Haematology** – Full Blood Count
- **Biochemistry** – Sodium, Potassium, Urea, Creatinine, Albumin, Liver Function Tests (ALT, ALP, Bilirubin) and if relevant C-reactive protein (CRP)

11.8 Early Discontinuation/Withdrawal of Participants

In accordance with the principles of the current revision of the Declaration of Helsinki and any other applicable regulations, a participant has the right to withdraw from the study at any time and for any reason, and is not obliged to give his or her reasons for doing so. The Investigator may withdraw the participant at any time in the interests of the participants' health and well-being. In addition, the participant may withdraw/be withdrawn for any of the following reasons:

- Administrative decision by the Investigator

- Ineligibility (either arising during the study or retrospectively, having been overlooked at screening).
- Significant protocol deviation
- Participant non-compliance with study requirements
- An AE, which requires discontinuation of the study involvement or results in inability to continue to comply with study procedures

The reason for withdrawal will be recorded in the CRF. If withdrawal is due to an AE, appropriate follow-up visits or medical care will be arranged, with the agreement of the volunteer, until the AE has resolved, stabilised or a non-trial related causality has been assigned. The DSMB or DSMB chair may recommend withdrawal of participants.

If a participant withdraws from the study, storage of samples will continue unless the participant specifically requests otherwise. Any data collected before their withdrawal will still be used in the analysis for safety and trial integrity; if the participant requests this could be de-identified following the end of the study.

In cases of subject withdrawal, long-term safety data collection, including some procedures such as safety bloods, may continue as appropriate if subjects have received one or more vaccine doses, unless they decline any further follow-up.

11.9 Definition of End of Trial

The end of the trial is the date of the last assay conducted on the last sample collected.

12 Trial Interventions

12.1 Investigational Medicinal Product(s) (IMP) Description

The marketing authorisation status of the vaccines included here is that the ChAdOx1-nCOV-19, BNT162b2, mRNA-1273 and Ad26.COV2.S vaccines are approved for use under a temporary authorisation of the supply of an unlicensed vaccine; regulation 174 of the Human Medicines Regulations 2012. For these vaccines there will not be IMP labelling for this trial where there is Regulation 174 approval and products will be used as supplied by manufacturer (as for national supply) and blinding performed as per section 11.5. This section will be revised with submission of the next substantial amendment if additional vaccines receive Regulation 174 approval during the trial period.

12.1.1 AstraZeneca COVID-19 vaccine (ChAdOx1 nCOV-19)

ChAdOx1 nCoV-19 is a recombinant replication-defective chimpanzee adenovirus expressing the SARS-CoV-2 spike (S) surface glycoprotein with a leading tissue plasminogen activator (TPA) signal sequence. S is a type I, trimeric, transmembrane protein located at the surface of the viral envelope, giving rise to spike shaped protrusions from the virion. The S proteins subunits are responsible for cellular receptor ACE-2 binding via the receptor-binding domain and fusion of virus and cell membranes, thereby mediating the entry of SARS-CoV-2 into the target cells. The S protein has an essential role in virus entry and determines tissue and cell tropism, as well as host range.

ChAdOx1 nCoV-19 expresses a codon-optimised coding sequence for Spike protein from the SARS-CoV-2 genome sequence accession MN908947. ChAd is a non-enveloped virus, and the glycoprotein antigen is not present in the vector, but is only expressed once the genetic code within the vector enters the target cells. The vector genes are also modified to render the virus replication incompetent, and to enhance immunogenicity. Once the vector is in the nucleus, mRNA encoding the spike protein is produced that then enters the cytoplasm. This then leads to translation of the target protein which act as an intracellular antigen

12.1.1.1 Dosage, scheduling and packaging

The dose of AstraZeneca COVID-19 vaccine is $5x10^{10}$vp in 0.5ml. The vaccine should be administered intramuscularly. The AstraZeneca vaccine is supplied in packs of 10 vials. Each vial contains 8 or 10 doses of vaccine, and is a colourless to slightly yellow, clear to slightly opaque liquid. Each dose is prepared by withdrawing 0.5 mL from a vial in a sterile 1 mL or equivalent syringe.

12.1.2 Pfizer BioNTech (BNT162b2)

BNT162b2 is a lipid nanoparticle-formulated, nucleoside-modified mRNA vaccine that encodes trimerised SARS-CoV-2 spike glycoprotein. BNT162b2 encodes the SARS-CoV-2 full-length spike, modified by two proline mutations to lock it in the prefusion conformation and more closely mimic the intact virus with which the elicited virus-neutralizing antibodies must interact. mRNA vaccines use the pathogen's genetic code as the vaccine; this then exploits the host cells to translate the code and then make the target spike protein. The protein then acts as an intracellular antigen to stimulate the immune response. The mRNA is then degraded within days. The vaccine RNA is formulated in lipid nanoparticles (LNPs) for more efficient delivery into cells after intramuscular injection.

12.1.2.1 Dosage, scheduling and packaging

The dose of Pfizer BioNTech COVID-19 vaccine is 30µg contained in 0.3ml of the diluted vaccine. A half dose is 15 µg contained in 0.15ml of the diluted vaccine. Each pack of the Pfizer BioNTech vaccine

contains 195 vials with 5 doses per vial (975 doses per pack). It is supplied with 0.9% sodium chloride diluent for injection plastic ampoules.

A half dose has been included at the request of the UK Chief Medical Office and Vaccine Task Force to investigate as potential dose sparing regimen for use in the UK or globally due to potential shortage of vaccine supply.

For the Young Adult Fractional Dosing sub-study, a dose of 10 µg is used. This dose has recently been authorised for emergency use by the FDA and EMA for children aged 5 to 11 years.

12.1.3 Moderna (mRNA-1273)

COVID-19 Vaccine Moderna (mRNA-1273) encodes the S-2P antigen, consisting of the SARS-CoV-2 glycoprotein with a transmembrane anchor and an intact S1–S2 cleavage site. S-2P is stabilized in its prefusion conformation by two consecutive proline substitutions at amino acid positions 986 and 987, at the top of the central helix in the S2 subunit. The lipid nanoparticle capsule is composed of four lipids, and formulated in a fixed ratio of mRNA and lipid.

12.1.3.1 Dosage, scheduling and packaging

The dose of Moderna COVID-19 vaccine is 0.10mg of mRNA in 0.5ml of the diluted vaccine. Each pack of the Moderna vaccine contains 10 vials with 10 doses per vial (100 doses per pack) and is a white to off white dispersion. Each dose is prepared by withdrawing 0.5ml from a vial in a sterile 1ml or equivalent syringe.

Moderna have applied to the US FDA and European Medicines Agency for a homologous 3rd dose to be of 0.05mg mRNA. Because of this and reactogenicity profiles seen in COV-BOOST stage 1 (publication in preparation, data seen by MHRA and JCVI) the JCVI, Deputy Chief Medical Officer for England and Head of Immunisation at Public Health England (co-Investigator MR) have recommended that half dose (0.05mg mRNA) be tested in COV-BOOST as a late boost in people previously given control vaccine.

For the Young Adult Fractional Dosing sub-study, a dose of 0.025mg is also used. Please refer to the IMP handling manual and applicable preparation guide for details on the dilution process to prepare this dose.

12.1.4 Novavax (NVXCoV2373)

Novavax, NVXCoV2373 is a nano-particle vaccine. It is constructed from the full-length wild-type (prototype Wuhan sequence) pre-fusion trimers of SARS-CoV2 spike glycoprotein. The native protein has been modified with several substitutions to limit protease cleavage and enhance thermal stability

(the putative native furin cleavage site has been modified from RRAR to QQAQ and 2 proline substitutions (positions K986P and V987P) in the HR1 domain). It has also been optimised for expression in insect (Spodoptera frugiperda) Sf9 cells. The recombinant S-protein genes are cloned into a baculovirus vector before being transferred into Sf9 cells. These cells then produce the protein which is extracted and purified and arranged into nanoparticles. It is co-formulated with a saponin-based adjuvant, Matrix-M1™.

12.1.4.1 Dosage, scheduling and packaging

The dose of NVXCoV2373 is 5 µg recombinant spike protein with 50 µg Matrix-M1 adjuvant (0.5ml). The vaccine is supplied in 10 dose vials. A half dose will be drawn up at 0.25ml per dose.

A half dose has been included at the request of the UK Chief Medical Office and Vaccine Task Force to investigate as potential dose sparing regimen for use in the UK or globally due to potential shortage of vaccine supply.

12.1.5 Valneva (VLA2001)

VLA2001 is a highly purified, whole virus, SARS-CoV-2 vaccine produced on serum-free Vero cells and inactivated with β propiolactone. The viral strain is derived from a Chinese tourist diagnosed in a hospital in Rome. VLA2001 is adjuvanted with the licensed adjuvant cytosine phosphor-guanine (CpG) 1018 in combination with Aluminium Hydroxide.

12.1.5.1 Dosage, scheduling and packaging

The dose of VLA2001 is 33 antigen units/dose of SARS-CoV-2 inactivated with β propiolactone with 0.5mg aluminium hydroxide and 1mg CpG 1018 in 0.5ml. Each dose is prepared by withdrawing 0.5ml from a vial in a sterile 1ml or equivalent syringe. The vaccine is supplied in 10 dose vials. A half dose will be drawn up at 0.25ml per dose.

A half dose has been included at the request of the UK Chief Medical Office and Vaccine Task Force to investigate as potential dose sparing regimen for use in the UK or globally due to potential shortage of vaccine supply.

12.1.6 Curevac (CVnCoV)

CVnCoV is an mRNA based SARS-CoV-2 vaccine encapsulated in lipid nanoparticles. The mRNA encodes a pre-fusion conformation stabilised version of the full-length Spike protein of the SARS-CoV-2 virus. The active substance is R9515, incorporating amino acid substitutions aimed to stabilise the encoded protein in the prefusion conformation, i.e. Lysine to Proline at position 986 and Valine to Proline at position 987.

12.1.6.1 Dosage, scheduling and packaging

The dose of CVnCoV is 12µg in 0.6ml of the diluted vaccine. The vaccine is supplied in 12 dose vials. It is supplied with 0.9% sodium chloride diluent for injection plastic ampoules.

12.1.7 Janssen (Ad26.COV2.S)

Ad26.COV2.S is a monovalent vaccine composed of a recombinant, replication-incompetent Ad26 vector, constructed to encode a membrane-bound full-length SARS-COV-2-S protein incorporating 2 amino acid changes in the S1/S2 junction that knock out the furin cleavage site, and 2 proline substitutions in the hinge region.

12.1.7.1 Dosage, scheduling and packaging.

Ad26.COV2.S will be supplied at a concentration of $1x10^{11}$ vp/mL as a suspension in single-use vials, with an extractable volume of 0.5 mL. $5x10^{10}$ vp dose level: 0.5 mL is withdrawn from one vial containing 0.69 mL $1x10^{11}$ vp/mL.

12.1.8 Control vaccine

Participants who are allocated to the control groups will receive an injection of MenACWY. One of two licensed quadrivalent protein-polysaccharide conjugate vaccine MenACWY vaccines will be used, with Nimenrix being considered first choice. Menveo should only be used in the event that a study site is unable to obtain supplies of Nimenrix.

Nimenrix (Pfizer). The licensed posology of this vaccine for those over 6 months of age is a single (0.5ml) intramuscular dose, containing 5mcg each of *Neisseria meningitidis* group A, C, W and Y polysaccharide, each conjugated to 44 mcg tetanus toxoid carrier protein.

Menveo (GlaxoSmithkline). The licensed posology of this vaccine for those 2 years of age and over is a single (0.5ml) intramuscular dose, containing

- 10 mcg meningococcal group A polysaccharide, conjugated to 16.7 to 33.3 mcg *Corynebacterium diphtheriae* CRM_{197} protein
- 5mcg meningococcal group C polysaccharide, conjugated to 7.1 to 12.5 mcg *C. diphtheriae* CRM_{197} protein
- 5mcg meningococcal group W polysaccharide, conjugated to 3.3 to 8.3 mcg *C. diphtheriae* CRM_{197} protein
- 5mcg meningococcal group Y polysaccharide, conjugated to 5.6 to 10.0 mcg *C. diphtheriae* CRM_{197} protein

Previous receipt of either vaccine (or a plain polysaccharide quadrivalent meningococccal A, C, W and Y vaccine) will not be a contraindication to receiving a further vaccine in this study.

12.1.8.1 Dosage, scheduling and packaging

Doses of the two vaccines are as described above, with a single dose administered in 0.5ml by intramuscular injection. There will be no additional labelling of these vaccines beyond their licensed packaging.

12.1.9 Blinding of IMPs

See Section "Blinding and codebreaking" for detail.

12.1.10 Storage of IMP

Vaccines will be stored in accordance with manufacturers' recommendations.

All movements of the study vaccines will be documented in accordance with existing standard operating procedure (SOP). Vaccine accountability, storage, shipment and handling will be in accordance with relevant SOPs and forms. To allow for participants to receive the vaccine in a short time period, additional clinic locations may be used. In this instance vaccines will be transported in accordance with local SOP's and approvals as required.

12.1.10.1 AstraZeneca COVID-19 vaccine (ChAdOx1 nCOV-19)

The AstraZeneca vaccine should be stored at +2ºC to +8ºC and has a shelf life of 6 months. The vaccine does not contain any preservative. After first opening the vial, it should be used within 6 hours when stored at room temperature (up to 30º C) or within 48 hours when stored in a refrigerator (2 to 8º C [36 to 46°F]). After this time, the vial must be discarded. The total cumulative storage time once opened must not exceed 48 hours.

12.1.10.2 Pfizer BioNTech (BNT162b2)

The Pfizer BioNTech vaccine should be stored at -70°C +/- 10°C and has shelf life of 6 months. Once thawed, the vaccine may be stored for 31 days at 2-8°C.

12.1.10.3 Moderna (mRNA 1273)

The COVID-19 Vaccine Moderna can be stored for 7 months at -25ºC to -15ºC. It should not be stored or transported on dry ice or below -40ºC. Once thawed it should not be re-frozen and may be stored refrigerated at 2 °C to 8 °C protected from light for up to 30 days if not used (needle-punctured). Chemical and physical stability of an unopened vial after removal from refrigerated conditions has been demonstrated for 24 hours at 8° to 25°C. Chemical and physical in-use stability has been demonstrated for 6 hours at 2 to 25 ºC after first puncture. It should not be re-frozen once thawed.

12.1.10.4 Novavax (NVXCoV2373)

SARS-CoV-2 rS and Matrix-M1 adjuvant should be stored at 2°C to 8°C and not frozen.

12.1.10.5 Valneva (VLA2001)

VLA2001 should be refrigerated at +2°C to +8°C and not frozen. The punctured vial should not exceed 6 hours independent of whether at room temperature or refrigerated.

12.1.10.6 Curevac (CVnCoV)

CVnCoV should be stored at -60°C or below. The diluted IMP can be stored for a maximum of 16 hours at 5 ± 3°C in the mixing vial, and subsequently stored in the syringe for a maximum of 2 hours at room temperature.

12.1.10.7 Janssen (Ad26.COV2.S)

Ad26.Cov2.S should be stored between -25°C and – 15°C

Once removed from the freezer, the unopened vaccine may be stored refrigerated at 2°C to 8°C, protected from light, for a single period of up to 28 days, not exceeding the printed expiry date (EXP).

Once thawed, the vaccine should not be re-frozen. The product can be stored between 2°C-8°C for a maximum of 6 hours or remain at room temperature (maximally 25°C) up to 3 hours after first puncture of the vial.

12.1.10.8 Control Vaccine

MenACWY should be refrigerated at +2°C to +8°C and not frozen.

12.1.11 Compliance with Trial Treatment

All vaccinations will be administered by the research team and recorded in the CRF. The study medication will be at no time in the possession of the participant and compliance will not, therefore, be an issue.

12.1.12 Accountability of the Trial Treatment

Accountability of the IMPs will be conducted in accordance with the IMP Handling Manual and IMP Management Plan.

12.1.13 Concomitant Medication

As set out by the exclusion criteria, volunteers may not enter the study if they have received: any vaccine other than the licensed seasonal influenza vaccine or pneumococcal vaccine in the 30 days prior to enrolment or there is planned receipt of any other vaccine within 30 days of each vaccination,

any investigational product within 30 days prior to enrolment or if receipt is planned during the study period, or if there is any use of immunosuppressant medication within 6 months prior to enrolment or if receipt is planned at any time during the study period (except topical steroids and short course of low dose steroids < 14 day). Concomitant medications taken at enrolment will be recorded, as will new medications taken within the 28 days after each immunisation. Subsequently only new medications taken in response to a medically attended adverse event up until 3 months post boost will be recorded.

12.1.14 Post-trial Treatment

If any of the booster vaccinations are deselected as 3rd dose boost candidates as a result of the 28 day immunogenicity data and the participant is eligible for a booster vaccination via the NHS, they may be offered an additional vaccine which has been demonstrated to elicit an acceptable rise in the primary immunogenicity analysis. Decisions regarding the need for a booster dose, the nature of the booster dose and mode of delivery (e.g. NHS vs study site) will be made in consultation with the DSMB and study management group and will take into account national JCVI advice.

12.2 Other Treatments (non-IMPS)

Participants will be advised that they may take paracetamol prophylactically after vaccine administration. This will be from the participants own supplies rather than supplied by the study team.

12.3 Other Interventions

There are no additional investigations other than those specified in this protocol.

13 Safety Reporting

Further information on safety event management including the sub-studies featured in Appendices E, F and G is included in the applicable Safety Management Plans.

13.1 Safety reporting window

Safety reporting for the trial will commence once the first participant is consented; and will end 12 months after the last participant has received the first dose of an IMP for SAEs and Adverse Events of Special Interest (AESI)s.

For individual participants the reporting period begins when they are consented, in person, at the V1 visit , and ends 12 months after the first dose of vaccine for SAEs and AESIs.

All adverse events (AEs) that result in a participants' withdrawal from the study will be followed up until a satisfactory resolution occurs, or until a non-study related causality is assigned (if the participant consents to this).

13.2 Adverse Event Definitions

**Adverse Event (AE)**	Any untoward medical occurrence in a participant to whom a medicinal product has been administered, including occurrences which are not necessarily caused by or related to that product.
**Adverse Reaction (AR)**	An untoward and unintended response in a participant to an investigational medicinal product which is related to any dose administered to that participant.<br>The phrase "response to an investigational medicinal product" means that a causal relationship between a trial medication and an AE is at least a reasonable possibility, i.e. the relationship cannot be ruled out.<br>All cases judged by either the reporting medically qualified professional or the Sponsor as having a reasonable suspected causal relationship to the trial medication qualify as adverse reactions.
**Adverse Events of Special Interest (AESI)**	Adverse events identified as being of particular relevance to the IMP's. These will also reported as an SAE, if meeting SAE criteria (e.g. hospitalisation)
**Serious Adverse Event (SAE)**	A serious adverse event is any untoward medical occurrence that:<br>• Results in death<br>• Is life-threatening<br>• Requires inpatient hospitalisation or prolongation of existing hospitalisation<br>• Results in persistent or significant disability/incapacity<br>• Consists of a congenital anomaly or birth defect*<br>Other 'important medical events' may also be considered a serious adverse event when, based upon appropriate medical judgement, the event may jeopardise the participant and may require medical or surgical intervention to prevent one of the outcomes listed above.

	NOTE: The term "life-threatening" in the definition of "serious" refers to an event in which the participant was at risk of death at the time of the event; it does not refer to an event which hypothetically might have caused death if it were more severe.
**Serious Adverse Reaction (SAR)**	An adverse event that is both serious and, in the opinion of the reporting Investigator, believed with reasonable probability to be due to one of the trial treatments, based on the information provided.
**Suspected Unexpected Serious Adverse Reaction (SUSAR)**	A serious adverse reaction, the nature and severity of which is not consistent with the Reference Safety Information for the medicinal product in question set out:<br>• In the case of a product with a marketing authorisation, in the approved summary of product characteristics (SmPC) for that product<br>• In the case of any other investigational medicinal product, in the approved investigator's brochure (IB) relating to the trial in question

NB: to avoid confusion or misunderstanding of the difference between the terms "serious" and "severe", the following note of clarification is provided: "Severe" is often used to describe intensity of a specific event, which may be of relatively minor medical significance. "Seriousness" is the regulatory definition supplied above.

13.3 Assessment results outside of normal parameters as AEs and SAEs

13.3.1 Clinical

Abnormal clinical findings from medical history or examination will be assessed as to their clinical significance throughout the trial. If an abnormal finding is deemed to be clinically significant, the participant will be informed and appropriate medical care arranged with the permission of the participant as per Section 14.6

13.3.2 Laboratory

Abnormal clinical findings from safety blood tests will be assessed by a (blind to treatment allocation) medically qualified study member. Laboratory AEs will be assessed using specific toxicity grading

scales adapted from the FDA Toxicity Grading Scale for Healthy Adult and Adolescent Volunteers Enrolled in Preventive Vaccine Clinical Trials (Appendix C: Toxicity grading scale for lab AEs)

Any abnormal test result deemed clinically significant may be repeated to ensure it is not a single occurrence, if deemed appropriate to do so in the medical opinion of the investigator.

If a repeated test remains clinically significant, the participant will be informed and appropriate medical care arranged as appropriate and with the permission of the volunteer.

13.4 Assessment of severity

The severity of clinical and laboratory adverse events will be assessed according to scales based on FDA toxicity grading scales for healthy adult volunteers enrolled in preventive vaccine clinical trials, listed in the Clinical Study Plan and in Table 44-Table 6 below.

Table 4. Severity grading for local adverse events

Adverse Event	Grade	Intensity
**Pain at injection site**	1	Pain that is easily tolerated
	2	Pain that interferes with daily activity
	3	Pain that prevents daily activity
	4	A&E visit or hospitalization
**Erythema at injection site***	1	2.5 - 5 cm
	2	5.1 - 10 cm
	3	>10 cm
	4	Necrosis or exfoliative dermatitis
**Induration/Swelling at injection site**	1	2.5 – 5 cm and does not interfere with activity
	2	5.1 - 10 cm or interferes with activity
	3	>10 cm or prevents daily activity
	4	Necrosis
***erythema ≤2.5cm is an expected consequence of skin puncture and will therefore not be considered an adverse event**		

Table 5. Severity grading criteria for physical observations.

Vital Signs	Grade 1 (mild)	Grade 2 (moderate)	Grade 3 (severe)	Grade 4 Potentially Life threatening
**Fever (Oral - °C)**	38.0 - 38.4	38.5 – 38.9	39.0 - 40	> 40
**Tachycardia (bpm)***	101 - 115	116 – 130	>130	A&E visit or hospitalisation for arrhythmia
**Bradycardia (bpm)****	50 – 54	45 – 49	<45	A&E visit or hospitalisation for arrhythmia
**Systolic hypertension (mmHg)**	141 - 150	151 – 155	≥155	A&E visit or hospitalization for malignant hypertension
**Diastolic hypertension (mmHg)**	91 - 95	96 – 100	>100	A&E visit or hospitalization for malignant hypertension
**Systolic hypotension (mmHg)*****	85 - 89	80 – 84	<80	A&E visit or hospitalization for hypotensive shock
**Respiratory Rate (breaths per minute)**	17 - 20	21-25	>25	Intubation
***Taken after ≥10 minutes at rest **When resting heart rate is between 60 – 100 beats per minute. Use clinical judgement when characterising bradycardia among some healthy subject populations, for example, conditioned athletes. ***Only if symptomatic (e.g. dizzy/ light-headed)**				

Table 6. Severity grading for local and systemic AEs

GRADE 0	None
**GRADE 1**	Mild: Transient or mild discomfort (< 48 hours); No interference with activity; No medical intervention/therapy required
**GRADE 2**	Moderate: Mild to moderate limitation in activity – some assistance may be needed; no or minimal medical intervention/therapy required
**GRADE 3**	Severe: Marked limitation in activity, some assistance usually required; medical intervention/therapy required.
**GRADE 4**	Potentially Life-threatening: Requires assessment in A&E or hospitalisation

13.5 Assessment of Causality

For every AE, an assessment of the relationship of the event to the administration of the vaccine will be undertaken by the local PI with CI oversight. An interpretation of the causal relationship of the intervention to the AE in question will be made, based on the type of event; the relationship of the

event to the time of vaccine administration; and the known biology of the vaccine therapy. Alternative causes of the AE, such as the natural history of pre-existing medical conditions, concomitant therapy, other risk factors and the temporal relationship of the event to vaccination will be considered and investigated. Causality assessment will take place during planned safety reviews, interim analyses (including if the study is paused by the DSMB due to safety concerns) and at the final safety analysis, except for SAEs, which should be assigned by the reporting investigator, immediately. Causality assessment will be recorded on the eCRF.

13.5.1 Causality assessment for participants who have received additional external vaccines

Following review of the initial results of Stage 1, the DSMB advised participants who received VLA2001 should receive an additional dose of approved COVID-19 vaccine to obtain a sufficient boost, which can be received external to the trial. In addition, as CVnCoV is no longer in development and will not be licensed, participants who received this vaccine have been advised that if they require evidence of a 3rd dose booster vaccine for travel purposes, that they may receive an approved COVID-19 booster vaccine external to the trial. Participants who have received any COVID-19 vaccination(s) post enrolment outside of the study will have all AE causality assessments designated as “Not related” from the day of the additional vaccine(s) onwards. This is due to the difficulty and complications of determining relatedness to the study intervention or the additional vaccines.

Table 7. Guidelines for assessing the relationship of vaccine administration to an AE.

**0**	No relationship	No temporal relationship to study product and<br>Alternate aetiology (clinical state, environmental or other interventions); and<br>Does not follow known pattern of response to study product
**1**	Unlikely	Unlikely temporal relationship to study product ***and***<br>Alternate aetiology likely (clinical state, environmental or other interventions) ***and***<br>Does not follow known typical or plausible pattern of response to study product.
**2**	Possible	Reasonable temporal relationship to study product; ***or***<br>Event not readily produced by clinical state, environmental or other interventions; ***or***<br>Similar pattern of response to that seen with other vaccines
**3**	Probable	Reasonable temporal relationship to study product; ***and***<br>Event not readily produced by clinical state, environment, or other interventions ***or***<br>Known pattern of response seen with other vaccines
**4**	Definite	Reasonable temporal relationship to study product; ***and***

Event not readily produced by clinical state, environment, or other interventions; ***and***<br>Known pattern of response seen with other vaccines

13.6 Procedures for Reporting Adverse Events

13.6.1 Solicited AEs

Participants will be asked to record local and systemic AEs for 7 days (and longer if symptoms persist at day seven, until resolution or stabilisation) following vaccination in the electronic diary (solicited AEs).

13.6.2 Unsolicited AEs

All local and systemic AEs occurring in the 28 days following vaccination observed by the Investigator or reported by the participant, whether or not attributed to study medication, will be recorded in electronic diaries or study database. All AEs that result in a participants' withdrawal from the study will be followed up until a satisfactory resolution occurs, or until a non-study related causality is assigned (if the participant consents to this) as per Section 11.8

SAEs and AESIs will be actively solicited at each study visit throughout the entire trial period.

13.6.3 Medically attended AEs

A medically attended AE, is defined as any adverse event for which the participant seeks medical attention either at hospital or from primary care. This explicitly excludes seeking medical attention solely for a SARS-CoV2 test. Participants will be asked to record any medically attended AEs on their diary cards. Medically attended AEs occurring up to 3 months post boost, will be directly solicited and reviewed at each study visit.

13.7 Reporting Procedures for Serious Adverse Events

In order to comply with current regulations on SAE reporting to regulatory authorities, the event will be documented accurately and notification deadlines respected. SAEs will be reported immediately the local Investigator/study team becomes aware of their occurrence. Copies of all reports will be forwarded for review to the Chief Investigator (as the Sponsor's representative) or delegate within 24 hours of the Investigator being aware of the suspected SAE. The DSMB will be notified of SAEs that are deemed possibly, probably or definitely related to study interventions; the chair of DSMB will be notified immediately (within 24 hours) of the CI/sponsor being aware of their occurrence. SAE/AESIs will not normally be reported immediately to the ethical committee(s) unless there is a clinically important increase in occurrence rate, an unexpected outcome, or a new event that is likely to affect

safety of trial participants, at the discretion of the Chief Investigator and/or DSMB. In addition to the expedited reporting above, the Investigator shall include all SAE/AESIs in the annual Development Safety Update Report (DSUR) report.

Grade 4 laboratory AEs should be reported as SAEs and under the category of outcome of an important medical event.

Cases falling under the Hy's Law should be reported as SAEs. A Hy's Law Case is defined by FDA Guidance for Industry "Drug-Induced Liver Injury: Premarketing Clinical Evaluation" (2009). Any study participant with an increase in Aspartate Aminotransferase (AST) or **Alanine Aminotransferase (ALT) ≥ 3x Upper Limit of Normal (ULN) together with Total Bilirubin ≥2xULN**, **where no other reason can be found to explain the combination of these abnormal results**, e.g., elevated serum alkaline phosphatase (ALP) indicating cholestasis, viral hepatitis A, B or C, another drug capable of causing the observed injury, amongst others.

13.7.1 Events exempt from immediate reporting as SAEs

Hospitalisation for a pre-existing condition, including elective procedures planned prior to study entry, which has not worsened, does not constitute a serious adverse event. A&E attendances should not routinely be reported as SAEs unless they meet the SAE definition described above.

13.8 Expectedness

13.8.1 SAEs

With the exception described below there are no expected serious adverse reactions. All SARs identified as probably, possibly or definitely related will therefore be treated as unexpected and reported as SUSARs.

13.8.1.1 AstraZeneca COVID-19 vaccine (ChAdOx1 nCOV-19)

No SAEs expected. Thromboembolic events associated with thrombocytopaenia are sufficiently rare that they will be reported as AESIs and SUSARs (see section 13.8.3).

13.8.1.2 Pfizer BioNTech (BNT162b2)

Anaphylaxis following immunisation is reported in the BNT162b2 Summary of Product Characteristics as an expected adverse event of unknown frequency. Accordingly, anaphylaxis within 24 hours of receipt of BNT162b2 will be considered an expected SAR to this vaccine. Acute peripheral facial nerve palsy and lymphadenopathy are described as rare and uncommon (respectively) adverse events following BNT162b2; should these be observed in participants receiving BNT162b2 and no other vaccine, and if they met the criteria for an SAE, these would be considered an expected SAR. If

experienced in participants receiving BNT162b2 and another COVID-19 vaccine then they should be classified as 'unexpected'.

13.8.1.3 COVID-19 Vaccine Moderna

Anaphylaxis following receipt of COVID-19 Vaccine Moderna is reported in the Regulation 174 Information for UK healthcare professionals as an expected adverse event of unknown frequency. Accordingly, anaphylaxis within 24 hours of receipt of COVID-19 Vaccine Moderna will be considered an expected SAR to this vaccine. Acute facial swelling (in those with a history of injections of dermatological filler) and peripheral facial nerve palsy are described as rare adverse events following COVID-19 Vaccine Moderna, while lymphadenopathy and injection site urticaria are reported as very common and common (respectively). Should these be observed in participants receiving COVID-19 Vaccine Moderna, and if they met the criteria for an SAE, these would be considered expected SARs.

13.8.1.4 Novavax, NVXCoV2373

No SAEs expected

13.8.1.5 Valneva, VLA2001

No SAEs expected

13.8.1.6 Curevac, CVnCoV

No SAEs expected

13.8.1.7 Janssen, Ad26.COV2.S

No SAEs expected. Thromboembolic events associated with thrombocytopaenia are sufficiently rare that they will be reported as AESIs and SUSARs (see section 13.8.3). Foreseeable Adverse Reactions

The foreseeable ARs following vaccination are as follows:

13.8.1.8 AstraZeneca COVID-19 vaccine (ChAdOx1 nCOV-19)

**Local reactions**	**Systemic reactions**		**Laboratory events**
At injection site<br>Common & expected	Common & expected<br>Mild to moderate	Uncommon & expected<br>Mild to moderate	
Tenderness	Fatigue	Abdominal pain	Transient neutropaenia from baseline is common and expected

Pain	Headache	Feeling dizzy	
Warmth	Myalgia	Decreased appetite	
Redness	Arthralgia	Enlarged lymph nodes	
Itching	Nausea or vomiting	Excessive sweating, itchy skin or rash	
Swelling/ bruising at the injection site	Malaise		
Lump at the injection site	Chills		
	Feverishness		
	Fever >38°		
	Coryza (sore throat, runny nose)		
These are expected to be less common after the second dose			

13.8.1.9 Pfizer BioNTech (BNT162b2)

Very common	***Common***	***Uncommon***	***Rare***	***Unknown***
Headache	Injection site redness	Lymphadenopathy	Acute peripheral facial paralysis	Anaphylaxis
Arthralgia	Nausea	Insomnia		Hypersensitivity
Myalgia		Pain in extremity		
Injection site pain/swelling		Malaise		
Fatigue		Injection site pruritis		
Chills				
Pyrexia				

13.8.1.10 COVID-19 Vaccine Moderna

**Very common**		**Common**	**Uncommon**	**Rare**	**Unknown**
Fever	Injection site swelling	Rash	Itching at injection site	Facial swelling – seen in recipients with history of injection of dermal fillers with 48 hours of vaccination	Anaphylaxis
Headache	Lymphadenopathy	Injection site redness		Facial paralysis	Hypersensitivity
Nausea	Fatigue	Injection site rash			
Vomiting	Chills				
Myalgia	Injection site pain				
Arthralgia					

13.8.1.11 Novavax, NVXCoV2373

(From the IB report of data from clinical trials)

- Reactogenicity is generally mild, and vaccinations were well tolerated.
- Following first vaccination, local reactogenicity is more frequent for the SARS-CoV-2 rS/Matrix-M1 adjuvant regimens than the unadjuvanted or placebo regimens.
- Tenderness and pain were the most frequent local AEs.
- Systemic reactogenicity were individually less frequent but were observed with greater frequency in the SARS-CoV-2 rS/Matrix-M1 adjuvant groups.
- Headache, fatigue, and myalgia were the most frequent systemic AEs.
- Following second vaccination, SARS-CoV-2 rS/Matrix-M1 adjuvant induced greater local and systemic reactogenicity, but the majority of reported symptoms remained at grade ≤ 1.
- Mean duration of reactogenicity events was ≤ 2 days without appreciable change in duration with second vaccination.

- Severe reactogenicity was infrequent (2 events after Dose 1 and 8 events after Dose 2), occurring more often with second vaccination and for systemic events, with placebo subjects citing similar frequencies as those receiving SARS-CoV-2rS.
- No subjects sought medical intervention or refused second vaccination because of reactogenicity.

13.8.1.12 Valneva, VLA2001

(From the IB report of data from clinical trials)

- The vast majority of AEs were assessed as mild or moderate
- Solicited injection site reactions were common, occurring in two thirds of vaccinees. The most common were tenderness and pain.
- The most frequent solicited systemic solicited AEs were headache, fatigue and muscle pain. Fever was uncommon, affecting only 2 participants.
- One AESI was observed, being chilblains. This was felt to be unrelated to the vaccine and a second dose was received without further problems.

13.8.1.13 Curevac, CVnCoV

(From the IB report of data from clinical trials)

- Majority of solicited AEs were mild and transient, generally resolving to normal within 48hrs
- Grade 3 AEs were more common in younger age participants and in females than males.
- Injection site pain is the most common solicited local AE.
- The most common solicited systemic AEs were fatigue, headache, myalgia and chills.
- The rate of grade 3 AEs increased after the second dose administration.
- An allergic reaction and a potential allergic reaction following vaccination was reported from 2 subjects.
- Very few subjects did not receive a second dose due to related AEs.

13.8.1.14 Janssen, Ad26.COV2.S

Very common (>1/10)	***Common (>1/100)***	***Uncommon (<1/100)***		***Rare (<1/1000)***
Injection site pain	Joint pain	Sneezing	Muscle weakness	Allergic reaction

Headache	Coughing	Tremor	Arm/leg pain	Thrombosis with thrombocytopaenia (<1/10,000)
Tiredness	Fever	Throat pain	Backache	
Muscle pain	Chills	Rash	Weakness	
Nausea	Redness and swelling of injection site	Sweating	Feeling generally unwell	

The following adverse events are considered adverse events of special interest.

Table 8. AESIs

**Immunologic**	Anaphylaxis	
**Neurological**	Isolated anosmia/ageusia*<br>Guillain-Barre Syndrome<br>Acute disseminated encephalomyelitis (ADEM)<br>Aseptic meningitis	Meningoencephalitis<br>Peripheral facial nerve palsy<br>Generalised convulsion<br>Myelitis
**Haematological**	Thrombosis**<br>Stroke<br>Thrombocytopaenia***<br>Eosinophilia****	Coagulation disorder (includes coagulopathy, thrombosis, thromboembolism, internal/external bleed and stroke)
**Cardiac**	Acute cardiovascular injury (includes myocarditis, pericarditis, arrhythmias, heart failure, infarction)	
**Dermatological**	Chilblain-like lesions<br>Single organ cutaneous vasculitis	Erythema multiforme<br>Alopecia
**Gastrointestinal**	Acute liver injury †† †	Appendicitis
**Respiratory**	ARDS††	
**Renal**	Acute kidney injury	
**Other**	COVID-19 disease†	SARS-CoV2 positivity on a validated test

*In the absence of COVID-19

** Excluding superficial thrombophlebitis (including line-associated)

*** G3 or above

**** This will be used as a marker of skewed Th2 responses and will be routinely monitored in participants attending the COVID-19 Pathway and follow-up visits. Only G2 and above.

† In particular, any occurrence of suspected vaccine associated enhanced disease (VAED) as defined by most recent Brighton Collaboration Case Definition (REF)

†† In the absence of an infective aetiology (including COVID-19)

††† As defined in Hy's Law (see Cases falling under the Hy's Law should be reported as SAEs. A Hy's Law Case is defined by FDA Guidance for Industry "Drug-Induced Liver Injury: Premarketing Clinical Evaluation" (2009). Any study participant with an increase in Aspartate Aminotransferase (AST) or **Alanine Aminotransferase (ALT) ≥ 3x Upper Limit of Normal (ULN) together with Total Bilirubin ≥2xULN, where no other reason can be found to explain the combination of these abnormal results**, e.g., elevated serum alkaline phosphatase (ALP) indicating cholestasis, viral hepatitis A, B or C, another drug capable of causing the observed injury, amongst others.)

AESIs should be collected and recorded in the AE reporting form in RedCap throughout the duration of this study. These should also be reported as SAEs if they fulfil the definition criteria for SAEs. All AESIs not already reported as SAEs should be included in the reports to the DSMB.

13.8.2 Thrombocytopaenic thrombosis following adenovirus vector vaccination

13.8.2.1 ChAdOx1-nCov19 (Oxford)

Serious thromboembolic events with concurrent thrombocytopenia, sometimes accompanied by bleeding, have occurred very rarely following vaccination with COVID-19 Vaccine AstraZeneca during post-authorisation use. This includes life-threatening and fatal cases presenting as venous thrombosis, including unusual sites such as cerebral venous sinus thrombosis, splanchnic vein thrombosis, as well as arterial thrombosis, combined with thrombocytopenia that can rapidly progress. Multifocal venous and arterial thromboses have been reported in serious cases. The majority of the events occurred within the first 14 days following vaccination but have also been reported after this period. Due to the rarity of these events, thromboembolic events with concurrent thrombocytopaenia will be considered an AESI and reported as a SUSAR.

13.8.2.2 Ad26.COV2.S (Janssen)

A warning about unusual blood clots with low platelets has been added to the product information for COVID-19 Vaccine Janssen and listed as a very rare side effect. This is following evidence in April 2021 from 8 reports from approximately 7 million people vaccinated in the US. All cases occurred in people under 60 years of age within 3 weeks of vaccination, the majority women. Blood clots occurred

mostly at unusual sites such as cerebral venous sinus thrombosis (CVST), and in arteries together with low levels of blood platelets and sometimes bleeding. Due to the rarity of these events, thromboembolic events with concurrent thrombocytopaenia will be considered an AESI and reported as a SUSAR. (https://www.ema.europa.eu/en/news/covid-19-vaccine-janssen-ema-finds-possible-link-very-rare-cases-unusual-blood-clots-low-blood)

13.8.3 Disease enhancement following vaccination

Severe COVID-19 disease will be defined as hospitalisation, with further grading of severity according to the WHO ordinal scale (June 2020) [12]. Cases of COVID-19 disease will be examined for the possibility of vaccine associated enhanced disease (VAED). This will be evaluated on the basis of the most recent recommendations of the Brighton Collaboration [13]. Detailed clinical parameters will be collected from medical records and aligned with agreed definitions, as they emerge. Samples will be collected for evaluation of immunological evidence of VAED. Investigations will be defined by the laboratory analysis plan.

13.9 SUSAR Reporting

Under direction of the Chief Investigator, all SUSARs will be reported by the CRO to the relevant Competent Authority and to the REC and other parties as applicable. For fatal and life-threatening SUSARs, this will be done no later than 7 calendar days after the CI, Sponsor or delegate is first aware of the reaction. Any additional relevant information will be reported within 8 calendar days of the initial report. All other SUSARs will be reported within 15 calendar days.

Principal Investigators will be informed of all SUSARs.

13.10 Development Safety Update Reports

A Development Safety Update Report (DSUR) will be prepared by the Sponsor to cover a one year reporting period in line with current UK legislation requirements and the approved safety management plan. This will be submitted to the Competent Authority, Ethics Committee and HRA within 60 calendar days of the defined Data Lock Point (DLP).

13.11 Interim reviews

The safety profile will be assessed on an on-going basis by the Investigators. The CI and relevant Investigators (as per the trial delegation log) will also review safety issues and SAEs as they arise. A review of reactogenicity data will occur after the initial 20 (approximately) participants per arm have received their trial dose, as per section “Safety review”

The DSMB will evaluate safety data every 4-8 weeks and/or as required and will review safety data accumulated when the study is fully recruited. The DSMB may also be consulted should safety concerns arise at any point.

13.12 Safety Holding Rules

There will be no formal pausing rules given the extensive safety database for all vaccines used in this study, however reactogenicity data will be reviewed as per section "Safety review".

The study can be paused upon advice of the DSMB, Chief Investigator, Study Sponsor, regulatory authority, Ethical Committee(s), for any single event or combination of multiple events which, in their professional opinion, jeopardise the safety of the participants or the reliability of the data.

13.13 Contraception and pregnancy

The Following are not applicable to the Fourth dose sub-study or Young adult fractional dosing sub-study

13.13.1 Contraception

Female participants of childbearing potential are required to use an effective form of contraception until three months after booster immunisation. A woman of childbearing potential is defined as a pre-menopausal female who is capable of becoming pregnant. Menopause can be diagnosed in a woman aged over 50 after one year of amenorrhoea (this applies only if the woman is not using hormonal contraception).

Acceptable forms of contraception for volunteers of female sex include:

- Established use of oral, injected or implanted hormonal methods of contraception
- Placement of an intrauterine device (IUD) or intrauterine system (IUS)
- Total hysterectomy
- Bilateral Tubal Occlusion
- Barrier methods of contraception (condom or occlusive cap with spermicide)
- Male sterilisation, if the vasectomised partner is the sole partner for the subject
- True abstinence, when this is in line with the preferred and usual lifestyle of the subject (Periodic abstinence and withdrawal are not acceptable methods of contraception)

13.13.2 Pregnancy

Should a participant become pregnant during the trial, no further study IMP will be administered. They will be followed up for clinical safety assessment with their ongoing consent and in addition will be

followed until pregnancy outcome is determined. We would not routinely perform venepuncture in a pregnant participant unless there is clinical need.

14 Statistics

14.1 Sample size

The primary analysis of this study will be a superiority test between the COVID-19 vaccine arm and the MenACWY arm within each of the three site groups and each of the two cohorts who received 2 doses of BNT162b2 or 2 doses of ChAdOx1 nCov19.

The below sample size calculation is based on the primary analysis of anti-spike protein IgG at D28 post vaccination. The current available data from the ongoing ChAdOx1 nCoV-19 trial suggests the standard deviation of anti-spike IgG at log scale (base 10) measured by standardised ELISA is 0.4 at D28 post 2nd dose.

The sample calculation for this trial is based on the following assumptions:

1. The minimum clinical difference to detect is 1.75 folds difference in GMC between the COVID-19 vaccine and control arms, i.e. 0.243 on log scale (base 10).
2. The standard deviation of the GMC on log scale (base 10) is 0.4 based on the current available data.

Based on the above assumptions, the study will need to recruit 83 participants in each arm of each cohort to achieve 90% of power at two-sided 1% significance level. Analysis will be performed separately for the three groups (A, B and C) each of which have their own control arm. We assume ~25% of study participants will be excluded from the primary analysis due to positive on anti-nuclesocapsid IgG at baseline or loss of follow-up. Therefore, the sample size in each arm will be expanded to approximately 111. The total sample size will be 111*4*2=888 for groups A and C and 111*5*2 for group B in stage 1. For 3 groups, the total sample size will be 888*2 + 1110*1 = 2886. The sample sizes will be 111*2*2=444 in stage 2, and up to 111*5*2=1110 in stage 3. The immunology cohort will used for exploratory analyses to generate hypothesis, and thus no formal sample size calculation was carried out for this cohort. The sample size of approximately 25 per arm was therefore chosen based on practical constraints. This means we will have around 20 seronegative participants in each arm for analysis.

Based on sample size of 111 per arm, the precisions and 95% confidence intervals of safety events are:

True safety event rate	Precision(normal approximation)	95% exact binomial CI
5%	±4.1%	1.8%-10.9%
10%	±5.6%	5.1%-17.1%
25%	±8.1%	17.3%-34.1%
50%	±9.3%	40.4%-59.6%

14.2 Description of statistical methods

Safety and Reactogenicity

The analysis population will be all randomised participants who received study vaccine. Counts and percentages of each local and systemic solicited adverse reaction from diary cards, and all unsolicited AEs and SAEs will be presented by groups of vaccine revived.

Immunogenicity

The immunogenicity data is expected to be highly skewed with censored data at the lower detection threshold. A value half the lower detection threshold will be used to impute the data, and the data will be log-transformed prior to analysis. The geometric mean concentration and associated 95% confidence interval will be summarised for each group at each time point, by computing the anti-log of the mean of the log-transformed data.

The primary endpoint is anti-spike IgG at Day 28 post vaccination. The geometric mean concentrations (GMC) of anti-spike IgG will be compared between each of the vaccine arms and the Men ACWY arm within each cohort under the hypothesis:

H_0: $GMC_{vaccine} / GMC_{control} = 1$ or $log_{10} GMC_{vaccine} - log_{10} GMC_{control} = 0$;

H_1: $GMC_{vaccine} / GMC_{control} \neq 1$ or $log_{10} GMC_{vaccine} - log_{10} GMC_{control} \neq 0$.

As all the trial participants received two doses of COVID-19 vaccine before enrolling, we would not expect a large proportion of censored data. Therefore, for each group A,B and C we will we will test the above hypothesis using a linear regression model adjusting for randomisation design variables study site and age (<70 and >=70), as well as baseline immunogenicity, duration between 1st- 2nd vaccine, and duration between 2nd to booster vaccine. Six regression model will be fitted for each of

the three groups and previous vaccine received (ChAdOx1-nCov19 or BNT162b2). Sensitivity analysis will be carried out to further adjust for other covariates, which will be pre-specified in the statistical analysis plan. If a very high proportion (20%) of censored data are observed in primary endpoint, then consideration will be made to use an alternative outcome as primary outcome, such as live neutralising antibodies against SARS-CoV-2, or pseudo neutralising antibodies.

As an exploratory analysis, we will also undertake target comparisons where the data across the three groups (A, B and C) are combined to allow comparison of different vaccine schedules, especially the homologous prime-boost schedule and heterologous prime-boost schedules. If useful, we will calculate the adjusted difference in Geometric Mean Ratio (GMR) compared to the combined control group for each vaccine schedule enabling an informal indirect comparison. We will then calculate direct comparisons for each homologous prime-boost schedule (ie ChAdOx1/ ChAdOx1/ ChAdOx1 and BNT162b2/ BNT162b2/BNT162b2) versus the relevant heterologous prime-boost schedules i.e. those involving the same primary immunisation. For these comparisons, as they are exploratory we will present the GMR with 95% confidence interval. Other targeted comparisons we be performed as required. The primary analysis will be conducted on the modified intent-to-treat basis, i.e. we will only include people who were negative on anti-nucleocapsid IgG at baseline and whose primary endpoint at D28 post vaccination is available. The statistical tests for the primary analysis will be two-sided and a p-value less than 0.01 will be considered significant. Multiple imputation will be performed as a sensitivity analysis including all participants randomised. Residual analysis will be used to check model fit. If model fit is not satisfactory specification of model covariates will be examined or alternative model will be used.

A fully detailed statistical analysis plan will be prepared and will be signed off by the Chief Investigator and Statistician prior to conducting any data analyses.

14.3 Interim analyses

The interim analysis on immunogenicity will be carried out when the primary endpoint of D28 anti-spike IgG data become available at each stage, while the interim analysis on reactogenicity will be conducted when the 7-day dairy data become available. Due to the urgency in providing evidence to MHRA/JCVI/VTF for policy making on the third dose vaccine recommendation, we presented the preliminary analysis results before the data lock for interim analysis when >95% of primary endpoint data were available. The aggregated results were only shared with the relevant parties before publishing the full interim analysis.

There will be stopping rules for the interim analyses and the analyses will not affect the continuation of trial.

14.4 Missing data

The level and pattern of the missing data in the baseline variables and outcomes will be reported. The potential causes of any missing data will be investigated and documented as far as possible. Any missing data will be dealt with using methods appropriate to the conjectured missing mechanism and level of missing.

15 Data Management

The Chief Investigator will be responsible for all data that accrues from the study. The sponsor will act as data controller, University of Oxford will act as a data processor under appropriate agreements.

15.1 Access to Data & Data Protection

Direct access will be granted to authorised representatives from the Sponsor, host institution and the regulatory authorities to permit trial-related monitoring, audits and inspections. The study protocol, documentation, data and all other information generated will be held in strict confidence. No information concerning the study or the data will be released to any unauthorised third party, without prior written approval of the sponsor.

15.2 Data Recording

All clinical study data including participant diary will be recorded directly into an EDC system (REDCap) or onto a paper source document for later entry into EDC if direct entry is not available. This includes safety and outcome data. Any additional information that needs recording, but is not relevant for the eCRF (e.g signed consent forms) will be recorded on separate paper source documents. All documents will be stored safely and securely in confidential conditions. The EDC online data is stored on University of Oxford servers.

All participant reported adverse event data (both solicited & unsolicited) will be entered onto electronic diary cards (e-diaries) for a maximum of 28 days following administration of the IMP. The eDiary provides a full audit trial of edits and will be reviewed at time-points as indicated in the schedule of events. Any adverse event continuing beyond the period of the diary will be copied into the eCRF as required for safety review.

The participants will be identified by a unique trial specific number and code in any database. The name and any other identifying detail will NOT be included in any trial data electronic file, with the exception of the electronic diaries, for which consent will be obtained to store the participant email address for quality control purposes. Only site research staff, sponsor staff, PHARMExcel monitors (as delegated by sponsor) and University of Oxford data managers have access to view the email address.

The EDC system (CRF data) uses a relational database (MySQL/ PostgreSQL) via a secure web interface with data checks applied during data entry to ensure data quality. The database includes a complete suite of features which are compliant with GCP, EU and UK regulations and Sponsor security policies, including a full audit trail, user-based privileges, and integration with the institutional LDAP server. The MySQL and PostgreSQL database and the webserver will both be housed on secure servers maintained by the University of Oxford IT personnel. The servers are in a physically secure location in Europe. Backups will be stored in accordance with the IT department schedule of daily, weekly, and monthly retained for one month, three months, and six months, respectively. The IT servers provide a stable, secure, well-maintained, and high capacity data storage environment. REDCap is a widely-used, powerful, reliable, well-supported system. Access to the study's database will be restricted to members of the study team by username and password.

15.3 Record keeping

The Investigators will maintain appropriate medical and research records for this trial, in compliance with GCP and regulatory and institutional requirements for the protection of confidentiality of volunteers. The Chief Investigator, co-Investigators and clinical research nurses will have access to records. The Investigators will permit authorised representatives of the Sponsor(s) and Host institution, as well as ethical and regulatory agencies to examine (and when required by applicable law, to copy) clinical records for the purposes of quality assurance reviews, audits and evaluation of the study safety and progress.

Identifiable information such as contact details will be stored for a minimum of 5 years from the end of the study. This includes storage of consent forms. Storage of these data will be reviewed every 5 years and files will be confidentially destroyed if storage is no longer required. Considerations at the time of this review will include the value of retaining this information for participant safety (e.g. to inform participants of unexpected safety signals emerging from post-licensing surveillance), as a resource for the participants (e.g. if they wish to check which vaccines they have received in the study) and any regulatory requirements. Financial information will be stored for 7 years. De-identified research data maybe be stored indefinitely. If volunteers consent to be contacted for future research, a record of this consent will be recorded, retained and stored securely and separately from the research data. If volunteers consent to have their samples stored and used in future research, information about their consent form will be retained and stored securely as per Biobanking procedures and SOP.

15.4 Source Data and Case Report Forms (CRFs)

All protocol-required information will be collected in CRFs designed by the Investigator. All source documents will be filed in the participant file. Source documents are original documents, data, and records from which the participant CRF data are obtained. For this study, these will include, but are not limited to, volunteer consent form, blood results, GP response letters, laboratory records, diaries, medical records and correspondence. In the majority of cases, CRF entries will be considered source data as the CRF is the site of the original recording (i.e. there is no other written or electronic record of data). In this study this will include, but is not limited to medical history, medication records, vital signs, physical examination records, urine assessments, adverse event data and details of vaccinations. All source data and participant files will be stored securely. Safety blood results source data will be available in the form of an original print out from each local site's safety lab systems. Safety blood results will be entered into the eCRF using double data entry.

To prevent withdrawal of a participant due to relocation, if there is a nearby participating site and with the consent of the participant, copies of relevant participant research records (such as ICF, paper source documents) will be transferred to the local site using secure email addresses such as nhs.net or by password protected sheets. The electronic research data stored on REDCap will also be transferred to the new site. The original records will be retained by the recruiting site.

15.5 Data Quality

Data collection tools will undergo appropriate validation to ensure that data are collected accurately and completely. Datasets provided for analysis will be subject to quality control processes to ensure analysed data is a true reflection of the source data.

Trial data will be managed in compliance with local University of Oxford data management SOPs. If additional, study specific processes are required, an approved Data Management Plan will be implemented

15.6 Data Sharing

For participants who are also registered on NHS Digital's '*Sign up to be contacted for coronavirus vaccine studies'* service, we will share the minimum amount of information necessary with NHS Digital in order to allow them to update their database so that participants are not contacted about further trials, as participants are permitted only to be in one vaccine study at a time.

Personally identifiable information will be shared with Public Health England regarding SARS-CoV2 PCR test results depending on the most up to date legal requirement to report on Notifiable Diseases at the time.

16 Quality Assurance Procedures

16.1 Risk assessment

The trial will be conducted in accordance with the current approved protocol, GCP, relevant regulations and standard operating procedures. A risk assessment and monitoring plan will be prepared before the study opens and will be reviewed as necessary over the course of the trial to reflect significant changes to the protocol or outcomes of monitoring activities.

16.2 Monitoring

Monitoring will be performed according to Good Clinical Practice (GCP) guidelines by external monitors. Following written SOPs, the monitors will verify that the clinical trial is conducted and data are generated, documented and reported in compliance with the protocol, GCP and the applicable regulatory requirements. The investigator sites will provide direct access to all trial related source data/documents and reports for the purpose of monitoring and auditing by the Sponsor or the Host institution and inspection by local and regulatory authorities

16.3 Trial committees

16.3.1 Trial Steering Committee

A Trial Steering Committee will be formed to oversee the study, and advise the Study Management Committee on key issues of study conduct, including, but not limited to, study pauses due to safety concerns on the advice of the DSMB.

16.3.2 Data Safety Monitoring Board

A Data Safety Monitoring Board (DSMB) will be convened. The DSMB will evaluate frequency of events, safety and efficacy data as specified in the DSMB charter. The DSMB will make recommendations concerning the conduct, continuation or modification of the study for safety reasons to the Trial Steering Committee.

The DSMB will review SAEs or AESIs deemed possibly, probably or definitively related to study interventions. The DSMB will be notified within 24 hours of the CI/Sponsor being aware of their occurrence. The DSMB can recommend placing the study or specific arms of the study on hold if deemed necessary following a study intervention-related SAE.

16.3.3 Study Management Group

Consists of the site Investigators, Sponsor Representatives, PHARMExcel project managers the Laboratory lead for Public Health England.

17 Protocol Deviations

A trial related deviation is a departure from the ethically approved trial protocol or other trial document or process (e.g. consent process or IMP administration) or from Good Clinical Practice (GCP) or any applicable regulatory requirements. Deviations from the protocol will be documented in a protocol deviation form according to SOP R&D/Gen/Admin/009 and filed in the trial master file.

These will be managed as per Sponsor (UHS) SOP R&D/Gen/Admin/009.

18 Serious Breaches

The Medicines for Human Use (Clinical Trials) Regulations contain a requirement for the notification of "serious breaches" to the MHRA within 7 days of the Sponsor becoming aware of the breach.

A serious breach is defined as “A breach of GCP or the trial protocol which is likely to affect to a significant degree –

(a) the safety or physical or mental integrity of the subjects of the trial; or

(b) the scientific value of the trial”.

In the event that a serious breach is suspected the Sponsor must be contacted within 1 working day. In collaboration with the CI the serious breach will be reviewed by the Sponsor and, if appropriate, the Sponsor will report it to the REC committee, Regulatory authority and the relevant NHS host organisation within seven calendar days.

19 Ethical And Regulatory Considerations

19.1 Declaration of Helsinki

The Investigator will ensure that this trial is conducted in accordance with the principles of the Declaration of Helsinki.

19.2 Guidelines for Good Clinical Practice

The Investigator will ensure that this trial is conducted in accordance with relevant regulations and with Good Clinical Practice.

19.3 Approvals

Following Sponsor approval the protocol, informed consent form, participant information sheet and any proposed advertising material will be submitted to an appropriate Research Ethics Committee (REC), HRA (where required), regulatory authorities (MHRA in the UK), and host institution(s) for

written approval. No amendments to this protocol will be made without consultation with, and agreement of, the Sponsor.

The Investigator is responsible for ensuring that changes to an approved trial, during the period for which regulatory and ethical committee(s) approval has already been given, are not initiated without regulatory and ethical committee(s)' review and approval except to eliminate apparent immediate hazards to the subject (i.e. as an Urgent Safety Measure).

19.4 Other Ethical Considerations

Study team members are not eligible for participation in the study. Family members of the study team are not barred from inclusion in the trial.

Participants who become eligible for routine booster SARS-CoV-2 immunisation as per national guidelines will not be excluded from participation in the trial; but will be counselled specifically on the risks of receiving an unapproved schedule. In particular, the risks of reduced efficacy and unforeseen safety concerns will be discussed.

19.5 Reporting

The CI shall submit once a year throughout the clinical trial, or on request, an Annual Progress Report to the REC, HRA (where required), host organisation, funder (where required) and Sponsor. In addition, an End of Trial notification and final report will be submitted to the MHRA, the REC, host organisation and Sponsor.

19.6 Transparency in Research

Prior to the recruitment of the first participant, the trial will have been registered on a publicly accessible database. Results will be uploaded to ISRCTN database within 12 months of the end of trial declaration by the CI or their delegate. Where the trial has been registered on multiple public platforms, the trial information will be kept up to date during the trial, and the CI or their delegate will upload results to all those public registries within 12 months of the end of the trial declaration.

19.7 Participant Confidentiality

The study will comply with the General Data Protection Regulation (GDPR) and Data Protection Act 2018, which require data to be de-identified as soon as it is practical to do so. The processing of personal data of participants will be minimised by making use of a unique participant study number only on all study documents and any electronic database(s), with the exception of informed consent forms, participant ID log and electronic diaries. All documents will be stored securely and only accessible by study staff and authorised personnel. The study staff will safeguard the privacy of

participants' personal data. A separate confidential file containing identifiable information will be stored in a secured location in accordance with the current data protection legislation. Photographs of vaccination sites if required (with the participants' written, informed consent), will not include the participants' face and will be identified by the date, trial code and subject's unique identifier. Once developed, photographs will be stored as confidential records, as above. This material may be shown to other professional staff, used for educational purposes, or included in a scientific publication.

19.8 Expenses and Benefits

Volunteers will be compensated for their time, the inconvenience of having blood tests and procedures, and their travel expenses. The total amount compensated will depend on the exact number of visits, and whether any repeat or additional visits are necessary. For all trial visits compensation will be calculated according to the following:

- Travel expenses: £15 per visit
- Inconvenience of blood tests: £10 per blood donation
- Time required for visit: £20 per visit

20 Finance And Insurance

20.1 Funding

The study is funded by National Institute Health Research (NIHR), supported by the Vaccine Task Force and DHSC.

Insurance

The Sponsor has a specialist insurance policy in place which would operate in the event of any participant suffering harm as a result of their involvement in the research. NHS indemnity operates in respect of the clinical treatment that is provided.

20.2 Contractual arrangements

Appropriate contractual arrangements will be put in place with all third parties.

21 Publication Policy

The Investigators will be involved in reviewing drafts of the manuscripts, abstracts, press releases and any other publications arising from the study. Data from the study may also be used as part of a thesis for a PhD or MD.

22 Development Of A New Product/ Process Or The Generation Of Intellectual Property

Ownership of IP generated by employees of the Trust vests in the Trust. The Trust will ensure appropriate arrangements are in place as regards any new IP arising from the trial.

23 Archiving

Study data may be stored electronically on a secure server, and paper notes will be kept in a key-locked filing cabinet at the site. All essential documents will be retained for a minimum of 5 years after the study has finished with 5 yearly reviews. The need to store study data for longer in relation to licensing of the vaccine will be subject to ongoing review. For effective vaccines that may be licensed, we may store research data securely at the site at least 15 years after the end of the study, subject to adjustments in clinical trials regulations. Where relevant participants' bank details will be stored for 7 years in line with the site financial policy. De-identified research data maybe be stored indefinitely, but with 5 yearly review.

General archiving procedures will be conducted in compliance to SOP R&D/Gen/Admin/022 SOP for the Archiving of Clinical Trial Data.

24 References

1. Dan, J.M., et al., *Immunological memory to SARS-CoV-2 assessed for up to 8 months after infection.* Science, 2021. **371**(6529).
2. Doria-Rose, N., et al., *Antibody Persistence through 6 Months after the Second Dose of mRNA-1273 Vaccine for Covid-19.* N Engl J Med, 2021.
3. Wu, K., et al., *Serum Neutralizing Activity Elicited by mRNA-1273 Vaccine.* N Engl J Med, 2021.
4. Madhi, S.A., et al., *Efficacy of the ChAdOx1 nCoV-19 Covid-19 Vaccine against the B.1.351 Variant.* N Engl J Med, 2021.
5. Shimabukuro, T., et al., *Reports of Anaphylaxis After Receipt of mRNA COVID-19 Vaccines in the US—December 14, 2020-January 18, 2021.* JAMA, 2021.
6. Public Health England, *Vaccine safety and adverse events following immunisation: The Green Book, Chapter 8.* 2020.
7. Weingartl, H., et al., *Immunization with modified vaccinia virus Ankara-based recombinant vaccine against severe acute respiratory syndrome is associated with enhanced hepatitis in ferrets.* J Virol, 2004. **78**(22): p. 12672-6.
8. Agrawal, A.S., et al., *Immunization with inactivated Middle East Respiratory Syndrome coronavirus vaccine leads to lung immunopathology on challenge with live virus.* Hum Vaccin Immunother, 2016. **12**(9): p. 2351-6.
9. Liu, L., et al., *Anti-spike IgG causes severe acute lung injury by skewing macrophage responses during acute SARS-CoV infection.* JCI Insight, 2019. **4**(4).
10. Munster, V.J., et al., *Protective efficacy of a novel simian adenovirus vaccine against lethal MERS-CoV challenge in a transgenic human DPP4 mouse model.* NPJ Vaccines, 2017. **2**: p. 28.

11. Alharbi, N.K., et al., *Humoral Immunogenicity and Efficacy of a Single Dose of ChAdOx1 MERS Vaccine Candidate in Dromedary Camels.* Sci Rep, 2019. **9**(1): p. 16292.
12. W. H. O. Working Group on the Clinical Characterisation Management of Covid Infection, *A minimal common outcome measure set for COVID-19 clinical research - The Lancet Infectious Diseases.* 2020.
13. Brighton Collaboration. *COVID-19 publications*. [cited 2020; Available from: https://brightoncollaboration.us/covid-19/.

25 Appendix A: Schedule of Visitation

25.1 Schedule of visits for general cohort

	Screening	V1	V2	V3	V4	(VPP) Only if enter C19P‡
**Study timeline**		D0	D28	D84	D242†	(D0-D242)
**Study window**		Within 120d of screening	-7/+3	±14	-7/+28	Within 7 days of positive test
**Informed consent**	X*	X				
**Safety bloods**		X	X			X
**Medical history**	X					
**Interim medical history**		X	X	X	X	X
**Physical examination (as required)**		(X)	(X)	(X)	(X)	X
**Urine test (Pregnancy) (if required)**		X				
**COVID-19 vaccination**		X				
**COVID-19 immunogenicity bloods**		X	X	X	X†	X
**SARS-Cov-2 viral swab**						X
**Diary card review**			X			X
**SAE/AESI/Medically attended AE check**			X	X	X	X

*Online Consent. Restricted to taking a limited medical history online, contacting the GP for further medical record if required and telephone screening visit(s)

† Following mass unblinding, participants who received VLA2001 or CVnCoV will attend a telephone visit for D242 and **no** blood samples should be collected

‡ C19 visits and samples are not required after unblinding of all Stage 1 participants

25.2 Schedule of visits for immunology cohorts

	Screening	V1	V2	V3	V4	V5	V6≠	(VPP) Only if enter C19P‡
**Study timeline**		D0	D7	D14	D28	D84	D242†	(D0-D242)
**Study window**		Within 120d of screening	±1	±2	-7/+3	±14	-7/+28	Within 7 days of positive test
**Informed consent**	X*	X						
**Safety bloods**		X	X		X			X
**Medical history**	X							
**Interim medical history**		X	X	X	X	X	X	X
**Physical examination (as required)**		(X)	(X)	(X)	(X)	(X)	(X)	X
**Urine test (Pregnancy) (if required)**		X						
**COVID-19 vaccination**		X						
**COVID-19 immunogenicity bloods**		X	X	X	X	X	X	X
**SAM strip**		X			X		X	X
**Saliva****		X			X		X	X
**SARS-Cov-2 viral swab**								X
**Diary card review**					X			X
**SAE/AESI/Medically attended AE check**			X	X	X	X	X	X

*Online Consent. Restricted to taking a limited medical history online, contacting the GP for further medical record if required and telephone screening visit(s)** Where available † Following mass unblinding, participants who received VLA2001 or CVnCoV will attend a telephone visit for D242 and no blood samples should be collected ‡ C19 visits and samples are not required after unblinding of all Stage 1 participants. ≠Participants taking part in the fourth dose sub-study will attend their final visit 3 months post receiving their fourth dose. This visit replaces the previously scheduled D242 visit. Please see section 30.6.

25.3 Schedule of additional visits for unblinded control arms in event of NHS national booster campaign

	V1	V2	V3	V4
**Study timeline**	UCD0	UCD14	UCD28	UCD242†
**Study window**	NA	±3	±7	-60/+30
**Informed consent**	X			
**Interim medical history**	X			
**Physical examination (as required)**	(X)	(X)	(X)	(X)
**Urine test (Pregnancy) (if required)**	X			
**COVID-19 vaccination**	X			
**COVID-19 immunogenicity bloods**	X	X	X	X
**Safety bloods**	X		X	
**Troponin**	X	X		
**SAM strip***	X*		X*	
**Saliva***	X*		X*	
**SAE/AESI/Medically attended AE check**	X	X	X	X

* Immunology cohort only

†For participants in the unblinded control arm who received an NHS booster vaccine and were not vaccinated at site, their UCD242 visit date can be calculated based on the date of the last attendance at the local site for a for UCD0 visit

25.4 Schedule of visits for External vaccine trials participant

	V1	V2	V3	V4	V5
**Study timeline**	D0	D14	D28	D84	D242
**Study window**	N/A	±3	-7/+3	±14	±60
**Informed consent**	X				
**Safety bloods**	X	X	X		
**Medical history**	X				
**Interim medical history**		X	X	X	X
**Physical examination (as required)**	(X)	(X)	(X)	(X)	(X)
**Urine test (Pregnancy) (if required)**	X				
**COVID-19 vaccination**	X				
**COVID-19 immunogenicity bloods**	X	X	X	X	X
**Diary card review**		X	X		
**SAE/AESI/Medically attended AE check**		X	X	X	X
**Troponin**	X	X			

26 Appendix B: Amendment History

Amendment No.	Protocol Version No.	Date issued	Author(s) of changes	Details of Changes made
1	2.0	May 14th 2020	Alasdair Munro	• Amended eligibility criteria to be minimum of 84 days post second COVID-19 vaccine, however minimum of 70 days post second vaccine of ChAdOx-nCoV19 is allowable with Sponsor approval at site.<br>• Removed confirmed SARS-CoV-2 infection as exclusion criteria<br>• Amended pregnancy/contraception as inclusion/exclusion to only be relevant for the first 3 months of the trial following vaccination<br>• Clarified diary checks to be performed at follow up visits with daily monitoring for email alerts of Grade 3 AEs<br>• Thrombocytopaenic thrombosis amended to no longer be expected SAE. Now will be considered an AESI and SUSAR<br>• Clarification that CI will be responsible for SUSAR reporting<br>• Mucosal immunity samples only to be collected at D0, 28 and 365<br>• Additional serum sample from immunology cohort at D0, 28 and 365 for functional antibody assays<br>• Removed safety bloods from D14 in the schedule of visits for the immunology

				cohort. This was an error and conflicted with appendix D<br>• Clarified that participants should have a minimum of 24 hours between receiving documents and attending a screening visit where possible. Sufficient time should be given before consent.<br>• Amended the D28 visit window to -7/+3 days
2	2.1	May 19th 2021	Stephen Saich<br>Alasdair Munro	• Removed reference to an alternative dose for Ad26.COV2.S vaccine in section 12.1.7. This was an administrative error has been removed. The table of interventions remains correct.<br>• Removed reference to a group of participants receiving two doses of Men ACWY vaccines. This was an administrative error and has been removed. The table of interventions remains correct.<br>• Adjusted blood collection volumes due to align serum sample collection volumes across groups and reduce LiHep sample volumes due to meet lab capacity constraints.<br>• Added mRNA1273 to IMP section of synopsis, previously omitted as administrative error.<br>• Clarification that saliva samples will only be taken where available<br>• Removal of tenderness from the list of solicited AEs collected due to anticipated participant difficulty in differentiating this from pain.<br>• Correction of typographical errors

3	3.0	June 1st 2021	Alasdair Munro	• The treatment arm for half dose CVnCoV (Curevac) in Group C has been changed to a half dose of BNT162b2 (Pfizer) on advice from the UK Vaccine Task Force.<br>• Lay summary and IMP section updated to reflect the reg 174 approval of the Janssen vaccine Ad26.COV2.S.<br>• Inclusion criteria amended to include volunteers who received their first dose of COVID-19 vaccine in February 2021, who are also a minimum of 84 days post boost (or 70 days post second dose of Ox/AZ if site has sponsor approval)<br>• Addition of text to support recruitment “Direct SMS/text message using the NHS vaccine registers”<br>• Updated BNT162b2 (Pfizer) storage conditions to 31 days from 5 days in line with national guidance.
	3.1	June 10th 2021	Saul Faust	• Addition of items to be included in follow up CRFs<br>• Correction of typos (2 additional instances of reference to JCVI priority groups 1-3 identified and removed).
	3.2	June 14th 2021	Alasdair Munro	• Clarification that number of participants in each immunology subgroup is approximately 25, and that the allocation of participants to each treatment arm is approximately 111

	4.0	Sept 8th 2021	Alasdair Munro	• Additional secondary endpoint added for immunological outcomes for a later booster immunization (after 7m), and in comparison to early booster (after 3m).<br>• Additional secondary endpoint added for changes in biomarkers of myocardial injury in participants having later booster immunisation via Troponin<br>• Additional section 9.1.7.1 outlining group allocations of unblinded controls randomized to a booster vaccine.<br>• Amended section 9.1.10 to clarify procedures for unblinding the control arm in event of deployment of a national NHS booster vaccine campaign and administer vaccine as part of the study.<br>• Amended section 11.4 to update randomisation plan for unblinded control group vaccination visit<br>• Amended section 11.5 to clarify that after unblinding of the control arm, statisticians and investigators will also have been unblinded to their initial allocation.<br>• Amended schedule of visitation (25.3) for additional booster vaccine visits for the unblinded control group (UCD0 – UCD28).<br>• Amended blood sampling tables (28) for additional booster vaccine visits for the unblinded control group (UCD0 – UCD28).<br>• Addition of Troponin blood test to the UCD0 and UCD14 visit.
	5.0	Oct 1st 2021	Alasdair Munro	• Clarification of eligibility criteria for vaccination of unblinded control group,

				including that pregnancy will not be an exclusion criteria or contraindication to vaccination<br>• Clarification of secondary endpoints for unblinded control group vaccination being approximately 6 months after primary COVID-19 immunisation schedule
	6.0	Oct 21st 2021	Alasdair Munro	• Addition of section on new sub-study of previous external vaccine clinical trial participants who have received NVXCoV2373 as their primary immunization course, receiving a 3rd booster dose<br>• Additional schedule of events for external vaccine clinical trial sub study participants<br>• Additional blood sampling table for external vaccine clinical trial sub study participants<br>• Addition of new secondary endpoints for safety, reactogenicity and immunogenicity of external vaccine clinical trial sub study participants<br>• Amended section 11.8 to remove legacy section which is no longer relevant since unblinding of control group<br>• Additional recruitment methods added for the external vaccine trial sub-study<br>• Addition of expected SAE for the Novavax vaccine
	6.1	Nov 12th 2021	Stephen Saich	• Removed the requirement to collect lithium heparin samples at D14 for participants in the general cohort WITH lithium heparin samples in the External Vaccine Trial Sub-Study

	7.0	Nov 30th 2021	Stephen Saich	• Addition of information on unblinding all stage 1 trial participants following approval of V7.0<br>• Addition of P Heath to the TMG
	8.0	Dec 12th 2021	Alasdair Munro	• Addition of Appendix E and section 9.1.12 for the Young Adult Fractional Dosing Sub-Study with accompanying table section 9.1.7.3<br>• Addition of Appendix F section 9.1.13 for the Fourth Dose Booster Sub-Study with accompanying table section 9.1.7.4<br>• Addition of secondary outcomes regarding immunogenicity, safety and reactogenicity for the Young Adult Fractional Dosing Sub-Study and Fourth Dose Sub-Study<br>• Addition of section 13.5.1 Causality assessment for participants who have received additional external vaccines, clarifying that causality should be determined as “not related” from the point of external vaccine administration onwards<br>• Note of Schedule of Attendances and Blood Sampling tables that participants from Stage 1 who received VLA2001 or CVnCoV and had an additional external COVID-19 vaccine can attend for phone visit only on D242 and should not have blood samples collected<br>• Note on section 11.6.5 COVID-19 episodes, Schedule of Attendances and Blood Sampling tables that after unblinding of all Stage 1 participants, in person visits for C19 episodes and collection of samples is no longer required; C19 episodes will just be

				recorded as AE/AESI and an initial contact form with C19 eCRF completed.<br>• Amended typographical error in section 11.5.1 Unblinding of all Stage 1 trial participants<br>• Removed legacy reference to a day 308 visit from section 9.1.4<br>• D365 visits have been brought forwards to D242. Schedules and endpoints have been updated. Participants that take part in the fourth dose sub-study will have their final visit in the study at 3 months post dose.
	8.1	January 11th 2022	Alasdair Munro<br>Stephen Saich	• Appendix D: Blood Sampling for the main phase of the trial has been corrected to clarify that all participants that received CureVac and Valneva vaccines will not have blood samples collected at D242, in line with the rest of the protocol.<br>• Text has been added on re-consent via telephone at D242 visits within the Informed Consent section.<br>• Young Adult Fractional Doses Sub-Study: change to include any positive acute COVID-19 test (including rt-PCR, Lateral flow device or Saliva LAMP testing) will be considered a positive COVID-19 diagnosis for the purpose of randomization.<br>• Clarified section 13.13 regarding contraception and pregnancy are not applicable to the fourth dose or young adult fractional dosing sub-study

	9.0	January 18th 2022	Alasdair Munro	• Addition of Appendix G for Omicron specific fourth dose booster sub-study<br>• Addition of secondary immunogenicity and safety outcomes for the Omicron specific fourth dose booster sub-study<br>• Amended references to Stage 3 of the CovBoost study to direct to the Omicron specific fourth dose booster sub-study in Appendix G<br>• Addition of day 14 VNA analysis at PHE to the Fourth dose booster sub-study<br>• Removal of cholecystitis as expected SAR for NVXCoV2373 following change to the IB<br>• Addition of UCD242 visit 8 months after the unblinded control group received an active COVID-19 vaccine to replace their original day 242 visit.<br>• Clarification that unblinded control participants who have not received a 3rd dose booster by the time of booking day 242 visits can be withdrawn (section 9.1.10.1)<br>• Clarification that positive test for COVID-19 within the past 28 days qualifies as a temporary delay criteria for the young adult fractional dosing sub-study (section 29.2.3)
	9.1	3rd February 2022	Matthew Stokes	• Correction of typographical errors in outcomes and inclusion criteria of the Omicron specific fourth dose booster sub-study to clarify that participants aged 30 or above will be recruited in line with other phases of the trial.

	10.0	28th February 2022	Stephen Saich	• Following the announcement of a Spring 2022 booster campaign for those aged over 75 and those that are considered clinically vulnerable, the window for the D242 visit for participants that previously received control vaccine has been amended from 7-9 months to 6-9 months post booster vaccination to enable final follow up visits to be performed prior to participants receiving a fourth dose via the NHS vaccination program.<br>• Removal of pseudo-neutralising antibodies as an outcome measure for the Omicron Specific Fourth Dose Booster Sub-Study and External Vaccine Trial Sub-Study<br>• Removal of pseudo-neutralising antibodies as an endpoint at D242 visits in the original phase of the trial and for the unblinded control cohort.<br>• Minor clarifications and corrections to typographical errors.

27 Appendix C: Toxicity grading scale for lab AEs

**Haematology**			Lab Range	Grade 1	Grade 2	Grade 3	Grade 4
Haemoglobin Absolute	**Male**	g/l	130 - 170	115-125	100-114	85-99	<85
Haemoglobin Absolute	**Female**		120 - 150	105-113	90-104	80-89	<80
Haemoglobin Change from Baseline (Decrease)			n/a	10-15	16-20	21-50	>50
White Blood Cells	**Elevated**	x109/l	11	11.5-15.00	15.01-20	20.01-25	>25
White Blood Cells	**Low**		4.0	2.5-3.5	1.5-2.49	1.0-1.49	<1.0
Platelets	**Low**		150-400	125-140	100-124	25-99	<25
Neutrophils	**Low**		2.0-7.0	1.5-1.99	1.0-1.49	0.5-0.99	<0.50
Lymphocytes	**Low**		1.0-4.0	0.75-0.99	0.5-0.74	0.25-0.49	<0.25
Eosinophils	**Elevated**	x109/l	0.02 - 0.5	0.65-1.5	1.51-5.00	>5.00	Hypereosinophilia
**Biochemistry**							
Sodium	**Elevated**	mmol/l	145	146-147	148-149	150-155	>155
Sodium	**Low**		135	132-134	130-131	125-129	<125
Potassium	**Elevated**	mmol/l	5	5.1-5.2	5.3-5.4	5.5-6.5	>6.5
Potassium	**Low**		3.5	3.2-3.3	3.1	2.5-3.0	<2.5
Urea	**Elevated**	mmol/l	2.5 - 7.4	8.2-9.3	9.4-11.0	>11.0	Requires dialysis
Creatinine	**Elevated**	µmol/l	49 - 104	1.1-1.5xULN<br>114-156	>1.5-3.0xULN<br>157-312	>3.0xULN<br>>312	Requires dialysis
Bilirubin	**Normal LFTs**	µmol/l	0-21	1.1-1.5xULN<br>23-32	>1.5-2xULN<br>33-42	>2-3xULN<br>43-63	>3xULN<br>≥64
Bilirubin	**Abnormal LFTs**	µmol/l	0 - 21	1.1-1.25xULN<br>23-26	>1.25-1.5xULN<br>27-32	>1.5-1.75xULN<br>33-37	>1.75xULN<br>>37
ALT		IU/l	10 - 45	1.1-2.5xULN<br>49-112	>2.-5xULN<br>113-225	>5-10xULN<br>226-450	>10xUPN<br>>450
Alk Phosphatase	**Elevated**	IU/l	30 -130	1.1-2xULN<br>143-260	>2.-3xULN<br>261-390	>3-10xULN<br>391-1300	>10xULN<br>>1300
Albumin		g/l	32-50	28-31	25-27	<25	-

Normal lab ranges may vary between sites and should be adapted accordingly

28 Appendix D: Blood Sampling

General cohort (sites collecting Lithium Heparin samples)

	**V1**	**V2**	**V3**	**V4**	**(VPP)<br>Only if enter C19P ‡**
**Study timeline**	**D0**	**D28**	**D84**	**D242†**	**(D0-D242)**
**Safety bloods**	1 x FBC (up to 2ml)<br>1 x Biochem (up to 5ml)	1 x FBC (up to 2ml)<br>1 x Biochem (up to 5ml)			1 x FBC (up to 2ml)<br>1 x biochem (up to 5ml)
**COVID-19 vaccination**	**X**				
**Primary endpoint**		**Nexelis**<br>Anti-spike IgG			
**Secondary endpoints**	**PHE**<br>Neutralising Ab<br>Anti-N IgG<br><br>**Nexelis**<br>Pseudo-neut Ab<br>Anti-spike IgG<br><br>**IMMUNOTEC**<br>ELIspot	**PHE**<br>Neutralising Ab<br><br>**Nexelis**<br>Pseudo-neut Ab<br><br>**IMMUNOTEC**<br>ELIspot	**PHE**<br>Neutralising Ab<br>Anti-N IgG<br><br>**Nexelis**<br>Pseudo-neut Ab<br>Anti-spike IgG<br><br>**IMMUNOTEC**<br>ELIspot	**PHE**<br>Neutralising Ab<br>Anti-N IgG<br><br>**Nexelis**<br>Anti-spike IgG<br><br>**IMMUNOTEC**<br>ELIspot	**PHE**<br>Neutralising Ab<br>Anti-N IgG<br><br>**Nexelis**<br>Pseudo-neut Ab<br>Anti-spike IgG<br><br>**IMMUNOTEC**<br>ELIspot
**No of tubes & colours**	Up to 2x10ml Red<br><br>Up to 2x10ml Green (LiHep)*<br><br>1 x FBC (up to 2ml)<br>1 x Biochem (up to 5ml)	Up to 2x10ml Red<br><br>Up to 2x10ml Green (LiHep)*<br><br>1 x FBC (up to 2ml)<br>1 x Biochem (up to 5ml)	Up to 2x10ml Red<br><br>Up to 2x10ml Green (LiHep)*	Up to 2x10ml Red<br><br>Up to 2x10ml Green (LiHep)*	Up to 2x10ml Red<br><br>Up to 2x10ml Green (LiHep)<br><br>1 x FBC (up to 2ml)<br>1 x Biochem (up to 5ml)
**Total volume per visit**	Up to 47ml	Up to 47ml	Up to 40ml	Up to 40ml	Up to 47ml
**Total volume by end of study**	At any point in the study, a repeat of safety bloods may be recommended at the clinical discretion of the investigator. This will result in up to an extra 7ml per repeat blood sample.			Up to 174ml	+ Up to 47ml per C-19 pathway attended

***Lithium Heparin tube or equivalent** † Participants who received VLA2001 or CVnCoV should not have blood samples collected at D242 ‡ C19 visits and samples are not required after unblinding of all Stage 1 participants

General Cohort at sites <u>NOT</u> collecting Lithium Heparin samples

	V1	V2	V3	V5	(VPP)<br>Only if enter C19P‡
**Study timeline**	D0	D28	D84	D242†	(D0-D242)
**Safety bloods**	1 x FBC (up to 2ml)<br>1 x Biochem (up to 5ml)	1 x FBC (up to 2ml)<br>1 x Biochem (up to 5ml)			1 x FBC (up to 2ml)<br>1 x Biochem (up to 5ml)
**COVID-19 vaccination**	**X**				
**Primary endpoint**		**Nexelis**<br>Anti-spike IgG			
**Secondary endpoints**	**PHE**<br>Neutralising Ab<br>Anti-N IgG<br><br>**Nexelis**<br>Pseudo-neut Ab<br>Anti-spike IgG	**PHE**<br>Neutralising Ab<br><br>**Nexelis**<br>Pseudo-neut Ab	**PHE**<br>Neutralising Ab<br>Anti-N IgG<br><br>**Nexelis**<br>Pseudo-neut Ab<br>Anti-spike IgG	**PHE**<br>Neutralising Ab<br>Anti-N IgG<br><br>**Nexelis**<br>Anti-spike IgG	**PHE**<br>Neutralising Ab<br>Anti-N IgG<br><br>**Nexelis**<br>Pseudo-neut Ab<br>Anti-spike IgG
**No of tubes & colours**	Up to 20ml Red<br><br>1 x FBC (up to 2ml)<br>1 x Biochem (up to 5ml)	Up to 20ml Red<br><br>1 x FBC (up to 2ml)<br>1 x Biochem (up to 5ml)	Up to 20ml Red	Up to 20ml Red	Up to 20ml Red<br><br>1 x FBC (up to 2ml)<br>1 x Biochem (up to 5ml)
**Total volume per visit**	Up to 27ml	Up to 27ml	Up to 20ml	Up to 20ml	Up to 27ml
**Total volume by end of study**	At any point in the study, a repeat of safety bloods may be recommended at the clinical discretion of the investigator. This will result in up to an extra 7ml per repeat blood sample.			Up to 94ml	+ Up to 27ml per C-19 pathway attended

† Participants who received VLA2001 or CVnCoV should not have blood samples collected at D242

‡ C19 visits and samples are not required after unblinding of all Stage 1 participants

Immunology Cohort

	V1	V1A	V1B	V2	V3	V5	(VPP)<br>Only if C19P‡
**Study timeline**	**D0**	**D7**	**D14**	**D28**	**D84**	**D242†**	**(D0-D242)**
**Safety bloods**	X	X		X			X
**COVID-19 vaccination**	**X**						
**Primary endpoint**				**Nexelis**<br>Anti-spike IgG			
**Secondary endpoints**	**PHE**<br>Neutralising Ab<br>Anti-N IgG<br>**Nexelis**<br>Pseudo-neut Ab<br>Anti-spike IgG<br>**OVG**<br>Functional Abs<br>**IMMUNOTEC**<br>ELISpot<br>ICS	**Nexelis**<br>Anti-spike IgG	**IMMUNOTEC**<br>ELISpot<br>ICS	**PHE**<br>Neutralising Ab<br>**Nexelis**<br>Pseudo-neut Ab<br>**OVG**<br>Functional Abs<br>**IMMUNOTEC**<br>ELISpot	**PHE**<br>Neutralising Ab<br>Anti-N IgG<br>**Nexelis**<br>Pseudo-neut Ab<br>Anti-spike IgG<br>**IMMUNOTEC**<br>ELISpot	**PHE**<br>Neutralising Ab<br>Anti-N IgG<br>**Nexelis**<br>Anti-spike IgG<br>**OVG**<br>Functional Abs<br>**IMMUNOTEC**<br>ELISpot	**PHE**<br>Neutralising Ab<br>Anti-N IgG<br>**Nexelis**<br>Pseudo-neut Ab<br>Anti-spike IgG<br>**IMMUNOTEC**<br>ELISpot<br>ICS
**No of tubes & colours**	Up to 20ml Red<br>Up to 40ml Green (LiHep)*<br>1xFBC (up to 2ml)<br>1xBiochem (up to 5ml)	Up to 20ml Red<br>1xFBC (up to 2ml)<br>1xBiochem (up to 5ml)	Up to 20ml Red<br>Up to 40ml Green (LiHep)*	Up to 20ml Red<br>Up to 30ml Green (LiHep)*<br>1xFBC (up to 2ml)<br>1xBiochem (up to 5ml)	Up to 20ml Red<br>Up to 30ml Green (LiHep)*	Up to 20ml Red<br>Up to 30ml Green (LiHep)*	Up to 20ml Red<br>Up to 40ml Green (LiHep)*<br>1xFBC (up to 2ml)<br>1xBiochem (up to 5ml)
**Total vol. per visit**	Up to 67ml	Up to 27ml	Up to 60ml	Up to 57ml	Up to 50ml	Up to 50ml	Up to 67ml
**Total volume (study end)**	At any point in the study, a repeat of safety bloods may be recommended at the clinical discretion of the investigator. This will result in up to an extra 7ml per repeat blood sample.					Up to 311ml	+ Up to 67ml per C-19P attended

*LiHep tube or equivalent † Participants who received VLA2001 or CVnCoV should not have blood samples collected at D242 ‡ C19 visits and samples are not required after unblinding of all Stage 1 participants

Additional Booster visit bloods for unblinded participants from the control arms

General cohort (with LiHep)

Study timeline	UCD0	UCD14	UCD28	UCD242
COVID-19 vaccination	X			
Secondary endpoints	PHE<br>Neutralising Ab<br>Anti-N IgG<br><br>Nexelis<br>Pseudo-neut Ab<br>Anti-spike IgG<br><br>IMMUNOTEC<br>ELIspot<br><br>Troponin	Nexelis<br><br>Anti-spike IgG<br><br>Troponin	PHE<br>Neutralising Ab<br><br>Nexelis<br>Pseudo-neut Ab<br>Anti-spike IgG<br><br>IMMUNOTEC<br>ELIspot	PHE<br>Neutralising Ab<br>Anti-N IgG<br><br>Nexelis<br>Anti-spike IgG<br><br>IMMUNOTEC<br>ELIspot
No of tubes & colours	Up to 2x10ml Red<br><br>Up to 2x10ml Green (LiHep)*<br><br>1xFBC (up to 2ml)<br>1xBiochem (up to 5ml)	10ml Red	Up to 2x10ml Red<br><br>Up to 2x10ml Green (LiHep)*<br><br>1xFBC (up to 2ml)<br>1xBiochem (up to 5ml)	Up to 2x10ml Red<br><br>Up to 2x10ml Green (LiHep)*
Total volume per visit	Up to 47ml	Up to 10ml	Up to 47ml	Up to 40ml

*LiHep tube or equivalent

UC = Unblinded Control

General cohort (without LiHep)

Study timeline	UCD0	UCD14	UCD28	UCD242
COVID-19 vaccination	X			
Secondary endpoints	PHE<br>Neutralising Ab<br>Anti-N IgG<br><br>Nexelis<br>Pseudo-neut Ab<br>Anti-spike IgG<br><br>Troponin	Nexelis<br><br>Anti-spike IgG<br><br>Troponin	PHE<br>Neutralising Ab<br><br>Nexelis<br>Pseudo-neut Ab<br>Anti-spike IgG	PHE<br>Neutralising Ab<br>Anti-N IgG<br><br>Nexelis<br>Anti-spike IgG
No of tubes & colours	Up to 20ml Red<br><br>1xFBC (up to 2ml)<br>1xBiochem (up to 5ml)	10ml Red	Up to 20ml Red<br><br>1xFBC (up to 2ml)<br>1xBiochem (up to 5ml)	Up to 20ml Red
Total volume per visit	Up to 27ml	Up to 10ml	Up to 27ml	Up to 20ml

Additional Booster visit bloods for unblinded participants from the control arms

Immunology cohort

Study timeline	UCD0	UCD14	UCD28	UCD242
**COVID-19 vaccination**	**X**			
**Exploratory endpoints**	**PHE**<br>Neutralising Ab<br>Anti-N IgG<br><br>**Nexelis**<br>Pseudo-neut Ab<br>Anti-spike IgG<br><br>**OVG**<br>Functional Abs<br><br>**IMMUNOTEC**<br>ELISpot<br>ICS<br><br>Troponin	**Nexelis**<br><br>Anti-spike IgG<br><br>**IMMUNOTEC**<br>ELISpot<br>ICS<br><br>Troponin	**PHE**<br>Neutralising Ab<br><br>**Nexelis**<br>Pseudo-neut Ab<br>Anti-spike IgG<br><br>**OVG**<br>Functional Abs<br><br>**IMMUNOTEC**<br>ELISpot	**PHE**<br>Neutralising Ab<br>Anti-N IgG<br><br>**Nexelis**<br>Anti-spike IgG<br><br>**OVG**<br>Functional Abs<br><br>**IMMUNOTEC**<br>ELISpot
**No of tubes & colours**	Up to 20ml Red<br><br>Up to 30ml Green (LiHep)*<br><br>1xFBC (up to 2ml)<br>1xBiochem (up to 5ml)	Up to 20ml Red<br><br>Up to 30ml Green (LiHep)*	Up to 20ml Red<br><br>Up to 30ml Green (LiHep)*<br><br>1xFBC (up to 2ml)<br>1xBiochem (up to 5ml)	Up to 20ml Red<br><br>Up to 30ml Green (LiHep)*
**Total vol. per visit**	Up to 57ml	Up to 50ml	Up to 57ml	Up to 50ml

*LiHep tube or equivalent

UC = Unblinded Control

External Vaccine Trial Sub-Study Participants

Immunology Cohort

	V1	V2	V3	V4	V5
**Study timeline**	**D0**	**D14**	**D28**	**D84**	**D242**
**Safety bloods**	X	X	X		
**COVID-19 vaccination**	**X**				
**Exploratory Endpoints**	**PHE**<br>Neutralising Ab<br>Anti-N IgG<br><br>**Nexelis**<br>Anti-spike IgG<br><br>**OVG**<br>Functional Abs<br><br>**IMMUNOTEC**<br>ELISpot<br>ICS<br><br>Troponin	**Nexelis**<br>Anti-spike IgG<br><br>**IMMUNOTEC**<br>ELISpot<br>ICS<br><br>Troponin	**PHE**<br>Neutralising Ab<br><br>**Nexelis**<br>Anti-spike IgG<br><br>**OVG**<br>Functional Abs<br><br>**IMMUNOTEC**<br>ELISpot	**PHE**<br>Neutralising Ab<br>Anti-N IgG<br><br>**Nexelis**<br>Anti-spike IgG<br><br>**IMMUNOTEC**<br>ELISpot	**PHE**<br>Neutralising Ab<br>Anti-N IgG<br><br>**Nexelis**<br>Anti-spike IgG<br><br>**OVG**<br>Functional Abs<br><br>**IMMUNOTEC**<br>ELISpot
**No of tubes & colours**	Up to 20ml Red<br><br>Up to 40ml Green (LiHep)*<br><br>1xFBC (up to 2ml)<br>1xBiochem (up to 5ml)	Up to 20ml Red<br><br>Up to 40ml Green (LiHep)*<br><br>1xFBC (up to 2ml)<br>1xBiochem (up to 5ml)	Up to 20ml Red<br><br>Up to 30ml Green (LiHep)*<br><br>1xFBC (up to 2ml)<br>1xBiochem (up to 5ml)	Up to 20ml Red<br><br>Up to 30ml Green (LiHep)*	Up to 20ml Red<br><br>Up to 30ml Green (LiHep)*
**Total vol. per visit**	Up to 67ml	Up to 67ml	Up to 57ml	Up to 50ml	Up to 50ml
**Total volume (study end)**	At any point in the study, a repeat of safety bloods may be recommended at the clinical discretion of the investigator. This will result in up to an extra 7ml per repeat blood sample.				Up to 291ml

* LiHep sample or equivalent, only applicable for sites collecting LiHep samples

External Vaccine Trial Sub-Study Participants

General Cohort (WITH LiHep)

	V1	V2	V3	V4	V5
**Study timeline**	**D0**	**D14**	**D28**	**D84**	**D242**
**Safety bloods**	X	X	X		
**COVID-19 vaccination**	**X**				
**Exploratory Endpoints**	**PHE**<br>Neutralising Ab<br>Anti-N IgG<br><br>**Nexelis**<br>Anti-spike IgG<br><br>**IMMUNOTEC**<br>ELISpot<br><br>Troponin	**Nexelis**<br>Anti-spike IgG<br><br>Troponin	**PHE**<br>Neutralising Ab<br><br>**Nexelis**<br>Anti-spike IgG<br><br>**IMMUNOTEC**<br>ELISpot	**PHE**<br>Neutralising Ab<br>Anti-N IgG<br><br>**Nexelis**<br>Anti-spike IgG<br><br>**IMMUNOTEC**<br>ELISpot	**PHE**<br>Neutralising Ab<br>Anti-N IgG<br><br>**Nexelis**<br>Anti-spike IgG<br><br>**IMMUNOTEC**<br>ELISpot
**No of tubes & colours**	Up to 20ml Red<br><br>Up to 20ml Green (LiHep)*<br><br>1xFBC (up to 2ml)<br>1xBiochem (up to 5ml)	Up to 20ml Red<br><br>1xFBC (up to 2ml)<br>1xBiochem (up to 5ml)	Up to 20ml Red<br><br>Up to 20ml Green (LiHep)*<br><br>1xFBC (up to 2ml)<br>1xBiochem (up to 5ml)	Up to 20ml Red<br><br>Up to 20ml Green (LiHep)*	Up to 20ml Red<br><br>Up to 20ml Green (LiHep)*
**Total vol. per visit**	Up to 47ml	Up to 27ml	Up to 47ml	Up to 40ml	Up to 40ml
**Total volume (study end)**	At any point in the study, a repeat of safety bloods may be recommended at the clinical discretion of the investigator. This will result in up to an extra 7ml per repeat blood sample.				Up to 221ml

External Vaccine Trial Sub-Study Participants

General Cohort (WITHOUT LiHep)

	V1	V2	V3	V4	V5
**Study timeline**	**D0**	**D14**	**D28**	**D84**	**D242**
**Safety bloods**	X	X	X		
**COVID-19 vaccination**	**X**				
**Exploratory Endpoints**	**PHE**<br>Neutralising Ab<br>Anti-N IgG<br><br>**Nexelis**<br>Anti-spike IgG<br><br>Troponin	**Nexelis**<br>Anti-spike IgG<br><br>Troponin	**PHE**<br>Neutralising Ab<br><br>**Nexelis**<br>Anti-spike IgG	**PHE**<br>Neutralising Ab<br>Anti-N IgG<br><br>**Nexelis**<br>Anti-spike IgG	**PHE**<br>Neutralising Ab<br>Anti-N IgG<br><br>**Nexelis**<br>Anti-spike IgG
**No of tubes & colours**	Up to 20ml Red<br><br>1xFBC (up to 2ml)<br>1xBiochem (up to 5ml)	Up to 20ml Red<br><br>1xFBC (up to 2ml)<br>1xBiochem (up to 5ml)	Up to 20ml Red<br><br>1xFBC (up to 2ml)<br>1xBiochem (up to 5ml)	Up to 20ml Red	Up to 20ml Red
**Total vol. per visit**	Up to 27ml	Up to 27ml	Up to 27ml	Up to 20ml	Up to 20ml
**Total volume (study end)**	At any point in the study, a repeat of safety bloods may be recommended at the clinical discretion of the investigator. This will result in up to an extra 7ml per repeat blood sample.				Up to 121ml

29 Appendix E: Young Adult Fractional Dosing Sub-Study

29.1 Rationale

Following analysis of the results of stage one of the COV-BOOST study, a half dose of BNT162b2 was found to have comparable immunogenicity to a full dose of BNT162b2, with a similar and acceptable side effect profile. A half dose of mRNA1273 has been licensed and deployed as a 3rd dose booster by the NHS. Initial data from the Pfizer own immunobridging studies of children aged 5 – 11y who received 10mcg of BNT162b2 showed equivalent immune responses to those seen in the studies of 12 – 15y who received 30mcg. As young people generally have a more robust immune response and experience more side effects from vaccination than elderly people, the Coalition for Epidemic Preparedness Innovations (CEPI) have requested a sub-study of fractional doses of mRNA vaccines given to young adults (aged 18 – 30). Dose sparing regimes also allow the same amount of vaccine supply to be distributed to more people.

29.2 Participants and eligibility

Participants will be young adults aged 18 – 30 who have received 2 doses of BNT162b2 or mRNA-1273 at least 3 months (84 days) prior to vaccination. Eligibility criteria are:

29.2.1 Inclusion Criteria

- Participant is willing and able to give written informed consent for participation in the trial.
- Male or Female, aged 18 to 30 years and in good health as determined by a trial clinician. Participants may have well controlled or mild-moderate comorbidity.
- In the Investigator's opinion, is able and willing to comply with all trial requirements.
- Willing to allow their General Practitioner and consultant, if appropriate, to be notified of participation in the trial
- Willing to allow investigators to discuss the volunteer's medical history with their General Practitioner and access all medical records when relevant to study procedures.
- Agreement to refrain from blood donation during the study.
- Has received 2 doses of BNT162b2 or mRNA-1273 and is at least 3 months (84 days) since their second dose by day 0

29.2.2 Exclusion Criteria

The participant may not enter the trial if ANY of the following apply:

- Receipt of any vaccine (licensed or investigational) other than the study intervention within 30 days before and after each study vaccination (one week for licensed seasonal influenza vaccine or pneumococcal vaccine)

- Prior or planned receipt of any other investigational or licensed vaccine or product likely to impact on interpretation of the trial data (e.g. adenovirus vectored vaccines, any coronavirus vaccines)
- Administration of immunoglobulins and/or any blood products within the three months preceding the planned administration of the vaccines
- Any confirmed or suspected immunosuppressive or immunodeficient state; asplenia; recurrent severe infections and use of immunosuppressant medication within the past 6 months, except topical steroids or short-term oral steroids (course lasting ≤14 days)
- History of allergic disease or reactions likely to be exacerbated by any component of study vaccines (e.g. hypersensitivity to the active substance or any of the SmPC-listed ingredients of the Pfizer vaccine)
- Any history of anaphylaxis
- Current diagnosis of or treatment for cancer (except basal cell carcinoma of the skin and cervical carcinoma in situ)
- Bleeding disorder (e.g. factor deficiency, coagulopathy or platelet disorder), or prior history of significant bleeding or bruising following IM injections or venepuncture
- Continuous use of anticoagulants, such as coumarins and related anticoagulants (i.e. warfarin) or novel oral anticoagulants (i.e. apixaban, rivaroxaban, dabigatran and edoxaban)
- Suspected or known current alcohol or drug dependency
- Any other significant disease, disorder or finding which may significantly increase the risk to the volunteer because of participation in the study, affect the ability of the volunteer to participate in the study or impair interpretation of the study data
- Severe and/or uncontrolled cardiovascular disease, respiratory disease, gastrointestinal disease, liver disease, renal disease, endocrine disorder and neurological illness (mild/moderate well controlled comorbidities are allowed)
- History of active or previous auto-immune neurological disorders (e.g. multiple sclerosis, Guillain-Barre syndrome, transverse myelitis). Bell's palsy will not be an exclusion criterion
- Significant renal or hepatic impairment
- Participant with life expectancy of less than 6 months
- Participants who have participated in another research trial involving an investigational product in the past 12 weeks.
- Insufficient level of English language to undertake all study requirements in opinion of the Investigators except where translation has been able to be provided and is available.

29.2.3 Temporary exclusion criteria

If at Visit 1 Screening & Vaccination the volunteer has any of the following, they will not be enrolled that day.

- Acute respiratory illness (moderate or severe illness with or without fever)

- Fever (oral temperature greater than 37.8°C)

- Positive test for COVID-19 within the past 28 days

They may be considered for enrolment later in the trial; if they recover in sufficient time.

29.3 Trial design

We will aim to recruit 961 participants, who will be randomised 1:1:1:1 to receive a single dose of either BNT162b2 30mcg, BNT162b2 10mcg, mRNA-1273 50mcg or mRNA-1273 25mcg. Recruitment and randomisation will be stratified according to history of confirmed SARS-CoV-2 infection, aiming to recruit 465 participants with self-reported positive acute test for COVID-19 (including rt-PCR, lateral flow tests and saliva LAMP) and 496 participants with no history of self-reported positive COVID-19 test. A subgroup of n=25 per arm within each of the two groups will be included in the immunology cohort to have additional blood samples taken for exploratory objectives.

All participants will be monitored for adverse events and have bloods for immunogenicity taken at day 0, 14, 28, 84, and 242. Safety bloods will be performed at day 0, 14 and 28, including Troponin at day 0 and 14. Participants will be asked to record symptoms in an e-diary in the same manner as the Stage 1 main study, and adverse events will be recorded in the same way.

COVID-19 infection episodes (confirmed by rt-PCR, lateral flow tests or saliva LAMP) will be recorded as AE/AESIs and initial contact forms with C19 participants will be completed, but no additional visits or sample collection will take place. Participants will be unblinded following the D84 immunogenicity bloods or at the timepoint after day 28 should a participant or participants require the information to be entered onto the NHS app/devolved nation equivalent system for internal UK certification or foreign travel.

29.3.1 Young Adults Fractional Dosing Sub-Study group allocation

Group	Subgroup	Booster	Visits
Young Adult Fractional Dosing	YA-BNT30mcg	BNT162b2 30mcg	D0, D14, D28, D84, D242
	YA-BNT10mcg	BNT162b2 10mcg	
	YA-MOD50mcg	mRNA-1273 50mcg	
	YA-MOD25mcg	mRNA-1273 25mcg	

29.4 Randomisation and blinding

Computer generated randomisation list will be prepared by the study statistician. Participants will be randomised 1:1:1:1 to the 4 vaccines using block randomisation. Random block sizes of 4 or 8 will be used. The randomisation

will be stratified by the study sites and history of positive test for COVID-19 infection (yes/no). The blinding will follow the main trial.

29.5 Statistics

The fractional dose sub-study uses a non-inferiority design to compare between schedules with a fractional dose versus a full dose BNT162b2 as the 3rd dose. The comparisons will be conducted separately in the seronegative and seropositive participants (determined by Anti-nucleocapsid IgG at pre-3rd dose). We will recruit participants based on their self-report history of confirmed COVID-19 infection (including rt-PCR, lateral flow tests and saliva LAMP) and define the populations for analysis using anti-nucleocapsid IgG once it becomes available.

The sample size calculation was conducted separately for participant with/without a self-report history of PCR confirmed COVID-19 infection. All the below assumption were based on the published COV-BOOST data on 28 days post 3rd dose.

The sample calculation is based on the following assumptions:

- Standard deviation=0.32 based on the D28 post 3rd dose of ½ BNT in the BNT/BNT primed population aged 30-70 yrs (n=51)
- Non-inferiority margin of 0.67 (WHO recommendation)
- Type-I error of two-sided 0.0167 (adjusting for 3 comparisons in each population)
- Power of 90%
- Among the participants with no PCR-confirmed COVID-19 (self-reported), the rate of seropositive is 20% (based on the ongoing COMCOV3 study, n=80)
- Among the participants with self-reported PCR-confirmed COVID-19, the rate of anti-N negative is 15% (based on COV-boost, n=17 out of 129)
- 5% of loss of follow up

Based on the above assumptions, the study will need to recruit an effective sample size of 93 per arm. After accounting for the attrition rate listed above, we will need to recruit N=93/(1-20%) *4= 465 participants with a self-report history of COVID-19 infection, and to recruit N=93/(1-25%) *4= 496 participants with no self-report history of COVID-19 infection.

The safety and reactogenicity analysis will follow the analysis in the main COV-BOOST study. For immunogenicity analysis, the primary analysis populations for the two groups will be on the per-protocol basis:

- Participants with no self-report history of COVID-19 infection who are seronegative pre-3rd dose with no infection within 14 days post 3rd dose, and whose primary endpoints are available. In addition, participants with protocol deviations on the timing of 3rd dose or primary endpoint visit will be excluded;

- Participants with a self-report history of COVID-19 infection who are seropositive pre-3rd dose with no infection within 14 days post 3rd dose, and whose primary endpoints are available. In addition, participants with protocol deviations on the timing of 3rd dose or primary endpoint visit will be excluded.

The sensitivity analyses will also be conducted on the modified intention-to-treat basis by including participants with protocol deviations on the timing of 3rd dose or primary endpoint.

The primary endpoint is anti-spike IgG measured by standardised ELISA at Day 14 post 3rd dose. The geometric mean concentrations (GMC) of anti-spike IgG will be compared between fractional dose arms and full dose BNT162b2 arm under the hypothesis:

H_0: $GMC_{\text{fractional dose}} / GMC_{\text{full dose}} \leq 0.67$ or $log10\ GMC_{\text{fractional dose}} - log10\ GMC_{\text{full dose}} \leq -0.174$;

H_1: $GMC_{\text{fractional dose}} / GMC_{\text{full dose}} > 0.67$ or $log10\ GMC_{\text{fractional dose}} - log10\ GMC_{\text{full dose}} > -0.174$.

The anti-spike IgG data will be transferred using logarithmic transformations (base 10) to render a normal distribution. We will test the above hypothesis using the multiple regression on log_{10}GMC adjusting for randomisation design variables and the pre-specified prognostic factors, if any. The adjusted mean difference of log_{10}GMC will be presented with the two-sided 98.3% confidence interval (CI) in the primary analyses. We will claim fractional dose arm is non-inferior to full dose arm if the lower CI lies above -0.174 for the adjusted mean difference of log_{10}GMC.

A fully detailed statistical analysis plan will be prepared and will be signed off by the Chief Investigator prior to conducting any data analyses.

Interim analyses

The seropositive rates will be monitored closely to check the attrition rate as a result of policy change to PCR test from January 2022 as well as the potential impact of Omicron wave. In case that our assumption is underestimated, if the effective sample size cannot be achieved we will discuss the possibility to increase the sample size with the funder, TSC and DSMB.

The interim analysis on immunogenicity will be carried out when the primary endpoint of D14 anti-spike IgG data become available to inform the policy making during the pandemic. The aggregated results will be only shared with the relevant parties before publishing the full interim analysis. There will be stopping rules for the interim analyses and the analyses will not affect the continuation of trial.

As the assumptions on sero-status were based on a relatively older population in COV-BOOST, we will review the sero-status data once it become available. If there is a significant discrepancy between observed data and our assumptions, we will consult the funder/TSC/DSMB to explore the possibility of increase the sample size.

Missing data

The level and pattern of the missing data in the baseline variables and outcomes will be reported. The potential causes of any missing data will be investigated and documented as far as possible. Any missing data will be dealt with using methods appropriate to the conjectured missing mechanism and level of missing.

29.6 Schedule of visitation (Young Adult Fractional Dosing)

	V1	V2	V3	V4	V5
**Study timeline**	D0	D14	D28	D84	D242
**Study window**	N/A	±3	-7/+3	±14	±28
**Informed consent**	X				
**Safety bloods**	X	X	X		
**Medical history**	X				
**Interim medical history**		X	X	X	X
**Physical examination (as required)**	(X)	(X)	(X)	(X)	(X)
**Urine test (Pregnancy) (if required)**	X				
**COVID-19 vaccination**	X				
**COVID-19 immunogenicity bloods**	X	X	X	X	X
**Diary card review**		X	X		
**SAE/AESI/Medically attended AE check**		X	X	X	X
**Troponin**	X	X			

29.7 Blood sampling (Young Adult Fractional Dosing) Immunology Cohort

	V1	V2	V3	V4	V5
**Study timeline**	**D0**	**D14**	**D28**	**D84**	**D242**
**Safety bloods**	X	X	X		
**COVID-19 vaccination**	**X**				
**Exploratory Endpoints**	**PHE**<br>Neutralising Ab<br>Anti-N IgG<br><br>**Nexelis**<br>Pseudo-neut Ab<br>Anti-spike IgG<br><br>**IMMUNOTEC**<br>ELISpot<br>ICS<br><br>Troponin	**Nexelis**<br>Anti-spike IgG<br><br>**IMMUNOTEC**<br>ELISpot<br>ICS<br><br>Troponin	**PHE**<br>Neutralising Ab<br><br>**Nexelis**<br>Pseudo-neut Ab<br>Anti-spike IgG<br><br>**IMMUNOTEC**<br>ELISpot	**PHE**<br>Neutralising Ab<br>Anti-N IgG<br><br>**Nexelis**<br>Pseudo-neut Ab<br>Anti-spike IgG<br><br>**IMMUNOTEC**<br>ELISpot	**PHE**<br>Neutralising Ab<br>Anti-N IgG<br><br>**Nexelis**<br>Pseudo-neut Ab<br>Anti-spike IgG<br><br>**IMMUNOTEC**<br>ELISpot
**No of tubes & colours**	Up to 20ml Red<br><br>Up to 40ml Green (LiHep)*<br><br>1xFBC (up to 2ml)<br>1xBiochem (up to 5ml)	Up to 20ml Red<br><br>Up to 40ml Green (LiHep)*<br><br>1xFBC (up to 2ml)<br>1xBiochem (up to 5ml)	Up to 20ml Red<br><br>Up to 30ml Green (LiHep)*<br><br>1xFBC (up to 2ml)<br>1xBiochem (up to 5ml)	Up to 20ml Red<br><br>Up to 20ml Green (LiHep)*	Up to 20ml Red<br><br>Up to 20ml Green (LiHep)*
**Total vol. per visit**	Up to 67ml	Up to 67ml	Up to 57ml	Up to 40ml	Up to 40ml
**Total volume (study end)**	At any point in the study, a repeat of safety bloods may be recommended at the clinical discretion of the investigator. This will result in up to an extra 7ml per repeat blood sample.				Up to 271ml

29.8 Blood sampling (Young Adult Fractional Dosing) General Cohort with Lithium Heparin

	V1	V2	V3	V4	V5
**Study timeline**	**D0**	**D14**	**D28**	**D84**	**D242**
**Safety bloods**	X	X	X		
**COVID-19 vaccination**	**X**				
**Exploratory Endpoints**	**PHE**<br>Neutralising Ab<br>Anti-N IgG<br><br>**Nexelis**<br>Pseudo-neut Ab<br>Anti-spike IgG<br><br>**IMMUNOTEC**<br>ELISpot<br><br>Troponin	**Nexelis**<br>Anti-spike IgG<br><br>Troponin	**PHE**<br>Neutralising Ab<br><br>**Nexelis**<br>Pseudo-neut Ab<br>Anti-spike IgG<br><br>**IMMUNOTEC**<br>ELISpot	**PHE**<br>Neutralising Ab<br>Anti-N IgG<br><br>**Nexelis**<br>Pseudo-neut Ab<br>Anti-spike IgG<br><br>**IMMUNOTEC**<br>ELISpot	**PHE**<br>Neutralising Ab<br>Anti-N IgG<br><br>**Nexelis**<br>Pseudo-neut Ab<br>Anti-spike IgG<br><br>**IMMUNOTEC**<br>ELISpot
**No of tubes & colours**	Up to 20ml Red<br><br>Up to 20ml Green (LiHep)*<br><br>1xFBC (up to 2ml)<br>1xBiochem (up to 5ml)	Up to 20ml Red<br><br>1xFBC (up to 2ml)<br>1xBiochem (up to 5ml)	Up to 20ml Red<br><br>Up to 20ml Green (LiHep)*<br><br>1xFBC (up to 2ml)<br>1xBiochem (up to 5ml)	Up to 20ml Red<br><br>Up to 20ml Green (LiHep)*	Up to 20ml Red<br><br>Up to 20ml Green (LiHep)*
**Total vol. per visit**	Up to 47ml	Up to 27ml	Up to 47ml	Up to 40ml	Up to 40ml
**Total volume (study end)**	At any point in the study, a repeat of safety bloods may be recommended at the clinical discretion of the investigator. This will result in up to an extra 7ml per repeat blood sample.				Up to 201ml

29.9 Blood sampling (Young Adult Fractional Dosing) General Cohort without Lithium Heparin

	V1	V2	V3	V4	V5
**Study timeline**	**D0**	**D14**	**D28**	**D84**	**D242**
**Safety bloods**	X	X	X		
**COVID-19 vaccination**	**X**				
**Exploratory Endpoints**	**PHE**<br>Neutralising Ab<br>Anti-N IgG<br><br>**Nexelis**<br>Pseudo-neut Ab<br>Anti-spike IgG<br><br>Troponin	**Nexelis**<br>Anti-spike IgG<br><br>Troponin	**PHE**<br>Neutralising Ab<br><br>**Nexelis**<br>Pseudo-neut Ab<br>Anti-spike IgG	**PHE**<br>Neutralising Ab<br>Anti-N IgG<br><br>**Nexelis**<br>Pseudo-neut Ab<br>Anti-spike IgG	**PHE**<br>Neutralising Ab<br>Anti-N IgG<br><br>**Nexelis**<br>Pseudo-neut Ab<br>Anti-spike IgG
**No of tubes & colours**	Up to 20ml Red<br><br>1xFBC (up to 2ml)<br>1xBiochem (up to 5ml)	Up to 20ml Red<br><br>1xFBC (up to 2ml)<br>1xBiochem (up to 5ml)	Up to 20ml Red<br><br>1xFBC (up to 2ml)<br>1xBiochem (up to 5ml)	Up to 20ml Red	Up to 20ml Red
**Total vol. per visit**	Up to 27ml	Up to 27ml	Up to 27ml	Up to 20ml	Up to 20ml
**Total volume (study end)**	At any point in the study, a repeat of safety bloods may be recommended at the clinical discretion of the investigator. This will result in up to an extra 7ml per repeat blood sample.				Up to 121ml

30 Appendix F: Fourth Dose Booster Sub-Study

30.1 Rationale

Following mass deployment of 3rd dose COVID-19 booster vaccination by the NHS, and the emergence of the Omicron variant which has substantial mutations to the spike protein thought to confer a significant degree of immune escape, the question has arisen over the ability of further doses of COVID-19 vaccination to overcome this immune escape. The study question will be whether the peak spike IgG titres at D28 following a 3rd dose booster from Stage 1 can be matched at D14 following a 4th dose booster.

30.2 Participants and eligibility

Participants from the main study in Group B who received a 3rd dose booster of BNT162b2 (regardless of whether primary course was BNT162b2 or ChAdOx1-nCov19) will be invited to participate. Participants will be eligible for booster vaccination at the Fourth Dose Booster Sub-Study unless they have had a previous severe adverse reaction to mRNA vaccines or have acquired an additional COVID-19 vaccine outside of the study since enrolling. Other medical criteria will be checked prior to immunisation (including diagnosis of cancer, autoimmune conditions, neurological conditions, blood clotting conditions and pregnancy), but will not be considered a contraindication to vaccination unless the investigator feels there is a specific clinical reason to withhold vaccination for the safety of the participant.

30.3 Trial Design

We will aim to recruit as many of the approximately 222 eligible participants from stage one as possible. Participants will be randomised 1:1 to receive either BNT162b2 or mRNA-1273 50mcg. 25 participants in each of the two arms will be included in the immunology cohort. This may include participants that were not in the immunology cohort for previous visits in the main phase of the trial.

All participants will be monitored for adverse events and have bloods for immunogenicity taken at day 0, 14. Following D14 participants will attend their final visit for the trial 3 months (+/- 14 days) from the time of their fourth dose (Day 0 referenced above).

Safety bloods will be performed at day 0 and 14 including troponin. Participants will be asked to record symptoms in an e-diary in the same manner as the Stage 1 main study, and adverse events will be recorded in the same way.

COVID-19 infection episodes (confirmed by rt-PCR, lateral flow tests or saliva LAMP) will be recorded as AE/AESIs and initial contact forms with C19 participants will be completed, but no additional visits or sample collection will take place. Participants will be unblinded following the completion of day 84 follow up visits.

30.3.1 Fourth Dose Booster Sub-Study group allocation

Group	Subgroup	Booster	Visits
Fourth Dose Booster	4D-BNT	BNT162b2	D0, D14, D84
	4D-MOD	mRNA-1273 50mcg	

30.4 Randomisation and blinding

Computer generated randomisation list will be prepared by the study statistician. Participants will be randomised 1:1 to BNT162b2 and mRNA-1273 50mcg using block randomisation. Random block sizes of 2 or 4 will be used. The randomisation will be stratified by the study sites, prime vaccine schedules (ChAd/ChAd or BNT/BNT), and age group (<70 years and ≥ 70 years). The blinding will follow the main trial.

30.5 Statistics

Since this sub-study is nested within the main COV-BOOST trial, there was no formal sample size calculation for the sub-study. The primary analysis is to compare the immune response at D14 post 4th dose and that at D28 post 3rd dose by paired comparisons under the below hypothesis:

H_0: $GMC_{\text{post 4th dose}} / GMC_{\text{post 3rd dose}} = 1$ or $log10\ GMC_{\text{post 4th dose}} - log10\ GMC_{\text{post 3rd dose}} = 0$;

H_1: $GMC_{\text{post 4th dose}} / GMC_{\text{post 3rd dose}} > 1$ or $log10\ GMC_{\text{post 4th dose}} - log10\ GMC_{\text{post 3rd dose}} > 0$.

The analysis will be done in all the participants and also be conducted separately for the below four schedules:

- ChAd/ChAd + BNT + BNT
- ChAd/ChAd + BNT + mRNA-1273
- BNT/BNT + BNT + BNT
- BNT/BNT + BNT + mRNA-1273

We will calculate the fold change between immune responses post 4th dose and post 3rd dose with 95% confidence intervals. The fold change will be transferred using logarithmic transformations (base 10) to render a normal distribution. The primary analysis population will be those who were seronegative pre-4th dose and sensitivity analysis will be conducted among all the participants.

Interim analyses

The interim analysis on immunogenicity will be carried out when the primary endpoint of D14 anti-spike IgG data become available to inform the policy making during the pandemic. The aggregated results will be only shared with the relevant parties before publishing the full interim analysis. There will be stopping rules for the interim analyses and the analyses will not affect the continuation of trial.

Missing data

The level and pattern of the missing data in the baseline variables and outcomes will be reported. The potential causes of any missing data will be investigated and documented as far as possible. Any missing data will be dealt with using methods appropriate to the conjectured missing mechanism and level of missing.

30.6 Schedule of visitation (Fourth Dose Booster)

	V1	V2	V3*
**Study timeline**	D0	D14	D84
**Study window**	N/A	±3	±14
**Informed consent**	X		
**Safety bloods**	X	X	
**Medical history**	X		
**Interim medical history**		X	X
**Physical examination (as required)**	(X)	(X)	(X)
**Urine test (Pregnancy) (if required)**	X		
**COVID-19 vaccination**	X		
**COVID-19 immunogenicity bloods**	X	X	X
**Diary card review**		X	
**SAE/AESI/Medically attended AE check**		X	X
**Troponin**	X	X	

*For participants in the fourth dose booster sub-study this visit will replace the previously scheduled D242 visit from the main phase of the trial.

30.7 Blood sampling (Fourth Dose Booster) Immunology Cohort

	V1	V2	V3*
**Study timeline**	**D0**	**D14**	**D84**
**Safety bloods**	X	X	
**COVID-19 vaccination**	**X**		
**Exploratory Endpoints**	**PHE**<br>Neutralising Ab<br>Anti-N IgG<br><br>**Nexelis**<br>Anti-spike IgG<br><br>**IMMUNOTEC**<br>ELISpot<br>ICS<br><br>Troponin	**PHE**<br>Neutralising Ab<br><br>**Nexelis**<br>Anti-spike IgG<br><br>**IMMUNOTEC**<br>ELISpot<br>ICS<br><br>Troponin	**PHE**<br>Neutralising Ab<br>Anti-N IgG<br><br>**Nexelis**<br>Anti-spike IgG<br><br>**IMMUNOTEC**<br>ELISpot
**No of tubes & colours**	Up to 20ml Red<br><br>Up to 40ml Green (LiHep)<br><br>1xFBC (up to 2ml)<br>1xBiochem (up to 5ml)	Up to 20ml Red<br><br>Up to 40ml Green (LiHep)<br><br>1xFBC (up to 2ml)<br>1xBiochem (up to 5ml)	Up to 20ml Red<br><br>Up to 30ml Green (LiHep)
**Total vol. per visit**	Up to 67ml	Up to 67ml	Up to 50ml
**Total volume (study end)**	At any point in the study, a repeat of safety bloods may be recommended at the clinical discretion of the investigator. This will result in up to an extra 7ml per repeat blood sample.		Up to 184 ml

*This visit will be the previously scheduled D242 visit for the main phase of the trial, performed 3 months post fourth dose vaccination.

30.8 Blood sampling (Fourth Dose Booster) General Cohort with Lithium Heparin

	V1	V2	V3*
**Study timeline**	**D0**	**D14**	**D84**
**Safety bloods**	X	X	
**COVID-19 vaccination**	**X**		
**Exploratory Endpoints**	**PHE**<br>Neutralising Ab<br>Anti-N IgG<br><br>**Nexelis**<br>Anti-spike IgG<br><br>**IMMUNOTEC**<br>ELISpot<br><br>Troponin	**PHE**<br>Neutralising Ab<br><br>**Nexelis**<br>Anti-spike IgG<br><br>Troponin	**PHE**<br>Neutralising Ab<br>Anti-N IgG<br><br>**Nexelis**<br>Anti-spike IgG<br><br>**IMMUNOTEC**<br>ELISpot
**No of tubes & colours**	Up to 20ml Red<br><br>Up to 20ml Green (LiHep)*<br><br>1xFBC (up to 2ml)<br>1xBiochem (up to 5ml)	Up to 20ml Red<br><br>1xFBC (up to 2ml)<br>1xBiochem (up to 5ml)	Up to 20ml Red<br><br>Up to 20ml Green (LiHep)
**Total vol. per visit**	Up to 47ml	Up to 27ml	Up to 40ml
**Total volume (study end)**	At any point in the study, a repeat of safety bloods may be recommended at the clinical discretion of the investigator. This will result in up to an extra 7ml per repeat blood sample.		Up to 114ml

*This visit will be replace the previously scheduled D242 visit for the main phase of the trial, performed 3 months post fourth dose vaccination.

30.9 Blood sampling (Fourth Dose Booster) General Cohort without Lithium Heparin

	V1	V2	V3*
**Study timeline**	**D0**	**D14**	**D84**
**Safety bloods**	X	X	
**COVID-19 vaccination**	**X**		
**Exploratory Endpoints**	**PHE**<br>Neutralising Ab<br>Anti-N IgG<br><br>**Nexelis**<br>Anti-spike IgG<br><br>Troponin	**PHE**<br>Neutralising Ab<br><br>**Nexelis**<br>Anti-spike IgG<br><br>Troponin	**PHE**<br>Neutralising Ab<br>Anti-N IgG<br><br>**Nexelis**<br>Anti-spike IgG
**No of tubes & colours**	Up to 20ml Red<br><br>1xFBC (up to 2ml)<br>1xBiochem (up to 5ml)	Up to 20ml Red<br><br>1xFBC (up to 2ml)<br>1xBiochem (up to 5ml)	Up to 20ml Red
**Total vol. per visit**	Up to 27ml	Up to 27ml	Up to 20ml
**Total volume (study end)**	At any point in the study, a repeat of safety bloods may be recommended at the clinical discretion of the investigator. This will result in up to an extra 7ml per repeat blood sample.		Up to 74ml

*This visit will replace the previously scheduled D242 visit for the main phase of the trial, performed 3 months post fourth dose vaccination.

31 Appendix G: Omicron Specific Fourth Dose Booster

31.1 Rationale

The Omicron Variant of Concern (VOC) exhibits an unusually large number of mutations, including to the spike protein against which all licensed vaccines are designed. There is evidence that this has conferred a significant degree of immune escape, predominantly against neutralisation from infection (some degree of protection against severe disease and death is expected to be preserved). As a result, there have been new vaccines designed which are specifically targeted to the spike protein of the Omicron VOC, including one based on the original mRNA-1273 vaccine.

This sub-study aims to compare safety and immunogenicity responses of an omicron specific vaccine, mRNA-1273.529 as a fourth dose booster to a full dose of BNT162b2 as a fourth dose booster. The study hypothesis is that the mRNA-1273.529 as a fourth dose compared with BNT162b2 can induce higher antibodies against omicron variant and non-inferior antibodies against delta variant (which is currently still circulating worldwide).

31.2 Outcomes

The immunogenicity outcome to power the sub-study will be non-inferiority of day 28 live viral neutralisation assays against the delta variant in the mRNA-1273.529 arm compared with BNT162b2 arm, and the co-immunogenicity outcome will be superiority of day 28 live viral neutralisation assays against the omicron variant in the mRNA-1273.529 arm compared with BNT162b2. Safety will be assessed using the same outcomes as the main study (solicited AEs up to 7 days, unsolicited AEs up to 28 days, medically attended AEs up to 84 days, AESIs and SAEs for the duration of the study).

31.3 Participants and eligibility

We will recruit healthy adult volunteers aged 30 years or over with no history of previous COVID-19 infection from 2 populations: firstly, from participants in the unblinded control arm who received BNT162b2 or half dose mRNA-1273 as their third dose booster, and secondly, from the general population who received either BNT162b2 or half dose mRNA-1273 as a third dose booster at least 84 days before day 0. Eligibility criteria are:

31.3.1 Inclusion Criteria

- Participant is willing and able to give written informed consent for participation in the trial.
- Male or Female, aged 30 years or over and in good health as determined by a trial clinician. Participants may have well controlled or mild-moderate comorbidity.
- Female participants of childbearing potential must be willing to ensure that they or their partner use effective contraception from 1 month prior to first immunisation continuously until 3 months after boost

immunisation. See Section "Contraception and Pregnancy" for definition of child-bearing potential and definition of effective contraception.

- In the Investigator's opinion, is able and willing to comply with all trial requirements.
- Willing to allow their General Practitioner and consultant, if appropriate, to be notified of participation in the trial
- Willing to allow investigators to discuss the volunteer's medical history with their General Practitioner and access all medical records when relevant to study procedures.
- Agreement to refrain from blood donation during the study.
- Has received 3 doses of approved COVID-19 vaccine, with the third dose being either BNT162b2 30mcg or mRNA-1273 50mcg and is at least 3 months (84 days) since their third dose by day 0.

31.3.2 Exclusion Criteria

The participant may not enter the trial if ANY of the following apply:

- Previous receipt of a fourth COVID-19 vaccine
- Previous positive test for COVID-19, including rt-PCR, lateral flow or saliva/LAMP test.
- Receipt of any vaccine (licensed or investigational) other than the study intervention within 30 days before and after each study vaccination (one week for licensed seasonal influenza vaccine or pneumococcal vaccine)
- Prior or planned receipt of any other investigational or licensed vaccine or product likely to impact on interpretation of the trial data (e.g. COVID-19 variant specific investigational vaccines)
- Participants who are pregnant at enrolment or planning to become pregnant during the first 3 months following vaccination.
- Administration of immunoglobulins and/or any blood products within the three months preceding the planned administration of the vaccines
- Any confirmed or suspected immunosuppressive or immunodeficient state; asplenia; recurrent severe infections and use of immunosuppressant medication within the past 6 months, except topical steroids or short-term oral steroids (course lasting ≤14 days)
- History of allergic disease or reactions likely to be exacerbated by any component of study vaccines (e.g. hypersensitivity to the active substance or any of the SmPC-listed ingredients of the Pfizer vaccine)
- Any history of anaphylaxis
- Current diagnosis of or treatment for cancer (except basal cell carcinoma of the skin and cervical carcinoma in situ)
- Bleeding disorder (e.g. factor deficiency, coagulopathy or platelet disorder), or prior history of significant bleeding or bruising following IM injections or venepuncture

- Continuous use of anticoagulants, such as coumarins and related anticoagulants (i.e. warfarin) or novel oral anticoagulants (i.e. apixaban, rivaroxaban, dabigatran and edoxaban)
- Suspected or known current alcohol or drug dependency
- Any other significant disease, disorder or finding which may significantly increase the risk to the volunteer because of participation in the study, affect the ability of the volunteer to participate in the study or impair interpretation of the study data
- Severe and/or uncontrolled cardiovascular disease, respiratory disease, gastrointestinal disease, liver disease, renal disease, endocrine disorder and neurological illness (mild/moderate well controlled comorbidities are allowed)
- History of active or previous auto-immune neurological disorders (e.g. multiple sclerosis, Guillain-Barre syndrome, transverse myelitis). Bell's palsy will not be an exclusion criterion
- Significant renal or hepatic impairment
- Participant with life expectancy of less than 6 months
- Participants who have participated in another research trial involving an investigational product in the past 12 weeks.
- Insufficient level of English language to undertake all study requirements in opinion of the Investigators except where translation has been able to be provided and is available.

31.3.3 Temporary exclusion criteria

If at Visit 1 Screening & Vaccination the volunteer has any of the following, they will not be enrolled that day.

- Acute respiratory illness (moderate or severe illness with or without fever)

- Fever (oral temperature greater than 37.8°C)

They may be considered for enrolment later in the trial, if they recover in sufficient time.

31.4 Trial Design

We will aim to recruit 410 eligible participants from 9 or 10 of the study sites, including as many of the eligible participants from the unblinded control arms in stage one as possible from participating sites. Participants will be randomised 1:1 to receive either BNT162b2 or mRNA-1273.529. 50 participants in each of the two arms will be included in the immunology cohort.

All participants will be monitored for adverse events and have bloods for immunogenicity taken at day 0, 14, 28, 84, and 242. Safety bloods will be performed at day 0, 14 and 28, including troponin at day 0 and 14. Participants will be asked to record symptoms in an e-diary in the same manner as the Stage 1 main study, and adverse events will be recorded in the same way. Participants who were previously in the unblinded control arm who are enrolled in this sub-study will not attend their pre-planned day 242 visit and be moved on to the new schedule of events for

the sub-study. For the purpose of future sample analysis, their day 0 visit can be considered their day 242 visit from Stage 1.

COVID-19 infection episodes (confirmed by rt-PCR, lateral flow tests or saliva LAMP) will be recorded as AE/AESIs and initial contact forms with C19 participants will be completed, but no additional visits or sample collection will take place. Participants will be unblinded after the D242 blood test, or earlier in the event they become eligible for a 4th dose of COVID-19 vaccine in the NHS programme. Also, in the event a 4th dose is required by that participant for international travel due to external country rules regarding timing of the last vaccine, investigators may request the Sponsor for permission to unblind.

31.4.1 Omicron Variant Booster Sub-Study group allocation

Group	Subgroup	Booster	Visits
Omicron Variant Booster	OV-BNT	BNT162b2	D0, D14, D28, D84, D242
	OV-m529	mRNA-1273.529	

31.5 Randomisation and blinding

Computer generated randomisation list will be prepared by the study statistician. Participants will be randomised 1:1 to BNT162b2 and mRNA-1273.529 using block randomisation. Random block sizes of 2 or 4 will be used. The randomisation will be stratified by the study sites, cohort (general/immunology) prime vaccine schedules (adenovirus vectored (e.g. ChAd) or mRNA (BNT162b2 or mRNA-1273), and age group (<70 years and ≥ 70 years). The blinding will follow the main trial.

31.6 Statistics

The omicron specific fourth dose booster uses a non-inferiority design to power the study, comparing mRNA-1273.529 to BNT162b2 as the fourth dose (second boost). The comparisons will be conducted in the COVID-19 native participants (determined by Anti-nucleocapsid IgG at pre-4th dose). Given the infection with specific strains can significantly affect the immunogenicity against omicron and delta, we will not recruitment participants with a history of COVID-19 infection.

The sample calculation is based on the following assumptions:

- Standard deviation=0.4 at log10 scale based on the D28 neutralising antibody data against **Delta** post 3rd dose of BNT in the BNT/BNT and ChAd/ChAd primed population aged 30-70 yrs (n=50);

- Standard deviation=0.55 at log10 scale based on the D28 neutralising antibody data against **Omicron** post 3rd dose of BNT in the BNT/BNT and ChAd/ChAd primed population aged 30-70 yrs (n=50);
- Non-inferiority margin of 0.67

- Type-I error of two-sided 0.025 (adjusting for two co-outcomes)
- Power of 90%
- Among the participants with no COVID-19 infection, the rate of seropositive is 30% (based on the discussion with experts from PHE)
- 5% of loss of follow up

Based on the above assumptions, the study will need to recruit an effective sample size of 133 per arm to show non-inferiority. After accounting for the attrition rate listed above, we will need to recruit N=93/(1-35%) *2= 410 participants. With this sample size, the minimum difference to detect for superiority of antibodies against omicron is 0.239 at log10 scale or GMR of 1.73 (or 0.58). The immunogenicity cohort will used for exploratory analyses to generate hypothesis, and thus no formal sample size calculation was carried out for this cohort. The sample size of 50 per arm was therefore chosen based on practical constraints. This means we will have around 33 seronegative participants in each arm for analysis.

The safety and reactogenicity analysis will follow the analysis in the main COV-BOOST study. For immunogenicity analysis, the primary analysis populations will be on the per-protocol basis (for non-inferiority analysis), i.e. participants with no infection within 14 days post 4th dose, who are seronegative pre-4th dose, and whose primary endpoints are available. In addition, participants with protocol deviations on the timing of 4th dose or primary endpoint visit will be excluded;

For the superiority analysis on antibodies against omicron, the analysis will be conducted on the modified intention-to-treat basis. All the randomised participants who receive the study vaccine and have the endpoint data available will be the superiority analysis population. The non-inferiority analysis will be done according the vaccine received, and the superiority analysis will be done according to the randomised arm.
For non-inferiority, the sensitivity analysis will also be conducted on the modified intention-to-treat basis by including participants with protocol deviations on the timing of 4th dose or primary endpoint and according the vaccine received.

The primary endpoint is live neutralising antibodies against delta Day 28 post 4th dose. The geometric mean concentrations (GMC) of antibodies will be compared between the two arms under the hypothesis:

H_0: $GMC_{mRNA\text{-}1273.529} / GMC_{BNT162b2} \leq 0.67$ or $log10\ GMC_{mRNA\text{-}1273.529} - log10\ GMC_{BNT162b2} \leq -0.174$;

H_1: $GMC_{mRNA\text{-}1273.529} / GMC_{BNT162b2} > 0.67$ or $log10\ GMC_{mRNA\text{-}1273.529} - log10\ GMC_{BNT162b2} > -0.174$.

The neutralising antibody data will be transferred using logarithmic transformations (base 10) to render a normal distribution. We will test the above hypothesis using the multiple regression on log_{10}GMC adjusting for randomisation design variables and the pre-specified prognostic factors, if any. The adjusted mean difference of log_{10}GMC will be presented with the two-sided 97.5% confidence interval (CI) in the primary analyses. We will claim mRNA-1273.529 arm is non-inferior to BNT162b2 arm if the lower CI lies above -0.174 for the adjusted mean difference of log_{10}GMC.

A fully detailed statistical analysis plan will be prepared and will be signed off by the Chief Investigator prior to conducting any data analyses.

Interim analyses

The interim analysis on immunogenicity will be carried out when the primary endpoint of D28 neutralising antibody data becomes available to inform the policy making during the pandemic. The aggregated results will be only shared with the relevant parties before publishing the full interim analysis. There will be stopping rules for the interim analyses and the analyses will not affect the continuation of trial.

The seropositive rates will be monitored closely. In case that our assumption is underestimated and the effective sample size cannot be achieved, we will discuss the possibility to increase the sample size with the funder, TSC and DSMB.

Missing data

The level and pattern of the missing data in the baseline variables and outcomes will be reported. The potential causes of any missing data will be investigated and documented as far as possible. Any missing data will be dealt with using methods appropriate to the conjectured missing mechanism and level of missing.

31.7 Trial Interventions

31.7.1 Investigational Medicinal Product(s) (IMP) Description

31.7.1.1 Pfizer BioNTech (BNT162b2)

As described in the main protocol (section 12.1.2)

31.7.1.2 Moderna Omicron Variant vaccine (mRNA-1273.529)

The COVID-19 Moderna vaccine targeted at the omicron variant, mrna-1273.529 is based on the original RNA1273 vaccine but contained a single mRNA that encodes for the B.1.1.529-matched S glycoprotein, modified to introduce 2 proline residues to stabilise into a prefusion conformation.

31.7.1.3 Dosage, scheduling and packaging

The dosage of mRNA-1273.529 is 50mcg or mRNA in 0.25ml. It is provided as a sterile solution at a concentration of 0.2mg/ml in packs of 1 vial. Each dose is prepared by withdrawing 0.25ml from a vial in a sterile 1ml or equivalent syringe.

31.7.1.4 Storage

Instructions are provided in the IMP handling manual. The injection is stored at -60˚C to -90˚C.

31.8 Safety

31.8.1 Expected Serious Adverse Reactions

There are no expected SAEs for mRNA-1273.529. All SARs identified as probably, possibly or definitely related will therefore be treated as unexpected and reported as SUSARs.

31.8.2 Adverse Events of Special Interest (AESIs)

For the purpose of the sub-study, a separate list of adverse events will be considered AESIs (see table 9 below). The reporting procedure for AESIs will remain the same as during the main study.

Table 9: AESIs for Omicron Variant Specific Fourth Dose Booster sub-study

Medical Concept	Additional Notes
**Anosmia, Ageusia**	• New onset COVID associated or idiopathic events without other etiology excluding congenital etiologies or trauma
**Subacute thyroiditis**	• Including but not limited to events of: atrophic thyroiditis, autoimmune thyroiditis, immune-mediated thyroiditis, silent thyroiditis, thyrotoxicosis and thyroiditis
**Acute pancreatitis**	• Including but not limited to events of: autoimmune pancreatitis, immune-mediated pancreatitis, ischemic pancreatitis, edematous pancreatitis, pancreatitis, acute pancreatitis, hemorrhagic pancreatitis, necrotizing pancreatitis, viral pancreatitis, and subacute pancreatitis<br>• Excluding known etiologic causes of pancreatitis (alcohol, gallstones, trauma, recent invasive procedures)
**Appendicitis**	• Include any event of appendicitis
**Rhabdomyolysis**	• New onset rhabdomyolysis without known etiology such as excessive exercise or trauma

**Acute respiratory distress syndrome (ARDS)**	• Including but not limited to new events of ARDS and respiratory failure.
**Coagulation disorders**	• Including but not limited to thromboembolic and bleeding disorders, disseminated intravascular coagulation, pulmonary embolism, deep vein thrombosis
**Acute cardiovascular injury**	• Including but not limited to myocarditis, pericarditis, microangiopathy, coronary artery disease, arrhythmia, stress cardiomyopathy, heart failure, or acute myocardial infarction
**Acute kidney injury**	• Include events with idiopathic or autoimmune etiologies<br>• Exclude events with clear alternate etiology (trauma, infection, tumor, or iatrogenic causes such as medications or radiocontrast etc)<br>• Include all cases that meet the following criteria<br>○ Increase in serum creatinine by ≥ 0.3 mg/dl (≥26.5 umol/l) within 48 hours;<br>○ OR Increase in serum creatinine to ≥ 1.5 times baseline, known or presumed to have occurred within prior 7 days<br>○ OR Urine volume ≤0.5 ml/ kg/ hour for 6 hours
**Acute liver injury**	• Include events with idiopathic or autoimmune etiologies<br>• Exclude events with clear alternate etiology (trauma, infection, tumor, etc)<br>• Include all cases that meet the following criteria<br>○ > 3-fold elevation above the upper normal limit for ALT or AST<br>○ OR • > 2-fold elevation above the upper normal limit for total serum bilirubin or GGT or ALP
**Dermatologic findings**	• Chilblain-like lesions<br>• Single organ cutaneous vasculitis<br>• Erythema multiforme<br>• Bullous rashes<br>• Severe cutaneous adverse reactions including but not limited to: Stevens-Johnson Syndrome (SJS), Toxic Epidermal Necrolysis (TEN), Drug Reaction with Eosinophilia and Systemic Symptoms (DRESS) and fixed drug eruptions
**Multisystem inflammatory disorders**	• Multisystem inflammatory syndrome in adults (MIS-A)<br>• Multisystem inflammatory syndrome in children (MIS-C)<br>• Kawasaki's disease
**Thrombocytopenia**	• Platelet counts < 150 x10^9<br>• Including but not limited to immune thrombocytopenia, platelet production decreased, thrombocytopenia, thrombocytopenic purpura, thrombotic thrombocytopenic purpura, or HELLP syndrome
**Acute aseptic arthritis**	• New onset aseptic arthritis without clear alternate etiology (eg gout, osteoarthritis, and trauma)

**New onset of or worsening of neurologic disease**	• Including but not limited to<br>o Guillain-Barre Syndrome<br>o Acute disseminated encephalomyelitis (ADEM)<br>o Peripheral facial nerve palsy (Bell’s palsy)<br>o Transverse myelitis<br>o Encephalitis/Encephalomyelitis<br>o Aseptic meningitis<br>o Febrile seizures<br>o Generalized seizures/convulsions<br>o Stroke (Hemorrhagic and non-hemorrhagic)<br>o Narcolepsy
**Anaphylaxis**	• Anaphylaxis as defined per protocol.<br>• Follow reporting procedures in protocol section xxx
**Other syndromes**	• Fibromyalgia<br>• Postural Orthostatic Tachycardia Syndrome<br>• Chronic Fatigue Syndrome (Includes Myalgic encephalomyelitis and Post viral fatigue syndrome)<br>• Myasthenia gravis

31.9 Study Procedures

31.9.1 Schedule of visitation (Omicron Variant Booster Sub-study)

	V1	V2	V3	V4	V5
**Study timeline**	D0	D14	D28	D84	D242
**Study window**	N/A	±3	-7/+3	±14	±28
**Informed consent**	X				
**Safety bloods**	X	X	X		
**Medical history**	X				
**Interim medical history**		X	X	X	X
**Physical examination (as required)**	(X)	(X)	(X)	(X)	(X)
**Urine test (Pregnancy) (if required)**	X				
**COVID-19 vaccination**	X				
**COVID-19 immunogenicity bloods**	X	X	X	X	X
**Diary card review**		X	X		
**SAE/AESI/Medically attended AE check**		X	X	X	X
**Troponin**	X	X			

31.9.2 Blood sampling (Omicron Variant Booster) Immunology Cohort

	V1	V2	V3	V4	V5
**Study timeline**	**D0**	**D14**	**D28**	**D84**	**D242**
**Safety bloods**	X	X	X		
**COVID-19 vaccination**	**X**				
**Exploratory Endpoints**	**PHE**<br>Neutralising Ab<br>Anti-N IgG<br><br>**Nexelis**<br>Anti-spike IgG<br><br>**IMMUNOTEC**<br>ELISpot<br>ICS<br><br>Troponin	**Nexelis**<br>Anti-spike IgG<br><br>**IMMUNOTEC**<br>ELISpot<br>ICS<br><br>Troponin	**PHE**<br>Neutralising Ab<br><br>**Nexelis**<br>Anti-spike IgG<br><br>**IMMUNOTEC**<br>ELISpot	**PHE**<br>Neutralising Ab<br>Anti-N IgG<br><br>**Nexelis**<br>Anti-spike IgG<br><br>**IMMUNOTEC**<br>ELISpot	**PHE**<br>Neutralising Ab<br>Anti-N IgG<br><br>**Nexelis**<br>Anti-spike IgG<br><br>**IMMUNOTEC**<br>ELISpot
**No of tubes & colours**	Up to 20ml Red<br><br>Up to 40ml Green (LiHep)*<br><br>1xFBC (up to 2ml)<br>1xBiochem (up to 5ml)	Up to 20ml Red<br><br>Up to 40ml Green (LiHep)*<br><br>1xFBC (up to 2ml)<br>1xBiochem (up to 5ml)	Up to 20ml Red<br><br>Up to 30ml Green (LiHep)*<br><br>1xFBC (up to 2ml)<br>1xBiochem (up to 5ml)	Up to 20ml Red<br><br>Up to 20ml Green (LiHep)*	Up to 20ml Red<br><br>Up to 20ml Green (LiHep)*
**Total vol. per visit**	Up to 67ml	Up to 67ml	Up to 57ml	Up to 40ml	Up to 40ml
**Total volume (study end)**	At any point in the study, a repeat of safety bloods may be recommended at the clinical discretion of the investigator. This will result in up to an extra 7ml per repeat blood sample.				Up to 271ml

31.9.3 Blood sampling (Omicron Variant Booster) General Cohort with Lithium Heparin

	V1	V2	V3	V4	V5
**Study timeline**	**D0**	**D14**	**D28**	**D84**	**D242**
**Safety bloods**	X	X	X		
**COVID-19 vaccination**	**X**				
**Exploratory Endpoints**	**PHE**<br>Neutralising Ab<br>Anti-N IgG<br><br>**Nexelis**<br>Anti-spike IgG<br><br>**IMMUNOTEC**<br>ELISpot<br><br>Troponin	**Nexelis**<br>Anti-spike IgG<br><br>Troponin	**PHE**<br>Neutralising Ab<br><br>**Nexelis**<br>Anti-spike IgG<br><br>**IMMUNOTEC**<br>ELISpot	**PHE**<br>Neutralising Ab<br>Anti-N IgG<br><br>**Nexelis**<br>Anti-spike IgG<br><br>**IMMUNOTEC**<br>ELISpot	**PHE**<br>Neutralising Ab<br>Anti-N IgG<br><br>**Nexelis**<br>Anti-spike IgG<br><br>**IMMUNOTEC**<br>ELISpot
**No of tubes & colours**	Up to 20ml Red<br><br>Up to 20ml Green (LiHep)*<br><br>1xFBC (up to 2ml)<br>1xBiochem (up to 5ml)	Up to 20ml Red<br><br>1xFBC (up to 2ml)<br>1xBiochem (up to 5ml)	Up to 20ml Red<br><br>Up to 20ml Green (LiHep)*<br><br>1xFBC (up to 2ml)<br>1xBiochem (up to 5ml)	Up to 20ml Red<br><br>Up to 20ml Green (LiHep)*	Up to 20ml Red<br><br>Up to 20ml Green (LiHep)*
**Total vol. per visit**	Up to 47ml	Up to 27ml	Up to 47ml	Up to 40ml	Up to 40ml
**Total volume (study end)**	At any point in the study, a repeat of safety bloods may be recommended at the clinical discretion of the investigator. This will result in up to an extra 7ml per repeat blood sample.				Up to 201ml

31.9.4 Blood sampling (Omicron Variant Booster) General Cohort without Lithium Heparin

	V1	V2	V3	V4	V5
**Study timeline**	**D0**	**D14**	**D28**	**D84**	**D242**
**Safety bloods**	X	X	X		
**COVID-19 vaccination**	**X**				
**Exploratory Endpoints**	**PHE**<br>Neutralising Ab<br>Anti-N IgG<br><br>**Nexelis**<br>Anti-spike IgG<br><br>Troponin	**Nexelis**<br>Anti-spike IgG<br><br>Troponin	**PHE**<br>Neutralising Ab<br><br>**Nexelis**<br>Anti-spike IgG	**PHE**<br>Neutralising Ab<br>Anti-N IgG<br><br>**Nexelis**<br>Anti-spike IgG	**PHE**<br>Neutralising Ab<br>Anti-N IgG<br><br>**Nexelis**<br>Anti-spike IgG
**No of tubes & colours**	Up to 20ml Red<br><br>1xFBC (up to 2ml)<br>1xBiochem (up to 5ml)	Up to 20ml Red<br><br>1xFBC (up to 2ml)<br>1xBiochem (up to 5ml)	Up to 20ml Red<br><br>1xFBC (up to 2ml)<br>1xBiochem (up to 5ml)	Up to 20ml Red	Up to 20ml Red
**Total vol. per visit**	Up to 27ml	Up to 27ml	Up to 27ml	Up to 20ml	Up to 20ml
**Total volume (study end)**	At any point in the study, a repeat of safety bloods may be recommended at the clinical discretion of the investigator. This will result in up to an extra 7ml per repeat blood sample.				Up to 121ml
