# Supplementary material for: Safety, immunogenicity, and reactogenicity of BNT162b2 and mRNA-1273 COVID-19 vaccines given as fourth-dose boosters following two doses of ChAdOx1 nCoV-19 or BNT162b2 and a third dose of BNT162b2 (COV-BOOST): a multicentre, blinded, phase 2, randomised trial
Source: Lancet Infect Dis. 2022 Aug;22(8):1131–41. doi: 10.1016/S1473-3099(22)00271-7 (PMC9084623; doi:10.1016/S1473-3099(22)00271-7)
Supplement: Supplementary appendix 3 [file mmc3.pdf]

THE LANCET
Infectious Diseases

Supplementary appendix 3

This appendix formed part of the original submission and has been peer reviewed. We post it as supplied by the authors.

Supplement to: Munro APS, Feng S, Janani L, et al. Safety, immunogenicity, and reactogenicity of BNT162b2 and mRNA-1273 COVID-19 vaccines given as fourth-dose boosters following two doses of ChAdOx1 nCoV-19 or BNT162b2 and a third dose of BNT162b2 (COV-BOOST): a multicentre, blinded, phase 2, randomised trial. *Lancet Infect Dis* 2022; published online May 9. https://doi.org/10.1016/S1473-3099(22)00271-7.

Statistical Analysis Plan for COV-BOOST Fourth Dose Booster Sub Study

Study Title: A randomised, phase II UK multi-centre study to determine reactogenicity and immunogenicity of booster vaccination against ancestral and novel variants of SARS-CoV-2

Short Title: Evaluating COVID-19 Vaccine Boosters

Sub Study: Fourth Dose Booster

IRAS Project ID: 299180

EudraCT Number: 2021-002175-19

UHS Study Number: RHM MED1781

Based on the Protocol version V9.1 03rd Feb2022

Written by Elaine Shuo Feng[1], Xinxue Liu[1], Leila Janani[2], Victoria Cornelius[2]

[1] Oxford Vaccine Group, Oxford University

[2] Imperial Clinical Trials Unit, Imperial College London

**Chief Investigator:**	Professor Saul Faust<br>NIHR Southampton Clinical Research Facility<br>University Hospital Southampton NHS Foundation Trust,<br>Southampton SO16 6YD
**Sponsor:**	University Hospital Southampton NHS Foundation Trust
**Funder:**	National Institute Health Research (NIHR), supported by the Vaccine Task Force and DHSC.

Document version history log

Version	Date	Author	Summary of changes made
Version 1.0	04/02/2022		
Version 2.0	02/03/2022	Xinxue Liu, Vitoria Cornelius	Update the definition of seropositive population

Approval Signatures

NAME	ROLE	SIGNATURE	DATE
Prof. Saul Faust	Chief Investigator		06.04.2022
Victoria Cornelius	Senior Statistician	Victoria Cornelius Digitally signed by Victoria Cornelius Date: 2022.04.01 18:25:16 +01'00'	
Prof. Robert Read	TSC Chair		06.04.2022

Contents

Abbreviations 4~~3~~
1. Introduction 5
1.1. Study summary 5
2. Sub study objectives 6
3. Design 7~~6~~
3.1. Study Population 7
3.2. Eligibility Criteria 7
3.3. Randomisation and blinding 8~~7~~
3.4. Sample Size 8
3.5. Schedule of Time and events 9
4. Analysis Population/s 10~~11~~
5. Variables of Analysis 12~~13~~
6. Statistical Methodology 13~~14~~
6.1. Baseline demographics 13~~14~~
6.2. Safety Analysis 13~~14~~
6.3. Interim Analysis 13~~14~~
6.4. Final Analysis 14~~15~~
6.5. Subgroup Analysis 14~~15~~
6.6. Sensitivity analysis 15~~16~~
6.7. Missing data 15~~16~~

Abbreviations

AE	Adverse event
AESI	Adverse Event of Special Interest
AR	Adverse reaction
C-19P	COVID-19 Pathway
ChAdOx1	Chimpanzee adenovirus 1
CI	Chief Investigator
CRF	Case Report Form
DSMB	Data Safety Monitoring Board
DHSC	Department of Health and Social Care
EDC	Electronic Data Capture
ELISPOT	Enzyme-linked Immunospot
FBC	Full blood count
GCP	Good Clinical Practice
GMT	Geometric Mean Titre
GP	General Practitioner
ICS	Intracellular Cytokine Staining
ICF	Informed Consent Form
IV	Intravenous
JCVI	Joint Committee on Vaccination and Immunisation
NHS	National Health Service
NIHR	National Institute for Health Research
PBMC	Peripheral blood mononuclear cell
RES	Research Ethics Service

PB	Post-booster
PI	Principal Investigator
REC	Research Ethics Committee
RSI	Reference Safety Information
SAE	Serious Adverse Event
SAM-strips	Synthetic absorbable matrix strips
SAR	Serious Adverse Reaction
SMPC	Summary of Medicinal Product Characteristics
SOP	Standard Operating Procedure
SUSAR	Suspected Unexpected Serious Adverse Reactions
µg	Microgram
Vp	Viral particle
VTF	Vaccine Task Force
WHO	World Health Organisation

1. Introduction

1.1. Study summary

This is a sub-study with the COV-BOOST Trial

Following mass deployment of 3rd dose COVID-19 booster vaccination by the NHS, and the emergence of the Omicron variant which has substantial mutations to the spike protein thought to confer a significant degree of immune escape, the question has arisen over the ability of further doses of COVID-19 vaccination to overcome this immune escape in people over 30 years old. The study question will be whether the peak spike IgG titres at D28 following the 3rd dose booster of BNT162b2 from the COV-BOOST Stage 1 can be matched at D14 following a 4th dose booster of BNT161b2 or mRNA-1273. The eligible participants are shown in context of the original COV-BOOST trial in Figure 1 Study Flowchart.

This sub-study is primarily interested in change over time within an arm. The trial has been randomised to allocate participants from stage one but our interest is not a between arm comparison in this sub-study.

Figure 1 : Study Flowchart

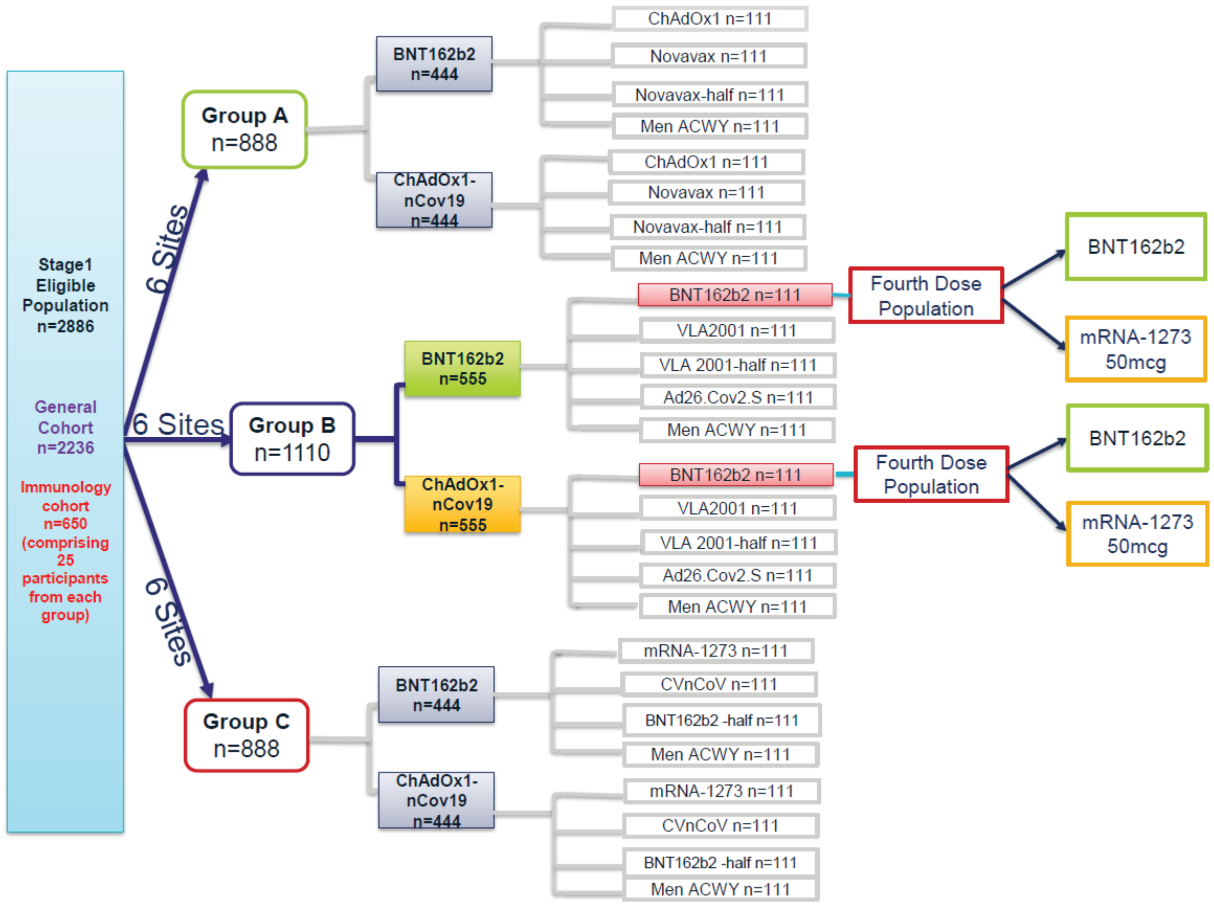

2. Sub study objectives

- To determine the safety and reactogenicity of 4th dose booster vaccination with BNT162b2 and mRNA-1273 50mcg when administered to participants who previously received a 3rd dose of BNT162b2
- To characterise immune responses of 4th dose booster vaccination with BNT162b2 and mRNA-1273 50mcg when administered to participants who previously received a 3rd dose of BNT162b2

3. Design

Participants will be randomised 1:1 to receive either BNT162b2 or mRNA-1273 50mcg. 25 participants in each of the two arms will be included in the immunology cohort which is a subset of participants who receive a more intense follow-up. These may include participants that were not in the immunology cohort for previous visits in the main phase of the trial.

All participants will be monitored for adverse events and have bloods for immunogenicity taken at day 0, 14. Following D14 participants will attend their final visit for the trial 3 months (+/- 14 days) from the time of their fourth dose (Day 0 referenced above).

Safety bloods will be performed at day 0 and 14 including troponin. Participants will be asked to record symptoms in an e-diary in the same manner as the Stage 1 main study, and adverse events will be recorded in the same way.

COVID-19 infection episodes will be recorded as AE/AESIs and initial contact forms with C19 participants will be completed, but no additional visits or sample collection will take place. Participants will be unblinded following the completion of day 84 follow up visits.

3.1. Study Population

Participants from the main study in Group B who received a 3rd dose booster of BNT162b2 (regardless of whether primary course was BNT162b2 or ChAdOx1-nCov19) will be invited to participate These participants include adults aged 30 years or older (including people 75 years and older) who have completed homologous two dose primary course of COVID-19 vaccination (BNT162b2 or ChAdOx1-nCov19) with their first dose of administered in December 2020, January or February 2021.

3.2. Eligibility Criteria

Participants will be eligible for booster vaccination at the Fourth Dose Booster Sub-Study unless they have had a previous severe adverse reaction to mRNA vaccines or have acquired an additional COVID-19 vaccine outside of the study since enrolling. Other medical criteria will be checked prior to immunisation (including diagnosis of cancer, autoimmune conditions, neurological conditions, blood clotting conditions and pregnancy), but will not be considered a contraindication to vaccination unless the investigator feels there is a specific clinical reason to withhold vaccination for the safety of the participant.

Table 1 Treatment Groups for the fourth dose sub-study

Group	Booster	Visits
Fourth Dose Booster	BNT162b2	D0, D14, D84
	mRNA-1273 50mcg	

3.3. Randomisation and blinding

Computer generated randomisation list will be prepared by the study statistician. Participants will be randomised 1:1 to BNT162b2 and mRNA-1273 50 mcg using block randomisation. Random block sizes of 2 or 4 will be used. The randomisation will be stratified by the study sites, prime vaccine schedules (ChAd/ChAd or BNT/BNT), and age group (<70 years and ≥ 70 years). Participants and key study team members will be blinded, statisticians will be unblinded due to the multiple interim analysis and rapid sharing of interim safety data with government expert committees and DMC committees.

3.4. Sample Size

Since this sub-study is nested within the main COV-BOOST trial, there was no formal sample size calculation for the sub-study. The primary analysis is to compare the immune response at D14 post 4th dose and that at D28 post 3rd dose by calculating the geometric mean fold change between the two time-points and 95% confidence interval.

The analysis will be done in all the participants and also be conducted separately for the below four schedules:

- ChAd/ChAd + BNT + BNT
- ChAd/ChAd + BNT + mRNA-1273 50mcg
- BNT/BNT + BNT + BNT
- BNT/BNT + BNT + mRNA-1273 50mcg

For each participant we will calculate the fold change immune responses 14 days post 4th dose to 28 days post 3rd dose and then we summarise by calculating the geometric mean fold change with 95% confidence interval.

3.5. Schedule of Time and events

Table 2: Schedule of visits

	V1	V2	V3*
**Study timeline**	D0	D14	D84
**Study window**	N/A	±3	±14
**Informed consent**	X		
**Safety bloods**	X	X	
**Medical history**	X		
**Interim medical history**		X	X
**Physical examination (as required)**	(X)	(X)	(X)
**Urine test (Pregnancy) (if required)**	X		
**COVID-19 vaccination**	X		
**COVID-19 immunogenicity bloods**	X	X	X
**Diary card review**		X	
**SAE/AESI/Medically attended AE check**		X	X
**Troponin**	X	X	

4. Analysis Population/s

Population	Description
All participants	All participants screened for the trial, to be used for reporting CONSORT diagram
**Safety analysis population**	**Safety modified Per-protocol population (with correction for vaccination error)**<br>• All randomised participants who received a fourth dose of study vaccines, including both seronegative and seropositive populations pre-fourth dose vaccination.<br>• Any vaccination error will be accounted for in this analysis set by assigning participants to the vaccination arm of the vaccine they received<br>• Participants who withdraw from the trial will be included up to the date of their study termination<br>• Logged on to eDiary record for at least one day in 7 days.
**Seronegative Immunogenicity analysis population**	**Seronegative (COVID-19 disease naïve) modified intent-to-treat population**<br>• COVID-19 disease naïve participants who are seronegative ~~at~~ pre-fourth dose (defined as cut off index <1.0 by the Roche Elecsys anti-Sars-CoV-2 assay at- all visits pre-fourth dose~~D0~~)<br>• No self-reported SARS-CoV-2 infection pre-fourth dose (by LFD or PCR)<br>• With no confirmed SARS-CoV-2 infection within 7 days (either pre or post fourth dose)<br>• Randomised and analysed in the assigned vaccine arm regardless of what they received<br>• Participants with endpoint data available
**Seropositive immunogenicity analysis population**	**Seropositive (evidence of previous COVID-19 infection) modified intent-to-treat population (modified ITT)**<br>All randomised participants meeting the below criteria:<br>• Seropositive (laboratory evidence of COVID-19 infection) ~~at~~ pre-fourth dose (defined as cutoff index ≥1.0 by the Roche Elecsys anti-Sars-CoV-2 assay at ~~D0~~any visit pre-fourth dose)<br>• Self-reported SARS-CoV-2 infection pre-fourth dose (by LFD or PCR)<br>• With confirmed SARS-CoV-2 infection within 7 days (inclusive post fourth dose)<br>• Randomised and analysed in the assigned arm for vaccine regardless for what they received

	• With endpoint data available

5. Variables of Analysis

Objectives	Outcome Measures	Timepoint(s)
To determine the reactogenicity of 4th dose booster vaccination with BNT162b2 and mRNA-1273 50mcg when administered to participants who previously received a 3rd dose of BNT162b2	Solicited and unsolicited adverse events<br>Medically attended adverse events	Solicited adverse events: Day 0-7 after immunisation<br>Unsolicited adverse events: Day 0-28 after immunization<br>Medically attended adverse events up to 3 months post immunisation
To characterise immune responses of 4th dose booster vaccination with BNT162b2 and mRNA-1273 50mcg when administered to participants who previously received a 3rd dose of BNT162b2	Anti-spike immunoglobulins, neutralising antibodies, cellular immune response by ICS** and ELISpot**	Day 0, 14, 84

6. Statistical Methodology

6.1. Baseline demographics

Whilst we are not interested in between arm differences, we will present distribution of baseline characteristics for each group to characterise the participants contributing to each of the within arm comparisons.

Baseline characteristics on age, ethnicity, healthcare worker status, comorbidities underlying conditions (respiratory disease, diabetes, cardiovascular events, duration between 1st and 2nd vaccine, between 2nd and 3rd vaccine, and between 3rd and 4th booster) will be summarised by fourth dose vaccine arms, and also stratified by the primary vaccine schedule received.

6.2. Safety Analysis

The safety analysis will be conducted at periodic intervals throughout the study using the most recently available data. The safety analysis population (defined in Section 4) will be used for analysis.

The safety and reactogenicity analysis include multiple outcomes. These comprise of both solicited adverse events over the first 7 days of local and systemic reactions (reactogenicity outcomes), and spontaneously reported (unsolicited) adverse events. Solicited adverse events are named the main COV-BOOST protocol. Adverse events are all events that have been spontaneously reported (unsolicited) or adverse events detected through routine clinical and blood screening tests.

The proportion with *at least one moderate or severe* event systemic and local adverse events over the first seven days will be using a stacked bar chart by severity and day (x-axis) for each solicited event in one row (one row per 4th dose vaccine arm).

An additional view of reactogenicity outcomes will be provided by priming vaccines (ChAdOx n-CoV 19 or BNT), and will be visualised using a stacked bar chart with 4 rows by priming vaccines and 4th dose vaccine arm and radial graphs if useful.

6.3. Interim Analysis

The interim analysis on reactogenicity will be conducted when 7-day diary data become available and the interim analysis on immunogenicity will be carried out when the Day 14 anti-spike IgG data become available to inform the policy making during the pandemic. The aggregated results will be only shared with the relevant parties before publishing the full interim analysis. There will be no formal stopping rules as recruitment will likely complete before the first interim analysis is possible.

The population for interim analysis will follow the Seronegative Immunogenicity analysis population defined in Section 3. The population includes those who have anti-spike IgG data at Day 14 post the 4th booster dose. Additional analysis will also be conducted using Seropositive Immunogenicity analysis population defined in Section 4.

For each participant, the fold change will be calculated by dividing the anti-spike IgG at D14 post 4th dose by the anti-spike IgG at D28 post 3rd dose. The geometric mean of the fold change will be calculated as the antilogarithm of Σ (log10 transformed fold change)/n i.e., as the antilogarithm transformation of the mean of the log10 transformed fold change. Data reported as lower than the detection threshold will be imputed with a value equal to half of the threshold before the transformation. If the geometric mean of the fold change is above 1, it means the 4th dose can boost the anti-spike level higher than that seen at peak post 3rd dose on average.

We will also present the geometric mean concentration of the anti-spike IgG post 4th dose with 95% confidence intervals, as well as the geometric mean of fold change between D14 post 4th dose and pre-4th dose.

The analysis will be done separately for the two vaccine arms (BNT162b2 and mRNA-1273 50mcg) in the populations defined in Section 5. Subgroup analysis will be also conducted by the prime vaccination schedules (ChAD/ChAd or BNT/BNT).

The proportion of missing outcome data is expected to be very low and no exploration for the impact of missing will be performed.

In the final analysis when full follow-up data is available, the analysis will be extended to final analysis immunogenicity outcomes (as defined below).

6.4. Final Analysis

Final analysis of immunogenicity outcomes include:

- Anti-spike immunoglobulins at day 0, 14, 84
- Neutralising antibodies against SARS-CoV-2 at day 0, 14, 84
- Anti-nucleocapsid immunoglobulins at day 0
- Pseudo neutralising antibodies at day 0, 14, 84
- Cellular immune responses by ELISpot at Day 0, 14, 84

Final analysis will use the same methods as defined in Interim analysis when full follow-up data at D84 is available in the populations defined in Section 4

Final analysis will describe the sustainability of immune responses after 4th dose, by the description of the fold change between D84 and D14 for the population defined in Section 5. The absolute level of immune responses at D84 will also be presented by GMC and 95%CI.

6.5. Subgroup Analysis

If the numbers permit (e.g. n>25) we will undertake subgroups analyses within each arm stratified by prime vaccine status, age and sex as below:

- Prime vaccine schedules (ChAdOx n-CoV 19 and BNT)
- Age group (<70 years and ⩾70 years)

- Sex (Male and Female)

6.6. Sensitivity analysis

There is no planned sensitivity analysis as we expect missing data to be minimal.

6.7. Missing data

The level and pattern of the missing data in the baseline variables and outcomes will be reported. The potential causes of any missing data will be investigated and documented as far as possible. If missing data is higher than expected (e.g. >5%) will develop a missing data plan prior to undertaking any final analysis.

2 Amendments to Version 1.0

REFERENCES

- Chis Ster A, Phillips R, Sauzet O, Cornelius V, 2021, Improving analysis practice of continuous adverse event outcomes in randomised controlled trials – a distributional approach, Trials, Vol: 22, ISSN: 1745-62

- Cornelius, V., Cro, S. & Phillips, R. Advantages of visualisations to evaluate and communicate adverse event information in randomised controlled trials. Trials 21, 1028 (2020).

- Moher D, Hopewell S, Schulz KF, Montori V, Gøtzsche PC, Devereaux PJ, Elbourne D, Egger M, Altman DG; CONSORT. CONSORT 2010 explanation and elaboration: updated guidelines for reporting parallel group randomised trials. Int J Surg. 2012;10(1):28-55.

- Sauzet O , Rehse J, Breiding JH. DistdichoR a R Package for the distributional dichotomisation of continuous outcomes. arXiv: Applications. 2018.

- White IR, Thompson SG. Adjusting for partially missing baseline measurements in randomized trials. Statistics in Medicine. 2005;24(7):993-1007.
